# Nearest-Neighbor Effects in Short Unfolded Peptides: An Assessment of Molecular Dynamics Force Fields

**DOI:** 10.1021/acs.jcim.6c00438

**Published:** 2026-06-04

**Authors:** Athul Suresh, Reinhard Schweitzer-Stenner, Brigita Urbanc

**Affiliations:** † Department of Physics, 6527 Drexel University, Philadelphia, Pennsylvania 19104, United States; ‡ Department of Chemistry, Drexel University, Philadelphia, Pennsylvania 19104, United States

## Abstract

Molecular dynamics (MD) is a unique tool for the investigation of intrinsically disordered proteins (IDPs); however, the reliability of MD predictions depends on the accuracy of the underlying force field. Here, we evaluate CHARMM36m, Amber ff14SB, and Amber ff24EXP-GA with respect to their capacity to capture experimentally observed nearest-neighbor (NN) effects on the conformational dynamics of amino acid residues in short unfolded peptides in water. Amber ff24EXP-GA, developed from the parent Amber ff14SB, which is more amino acid-specific than the other two force fields and reproduces intrinsic conformational ensembles of amino acid residues significantly better than the parent force field, captures the NN effects better than Amber ff14SB. Despite a lack of residue specificity in intrinsic conformational ensembles, CHARMM36m, calibrated on global properties of IDPs, reproduces the NN effects on par with Amber ff24EXP-GA. These findings are important for the development of next-generation force fields that reproduce both the residue-specific conformational dynamics and global properties of IDPs.

## Graphical abstract

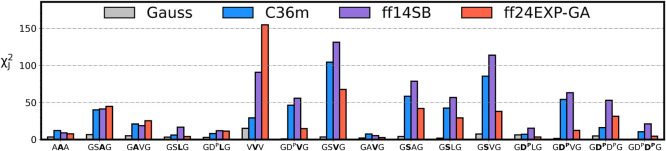

## Introduction

The structure-function paradigm, which states that biological activity requires a stable, unique three-dimensional (3D) protein structure, has been a prominent theme in protein science. Intrinsically disordered proteins (IDPs), which contribute more than one-third to the eukaryotic genome, challenge this notion because they lack a unique native fold.^1^ Instead, IDPs are characterized by unfolded regions that can adopt various distinct conformations upon interaction with multiple partners, making their experimental and computational characterization difficult. Advancing our understanding of IDP structure-function relationships requires the development of accurate and robust tools that can capture entire structural ensembles of IDPs and help us understand their biological significance. Molecular dynamics (MD) offers various tools for exploring conformational free-energy landscapes and dynamics of IDPs and their interactions with binding partners that are relevant to their biological functions. The reliability of conformational dynamics of IDPs derived from MD simulations depends on, and is limited by, the accuracy of the underlying MD force field. Although substantial progress has been made in the development of all-atom MD force fields,^2–4^ several critical limitations in capturing structural and dynamical properties of IDPs by MD remain. In 2015, Rauscher and collaborators evaluated eight different MD force fields on various IDPs and reported significant nonsystematic deviations of chain dimensions, hydrogen bonding, and secondary structure content from the corresponding experimental values derived from small-angle X-ray scattering (SAXS) and NMR spectroscopy.^5^ One of the most concerning outcomes was the observation that switching the force field had a stronger effect on MD-derived structural ensembles of IDPs than their amino acid sequence modifications,^5^ suggesting that, at the least, MD force fields lack amino acid specificity.

The conformational ensembles of amino acid residues in short peptides are directly relevant to IDPs because of their unfolded and solvent-exposed nature. As viable models of IDPs, short unfolded peptides are ideal for bottom-up force field development and benchmarking. Over the past two decades, Schweitzer-Stenner and colleagues have characterized the conformational ensembles of guest residues x in cationic GxG peptides in aqueous solution using a combination of spectroscopic techniques, including solution NMR, vibrational circular dichroism (VCD), Raman, and infrared spectroscopy (IR).^6–8^ These studies have yielded comprehensive residue-specific data consisting of five *J*-coupling constants, polarized Raman, IR, and VCD amide I’ profiles of 14 guest residues (x = G, A, L, V, I, F, Y, D^P^, E^P^, R, C, N, S, and T) in cationic GxG peptides in water. Each residue-specific spectroscopic dataset serves as an input to a Gaussian superposition model that generates a Gaussian Ramachandran distribution, which best reproduces the experimental data.^9^ The overall understanding that emerged from these experiments and the subsequent analysis is that guest residues adopt intrinsic conformational ensembles dominated by polyproline II (pPII) and *β*-strand-like (*β*) conformations, with some polar residues exhibiting, in addition, significant contributions from various turn-like conformations.^7,8^

Empirical guest residue-specific Gaussian Ramachandran distributions can serve as a rigorous benchmark set for the quantitative assessment of MD force fields. Using the above spectroscopic data, Urbanc and collaborators demonstrated that, at the level of individual amino acid residues, i.e., the fundamental building blocks of proteins, commonly used nonpolarizable and polarizable force fields fail to accurately reproduce the experimental intrinsic conformational propensities of guest residues in short unfolded GxG peptides in water.^10–13^ Additive force fields under consideration, such as Amber ff14SB,^14^ Amber ff19SB,^15^ CHARMM36m,^16^ and OPLS-AA/M,^17^ (i) fail to reproduce the polyproline II (pPII) population variability across the guest residues while oversampling antiparallel *β* (a*β*) at the expense of transitional *β* conformations, (ii) inadequately sample turn-like conformations of polar and ionizable guest residues such as N and D^P^, and (iii) overestimate *α*-helical conformations, at variance with the experimental data.^10–12^ Surprisingly, polarizable force fields, AMOEBA^18^ and CHARMM Drude,^19,20^ performed worse than additive CHARMM36m with respect to their ability to capture conformational ensembles of glycine and alanine residues in GGG and GAG peptides, respectively.^13^ The aforementioned experimental data on 14 guest residues in GxG peptides were subsequently utilized as target data in the development of the most recent Amber force field, Amber ff24EXP-GA, which exhibits a higher level of amino acid residue specificity than the parent Amber ff14SB and performs on par with or better than Amber ff14SB and CHARMM36m on longer unfolded protein sequences.^21^

It is, therefore, important to establish whether MD force fields, in particular Amber ff24EXP-GA, capture the effect of nearest-neighbor residues on the Ramachandran distribution of the residue of interest. The classical Flory isolated pair hypothesis assumes that, in the unfolded state, the conformational ensembles of adjacent residues are uncorrelated,^22^ meaning that the nearest-neighbor (NN) effects are negligible. Accumulating evidence on various unfolded systems, ranging from short peptides to denatured proteins, from spectroscopic studies,^23,24^ statistical analyses of coil libraries, and computational studies^25–34^ challenges this assumption. A quantitative assessment of MD force fields with respect to NN interactions is feasible because Schweitzer-Stenner and collaborators extended the aforementioned spectroscopic studies and Gaussian modeling beyond GxG peptides to other unfolded tri-, tetra-, and pentapeptides in water.^35–42^ These studies combined demonstrate that neighboring residues systematically alter the conformational distributions of the residues of interest, with effects that vary depending on the neighbor identity and can accumulate across residues in longer unfolded peptides.

Here, we assess three MD force fields, i.e., CHARMM36m, Amber ff14SB, and Amber ff24EXP-GA, with respect to their capacity to capture experimentally quantified NN effects on the conformational dynamics of amino acid residues in unfolded peptides in water. These three force fields have been optimized in different ways. Optimization of CHARMM36m is rooted in the global conformational properties of longer unfolded peptides and IDPs.^16^ In Amber ff14SB, side-chain dihedral parameters have been derived from quantum mechanical calculations, and backbone dihedral parameters have been further empirically adjusted for alanine residues in short peptides.^14^ Backbone dihedral parameters in Amber ff24EXP-GA have been optimized using experiment-based intrinsic conformational ensembles of glycine and alanine residues in GGG and GAG peptides, respectively.^21^ NN interactions manifest themselves through NN-induced perturbations of the intrinsic conformational ensembles, as visualized by Ramachandran distributions of backbone dihedral (*ϕ*, *ψ*) angles. Whereas glycine neighbors in GxG peptides, due to their minimal interference, allow characterization of intrinsic conformational ensembles of guest residue x, the presence of nonglycine neighbors alters the intrinsic conformational ensemble of the residue of interest, yielding a Ramachandran distribution that can significantly differ from the intrinsic Ramachandran distribution. To this end, we select several amino acid residues of interest in tri-, tetra-, and pentapeptides, for which complete or near-complete sets of experimental data are available.^36,37,39^ Accurate reproduction of these experimental data would indicate that NN effects are adequately accounted for. By evaluating MD force fields that have been developed using different parametrization strategies with respect to NN effects in short unfolded peptides, this study delivers not only a quantitative assessment but also offers insights needed for the development of next-generation force fields that will be able to accurately model the conformational dynamics of IDPs.

## Methods

### Molecular Dynamics Simulations

GROMACS 5.1.2 ^43–49^ is used to perform the MD simulations. The PDB structures of the peptides under this study, i.e., AAA, VVV, GAVG, GD^P^LG, GD^P^VG, GSAG, GSLG, GSVG, GD^P^D^P^G, and GD^P^D^P^D^P^G, are generated using the *Molefacture* plugin within the Visual Molecular Dynamics (VMD) software package.^50^ To mimic the acidic pH used in experiments, all aspartic acid residues are protonated (D^P^) and so are the N-termini of all peptides in this study $\left(-{\mathrm{NH}}_{3}^{+}\right)$ . In addition, the neutral C-terminal capping, −COOH, is used in CHARMM36m, and −CONH_2_ is used in Amber ff14SB and Amber ff24EXP-GA. Each peptide is inserted into a simulation box with cubic geometry, maintaining a minimum distance of 1.2 nm between the peptide atoms and the box edges. In CHARMM36m simulations, the CHARMM-modified TIP3P water is used to solvate the system, whereas in both Amber ff14SB and Amber ff24EXP-GA simulations, the TIP4P-2005 water model is used. All the peptide systems are positively charged, so one chloride ion is added to each system to neutralize it. The steepest descent minimization algorithm is used to minimize the energy of the solvated simulation box until the maximum force on every atom is lower than 1000.0 kJ mol^–1^ nm^–1^. Following the energy minimization, the initial velocities of all atoms in the system are drawn from the Maxwell-Boltzmann distribution at 300 K to match the temperature used in experiments. Then, the system is equilibrated at constant volume and temperature (NVT) for 1 ns using the stochastic V-rescale thermostat.^51^ Subsequently, the system is equilibrated at a constant pressure of 1.0 bar and a temperature of 300 K for 1.0 ns, using the Parrinello-Rahman barostat^52^ with time constants *τ*_*T*_ and *τ*_*P*_ equal to 1.0 and 5.0 ps, respectively, and an isothermal compressibility of 4.5 × 10^–5^ bar^–1^. During both NVT and NPT equilibration steps, the peptide coordinates are restrained using a harmonic force with a force constant of 1000.0 kJ mol^–1^ nm^–1^, and the bonds involving hydrogen atoms are constrained using the LINCS algorithm.^46^ The final production runs are performed in the NPT ensemble, using the exact specifications as those of NPT equilibration, except that the peptides are unrestrained. An integration time step of 2 fs is used in all simulations. The van der Waals interactions are smoothly switched to zero over a distance range of 1.0–1.2 nm, and the short-range electrostatic interactions are cut off at a distance of 1.2 nm. The long-range electrostatic interactions are calculated using the Particle Mesh Ewald (PME)^53^ method with an order of 4 and a grid spacing equal to 0.16 nm. Production runs on tripeptides are 500 ns long, and 25000 time frames, 2 ps apart, from 50–500 ns of each MD trajectory are used in the analysis. Production runs on longer peptides are 1 μs long, and 500000 time frames, 2 ps apart, from 50–1000 ns of each MD trajectory are used in the analysis.

### Analysis

#### Ramachandran Distributions

2D distributions of dihedral angles (*ϕ*, *ψ*) adopted by conformations of the residue of interest are derived from MD production runs using the gmx rama script within GROMACS 5.1.2. Normalized 2D distributions are calculated with a bin size of 2° × 2°, resulting in 180 × 180 = 32400 bins in the range −179, −177,..., 177, 179 along the *ϕ* and *ψ* coordinates to facilitate a direct comparison to the experiment-based Ramachandran distributions predicted by the Gaussian model.

#### Definitions of Mesostates

To better visualize the different conformational ensembles adopted by the amino acid residues in short peptides in Ramachandran distributions, we demarcate the Ramachandran space into distinct regions, referred to as mesostates (Figure 1). Seven distinct mesostate regions in the Ramachandran space are used here, consistent with the definitions used in previous studies:^12,21^

1 Polyproline II, pPII (−90° < *ϕ* < −42°, 100° < *ψ* < 180°)
2 Antiparallel *β*-strand, a*β* (−180° < *ϕ* < −130°, 130° < *ψ* < 180°)
3 *β* transition region between a*β* and pPII, *β*t (−130° < *ϕ* < −90°, 130° < *ψ* < 180°)
4 Parallel *β*-strand, p*β*-strand (−130° < *ϕ* < −90°, 100° < *ψ* < 130°)
5 Extended conformations in the lower left region of the Ramachandran space, *ε* (−180° < *ϕ* < −20°, −180° < *ψ* < −150°)
6 Turn-like structures:
  a Type I/II’ *β*_*i*+2_ (−110° < *ϕ* < −30°, −20° < *ψ* < 20°)
  b Type I’/II *β*_*i*+2_ (50° < *ϕ* < 110°, −20° < *ψ* < 20°)
  c Type I/III’ *β*_*i*+1_ (−80° < *ϕ* < −30°, −50° < *ψ* < −20°)
  d Type I’/III’ *β*_*i*+1_ (50° < *ϕ* < 110°, −20° < *ψ* < 20°)
  e Inverse *γ* (−80° < *ϕ* < −30°, 30° < *ψ* < 80°)
  f Classical *γ* (30° < *ϕ* < 80°, −70° < *ψ* < −30°)
  g Asx (70° < *ϕ* < 110°, 75° < *ψ* < 170°).

**Figure 1:**
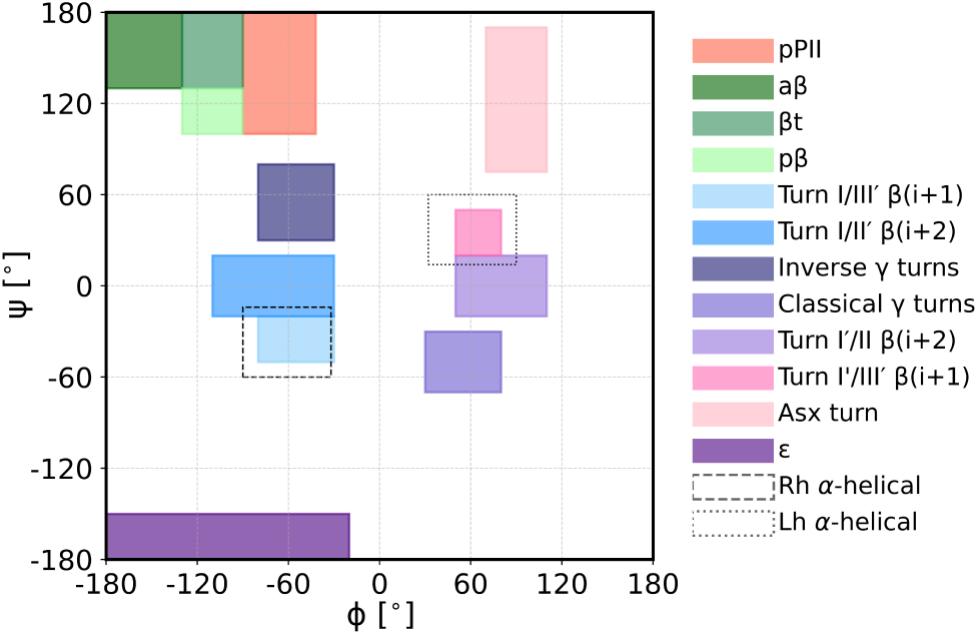
Definitions of mesostates in the Ramachandran space.

It is important to note that the secondary structure conformations of the guest residues in short unfolded peptides, in the context of this study, do not correspond to traditional secondary structures, which require multiple contiguous residues to adopt dihedral angles from a localized region of Ramachandran space. Instead, we refer here to mesostates as conformational ensembles defined by the dihedral angles of a single guest residue of interest.

#### Hellinger Distance

We quantify the degree of similarity between Ramachandran distributions using the Hellinger distance, a metric that measures the extent of overlap between two probability distributions.^32^ Assuming that the Ramachandran space is divided into N_*ϕ*_ and N_*ψ*_ bins along the *ϕ*- and *ψ*-axes, respectively, the Hellinger distance H­(P_A_, P_B_) between distributions P_A_ and P_B_ is calculated as

1 $$H(P_{A},P_{B})=\sqrt{\frac{1}{2}\overset{N_{\phi }}{\underset{i=1}{\sum }}\overset{N_{\psi }}{\underset{j=1}{\sum }}{\left[\sqrt{P_{B}(\phi_{i},\psi_{j})}-\sqrt{P_{A}(\phi_{i},\psi_{j})}\right]}^{2}}$$

where P_A_(*ϕ*_*i*_, *ψ*_*j*_) and P_B_(*ϕ*_*i*_, *ψ*_*j*_) are the probabilities corresponding to bin (*i*, *j*) in the Ramachandran space.

The Hellinger distance values range from 0 to 1, where 0 indicates identical distributions and 1 indicates completely dissimilar distributions. We interpret the Hellinger distance values using previously established criteria adopted by Schweitzer-Stenner and Toal:^54^ H ≤ 0.1 signifies very similar distributions, 0.1 < H ≤ 0.25 indicates moderate similarity between the two distributions, 0.25 < H ≤ 0.4 corresponds to moderately dissimilar distributions, and H ≥ 0.4 indicates very dissimilar distributions.

Importantly, the Hellinger distance is sensitive to both the location and the dimensions of conformational basins in the Ramachandran space, rather than just population differences within fixed regions. This provides complementary information to the mesostate population analysis. Whereas the mesostate populations measure occupancies of specific regions, the Hellinger distance captures, in addition to the occupancies, shifts in the location of the conformational basins in the *ϕ*–*ψ* space.

#### MD-Derived NMR *J*-Coupling Constants

The NMR *J*-coupling constants , ^3^ *J*(H^N^, C′), ^3^ *J*(H^C*α*^, C′), ^3^ *J*(H^N^, C_*β*_), and ^1^ *J*(N, C_*α*_) that have been determined experimentally are also calculated from the MD-derived and Gaussian Ramachandran distributions. The first four ^3^ *J* coupling constants are sensitive to the backbone dihedral angle *ϕ*, whereas ^1^ *J*(N, C_*α*_) depends only on the backbone dihedral angle *ψ*. The *J-*coupling constant that depends on the dihedral angle *θ* can be calculated using the Karplus equation:^55^

2 $$J(\theta )=A\;\cos^{2}\left(\theta -\theta_{0}\right)+B\;\cos\left(\theta -\theta_{0}\right)+C$$

and the final *J*-coupling constant corresponding to the Ramachandran distribution *P*(*ϕ*, *ψ*) is derived by taking an ensemble average of the function in eq 2 over the *ϕ*–*ψ* space as

3 $$\langle J\rangle =\int_{-\pi }^{\pi }\int_{-\pi }^{\pi }P(\phi ,\psi )J(\phi ,\psi )d\phi d\psi$$

where *A*, *B,* and *C* are the Karplus parameters, obtained mostly from experimental values of *J*-coupling constants of residues in folded proteins from available X-ray crystallographic and/or NMR-based structures, and *J*(*ϕ*, *ψ*) is a general form of a *J*-coupling constant that can depend on either or both backbone dihedral angles. In our case, four *J*-coupling constants (^3^ *J*) depend on *ϕ* alone, and one *J*-coupling constant (^1^ *J*) depends on *ψ* alone, as implied by eq 2. For the alanine residue, Karplus constants obtained from density functional theory calculations are also available.^56^ In line with earlier work,^6,7,36^ we utilize the Karplus parameters and phases *θ*_0_ from Hu and Bax^57^ for all *ϕ*-dependent ^3^ *J*-coupling constants. For ^1^ *J*(N,C_*α*_), we use the Karplus parameters reported by Wirmer and Schwalbe.^58^ The deviation between calculated and experimental values is quantified using the absolute value of their differences as well as a reduced $\chi_{J}^{2}$ function:

4 $$\chi_{J}^{2}=\frac{1}{N}\overset{N}{\underset{i=1}{\sum }}\frac{{\left(J_{i,\exp}-J_{i,\mathrm{calc}}\right)}^{2}}{s_{i}^{2}}$$

where *J*_*i*_,_exp_ and *J*_*i*_,_calc_ denote experimental and calculated *J*-coupling constant values (obtained by using eqs 3 and 2, respectively), *N* is the number of *J*-coupling constants, and s_*i*_ are statistical uncertainties, i.e., a combination of experimental errors^6^ and errors due to the reported errors in the estimation of the Karplus parameters.^57,58^ Statistical uncertaintiess_i_ are calculated as ensemble averages of the following function s̅_*i*_(*θ*)­

5 $${\mathrm{s̅}}_{i}(\theta )=\sqrt{s_{A_{i}}^{2}\cos^{4}(\theta -\theta_{0,i})+s_{B_{i}}^{2}\cos^{2}(\theta -\theta_{0,i})+s_{C_{i}}^{2}+s_{J_{i}}^{2}}$$

where $s_{A_{i}}$ , $s_{B_{i}}$ , and $s_{C_{i}}$ are the statistical uncertainties associated with the three Karplus parameters, *θ* and *θ*_0,*i*_ are the relevant dihedral angle and phase associated with the *J*-coupling constant *J_i_*, respectively, and s_Ji_ is the corresponding experimental uncertainty. The statistical uncertainties of the three Karplus parameters are available for all *J*-coupling constants of residues in peptides investigated in this work, except for ^1^ *J*(N, *C*_*α*_).^57,58^

#### Empirical Fitting of Ramachandran Distributions Using Gaussian Modeling

The Gaussian decomposition model represents residue-specific conformational ensembles as two-dimensional Gaussian subdistributions corresponding to known mesostates, such as pPII, p*β*, a*β*, t*β*, helical, and various turn-like states (Figure 1) within the Ramachandran space.^9^ The overall Ramachandran distribution, i.e., the probability distribution in the phase space of the two backbone dihedral angles, *P*(*ϕ*, *ψ*), is expressed as a weighted sum of Gaussian subdistributions:

6 $$P(\phi ,\psi )=\underset{i}{\sum }\chi_{i},G_{i}(\phi_{i,m},\psi_{i,m},\sigma_{i,\phi },\sigma_{i,\psi })$$

where *χ*_*i*_ denotes the mole fraction of the *i*-th mesostate, and G_*i*_ is a two-dimensional Gaussian distribution centered at *ϕ*_*i*_,m and *ψ*_*i*_,m with half-widths *σ*_*i*_,*_ϕ_* and *σ*_*i*_,*_ψ_*. For a given dihedral angle distribution, *P*(*ϕ*, *ψ*), *J*-coupling constants are calculated via the Karplus equations using parameters reported previously.^36^ The parameters of the Gaussian model (the positions and half-widths of the Gaussian subdistributions) are optimized to achieve the best agreement between calculated and experimental *J*-coupling constants.^59^

In this work, the parameters of the Gaussian Ramachandran distributions of the central alanine residue in GAG and AAA are taken from Zhang and collaborators^10^ and are based on the Gaussian model analysis reported in earlier work.^6,60^ Mole fractions for the central residues in GVG, GLG, and GSG peptides are reproduced from Andrews and collaborators^12^ with permission from the Royal Society of Chemistry, whereby the corresponding positions of Gaussian subdistributions have been reported by Hagarman and collaborators.^6^ All Gaussian parameters for the central valine residue in the VVV peptide are taken from Schweitzer-Stenner.^9^ We here use the Gaussian Ramachandran distribution parameters for the protonated aspartic acid residue in GD^P^G peptide reported by Milorey and collaborators.^39^ The experimental data for guest residues x and y in GxyG peptides, obtained by Toal and collaborators,^37^ have been reanalyzed with a higher-resolution Gaussian model. Toal *et al.* used a reduced space of the Ramachandran plot by solely considering the regions that were expected to be significantly populated, with a lower resolution of 6° and 8°,^37^ which could lead to under- or overestimation of *J*-coupling constants if subpopulations are positioned in a region where the Karplus curves of *J*-coupling constants have a large slope. Thus, Schweitzer-Stenner repeated the analysis of experimental data using the protocol reported by Milorey and collaborators.^61^ Briefly, the full Gaussian model was solely used for nonlinear least-square fitting of eq 6 to a set of five experimental scalar *J*-coupling constants of the respective guest residues x and y in GxyG peptides (i.e., the *ϕ*-dependent J3(HN,HCα) , ^3^ *J*(H^N^, C′), ^3^ *J*(H^C*α*^, C′), ^3^ *J*(H^N^, C_*β*_), and the *ψ*-dependent ^1^ *J*(N, C_*α*_), which were carried out using the Matlab program. During fitting, the statistical weights were used as free parameters, whereas the positions and widths of these distributions were mostly fixed, except in a few cases, in which fitting was repeated with slightly changed positions and widths. Ramachandran distributions were calculated with a resolution of 2°. Subsequently, amide I’ profiles in isotropic Raman, anisotropic Raman, FTIR, and vibrational circular dichroism spectra of the investigated GxyG peptides were simulated with a truncated model constructed based on the Gaussian analysis of the *J*-coupling constants, as described in detail elsewhere.^61^ These steps were reiterated until the best fit to all data was obtained. The reductionist approach to the amide I’ data was necessary to reduce the computational costs of the analysis. The Gaussian Ramachandran distribution parameters for all aspartic acid residues in GD^P^D^P^G and GD^P^D^P^D^P^G peptides are taken from Milorey *et al.* ^39^ without any modifications.

## Results and Discussion

Availability of comprehensive sets of experimental data on guest residues x in GxG peptides in water^6–8,36^ has enabled the evaluation of several additive and two polarizable MD force fields with respect to their ability to capture intrinsic conformational ensembles of amino acid residues in water.^10–13,62^ These studies combined showed that even the best MD force fields, such as Amber ff14SB,^14^ OPLS-AA/M,^17^ and CHARMM36m,^16^ fail to capture the amino acid-residue specificity of intrinsic conformational ensembles as quantified by experiment-based residue-specific Gaussian Ramachandran distributions. Backbone dihedral parameters for guest glycine and alanine residues in GGG and GAG peptides, respectively, have been recently optimized within Amber ff14SB against the experimental data.^21^ The resulting force field, Amber ff24EXP-GA, combined with TIP4P-2005 water,^63^ predicts intrinsic conformational ensembles of 15 guest residues in GxG peptides in water that agree with experimental data significantly better than those derived from the parent force field and show significantly more amino acid residue specificity than Amber ff14SB or CHARMM36m.^21^

We here ask to what extent Amber ff24EXP-GA with the TIP4P-2005 water model captures NN effects on conformational ensembles of the residue of interest in short unfolded peptides and compare its performance to Amber ff14SB with the TIP4P-2005 water model and CHARMM36m with its intrinsic TIP3P water model. The selection of test systems, i.e., short homopeptides (x**x**x and GxxxG) and heteropeptides (G**xy**G), is guided by the availability of respective comprehensive experimental data previously reported by Schweitzer-Stenner and collaborators.^37,39,40^ The Gaussian Ramachandran distributions of residues of interest (see *Methods*) serve as benchmarks by providing a visual counterpart to experimental data. It is important to note that we use MD-derived Ramachandran distributions to calculate *J*-coupling constants and amide I’ profiles to facilitate a direct comparison to experimental data, which is independent of Gaussian modeling. Experimental data on the central residues in cationic A**A**A and V**V**V peptides were reported by Graf and collaborators.^36^ Our choice of G**xy**G peptides as model systems to explore NN interactions between the residues x and y is based on experimental data reported by Toal et al., who determined NMR *J*-coupling constants and the amide I’ profiles of selected guest residues (shown in bold font) in cationic G**D^p^** yG, G**S**yG, Gx**L**G, and Gx**V**G peptides.^37^ Milorey and collaborators reported experimental data on all protonated aspartic acid residues in cationic GD^p^D^p^G and GD^p^D^p^D^p^G peptides.^40^ While the experimental data for x and y guest residues of interest in cationic GxyG peptides were taken from Toal and collaborators,^37^ the corresponding Gaussian model parameters were rederived using a higher-resolution model,^61^ as described in *Methods*. In the following, we assess Amber ff14SB, Amber ff24EXP-GA, and CHARMM36m with respect to their capacity to reproduce NN effects on conformational ensembles of alanine, leucine, valine, serine, and aspartic acid.

### Nearest-Neighbor Effects on Alanine

Here, we compare the conformational ensembles of the alanine residue (**A**) in cationic G**A**G, A**A**A, GS**A**G, GA**V**G peptides in water. The empirical Gaussian Ramachandran distribution of alanine in the cationic G**A**G peptide is characterized by a high pPII content of 58%. The pPII content in the central alanine residue in cationic A**A**A is even higher, exceeding 75%, which demonstrates that alanine residues as NNs enhance the characteristic conformational ensemble, i.e., pPII conformations.^10,35,36,60^ The pPII content of the alanine residue in G**A**yG and Gx**A**G peptides decreases if the neighboring residues x and y possess intrinsic conformational ensembles distinct from that of the alanine residue.^37^ This can be observed in the Gaussian Ramachandran distributions in Figure 2A as well as in the corresponding mesostate populations in Figure 2B. The presence of the polar serine residue in GS**A**G peptide significantly reduces the pPII content of the alanine residue.^37^ This decreased pPII content is concomitant with an increase in the *β*-strand content and turn-like conformations.^37^ The valine residue, with its intrinsic preference for *β*-strand-like conformations,^6^ also affects the conformational propensity of the alanine residue in the cationic G**A**VG peptide. When the valine residue is the NN to alanine in G**A**VG peptide, the *β*-strand population of the alanine residue increases at the expense of the pPII population.^37^ MD simulations of the four peptides in the three force fields (CHARMM36m, Amber ff14SB, and Amber ff24EXP-GA) are conducted as described in Methods. The MD-derived Ramachandran distributions and the corresponding mesostate populations are shown in Figure 2A and Figure 2B, respectively, alongside the Gaussian model results. The numerical values of all mesostate populations are also reported in Table S1.

**Figure 2:**
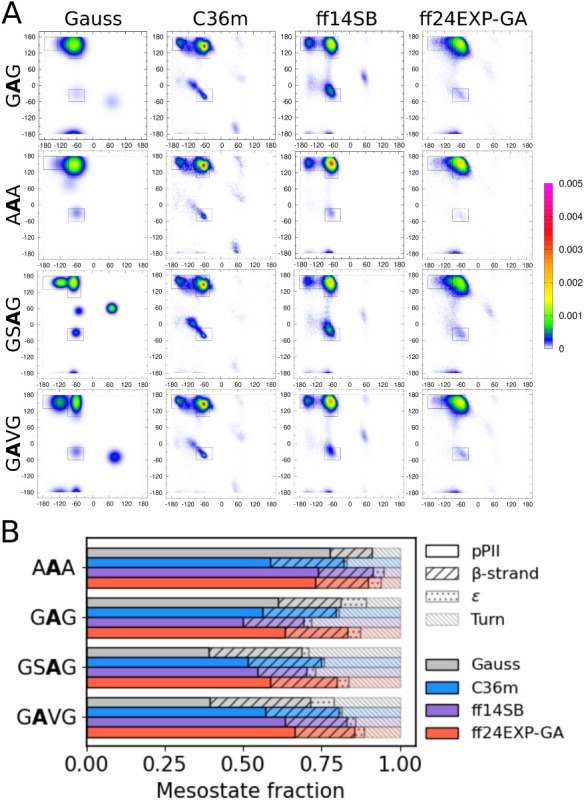
(A) Ramachandran distributions and (B) mesostate populations of alanine residue in G**A**G, A**A**A, GS**A**G, and G**A**VG peptides obtained within the Gaussian model and derived from CHARMM36m, Amber ff14SB, and Amber ff24EXP-GA simulations. Data pertaining to G**A**G Ramachandran distributions for the Gaussian model are taken from Zhang and collaborators (Copyright 2020, American Chemical Society).^10^ MD-derived Ramachandran distributions for the alanine residue in GAG are reproduced from ref 12 with permission from the Royal Society of Chemistry or taken from Suresh and collaborators (Copyright 2025, American Chemical Society).^21^

None of the three MD force fields reproduces significant changes in the conformational ensembles of alanine residues in A**A**A, GS**A**G, and G**A**VG peptides relative to the conformational ensembles of the alanine residue in G**A**G peptide very accurately, as can be observed by comparing the Gaussian and MD-derived Ramachandran distributions (Figure 2A). Figure 2B and Table S1 provide a more quantitative comparison of the mesostate populations, which confirms that the three MD force fields capture NN effects on the alanine residue to a very limited extent. The most notable exception is the cooperative effect when the central alanine’s pPII content increases in the presence of two flanking neighbors in A**A**A. This reinforcement of the intrinsic pPII population in A**A**A relative to G**A**G is captured by both Amber force fields, with Amber ff24EXP-GA showing the best agreement with the Gaussian model. Of the three MD force fields, CHARMM36m performs the worst in capturing the enhanced pPII content of the central alanine residue in A**A**A relative to G**A**G. In contrast, the “anticooperative” reduction in the pPII population of the alanine residue due to unlike neighboring residues, such as serine and valine, is not captured by any MD force fields (Figure 2B and Table S1). In GS**A**G, CHARMM36m shows a depleted pPII population, accompanied by an equal enhancement in the turn population. In contrast, *β*-strand basin population remains unchanged and is comparable to the intrinsic population of the alanine residue in G**A**G. Amber ff14SB results show that the pPII content modestly increases, whereas the *β*-strand and turn populations decrease. In Amber ff24EXP-GA, which is parameterized based on alanine’s intrinsic conformational propensities, the pPII mesostate decreases while the *β*-strand and turn populations increase. For the valine residue as alanine’s NN in G**A**VG peptide, none of the three MD force fields captures the reduced pPII, increased *β*-strand, and increased turn mesostate populations relative to alanine’s intrinsic preferences, as predicted by the Gaussian model (Figure 2B and Table S1).

The Hellinger distance, which measures the degree of similarity/difference between two Ramachandran distributions, is sensitive to differences in the positions of basins as well as their populations, making it a compelling quantity for studying NN effects.^54^ Figure S1 shows Hellinger distance matrices that quantify the NN effects for alanine in the Gaussian model (Figure S1A) and the three MD force fields (Figure S1B-D). Hellinger distance values are split into four categories, as explained in *Methods*. Relative to the Gaussian Ramachandran distribution of the alanine residue in G**A**G, the Ramachandran distributions of alanine residues in A**A**A, GS**A**G, and G**A**VG peptides show moderate to high dissimilarity, with the distribution of the alanine residue in GS**A**G deviating from that of alanine in G**A**G the most. Upon examining the Gaussian Ramachandran distribution of alanine in GS**A**G peptide, one can observe a new type I’/III’*β*_*i*+1_ turn conformational basin, which contributes to the significant Hellinger distance between the Ramachandran distributions of alanine in G**A**G and GS**A**G. Based on the Hellinger distance analysis, the three force fields fail to capture the effects of NN residues, and the Ramachandran distributions do not exhibit as large dissimilarities as observed in the experiment-based Gaussian model. Of the three MD force fields, Amber ff24EXP-GA shows the largest dissimilarities and is thus the most in line with the Gaussian model, followed by Amber ff14SB, whereas CHARMM36m predicts the most similar Ramachandran distributions, thereby deviating the most from the Gaussian model predictions (Figure S1B-D).

The most rigorous comparison of MD predictions to experimental data, comprising several NMR *J*-coupling constants, can be done by using MD-derived Ramachandran distributions to directly calculate the *J*-coupling constants, followed by the calculation of the respective relative $\chi_{J}^{2}$ value, defined by eqs 2–5 and described in *Methods*. Figures S2, S3, and S4 show a comparison of calculated and experimental *J*-coupling constants, and the respective $\chi_{J}^{2}$ values for the alanine residue in A**A**A, GS**A**G, and G**A**VG peptides, respectively (see also numerical values in Table S2). Of the three MD force fields, the *J*-coupling constants of the central alanine in A**A**A are best reproduced by Amber ff24EXP-GA, followed by Amber ff14SB, whereas CHARMM36m deviates from the experimental data the most (Figure S2-vi). Overall, Amber ff24EXP-GA captures the central alanine’s conformational preferences in A**A**A better than the other two force fields. Results of MD simulations for the alanine residue in GS**A**G peptide demonstrate poor agreement with experimental *J*-coupling constants, yielding consistently high $\chi_{J}^{2}$ values for all three force fields (Figure S3). The observed bias of MD force fields for pPII over *β*-strand conformations contradicts experimental observables, in particular with respect to the *ϕ*-dependence of the Ramachandran distribution, associated with ^3^ *J*(H^N^, H^C*α*^), ^3^ *J*(H^N^, H^C*α*^), ^3^ *J*(H^N^, C_*β*_), and ^3^ *J*(H^C*α*^,C′) values. The *ψ*-dependence of MD-derived Ramachandran distributions, as reflected in the close correspondence between experimental and MD-derived ^1^ *J*(N, *C*_*α*_) values, is relatively satisfactory, possibly due to an increased turn propensity of the alanine residue in GS**A**G peptde compared to that in G**A**G peptide, which primarily affects *ψ*-coordinate (Figure 2A). The three MD force fields are associated with large $\chi_{J}^{2}>40$ values, with CHARMM36m predicting the lowest $\chi_{J}^{2}$ value, followed by Amber ff14SB, and Amber ff24EXP-GA. The agreement between MD-derived and experimental *J*-coupling constants for the alanine residue in G**A**VG peptide is also poor within the three MD force fields, as reflected in Figure S4. While Amber ff14SB slightly outperforms the other two force fields with respect to reduced $\chi_{J}^{2}$ values, all three force fields predict rather large $\chi_{J}^{2}$ values of ∼20.

Thus, while MD force fields capture increased pPII conformations of the central alanine residue in A**A**A relative to the respective intrinsic ensemble, they do not reproduce the effects of unlike NN residues, such as S and V, with a high intrinsic *β*-strand preference, on the conformational ensemble of the alanine residue.

### Nearest-Neighbor Effects on Leucine

An experimental study by Hagarman and collaborators revealed that the intrinsic conformational ensemble of the leucine residue in the cationic GLG peptide is dominated by balanced pPII and *β*-strand populations.^6^ Toal *et al.* demonstrated that NN interactions do not strongly affect the conformational preferences of the leucine residue.^37^ Acquiring and analyzing MD trajectories of GSLG and GD^P^LG peptides in the three MD force fields, we here compare the conformational dynamics of the leucine residue in GS**L**G and GD^P^ **L**G peptides to its intrinsic dynamics in G**L**G peptide. Figure 3A shows the Gaussian and MD-derived Ramachandran distributions of the leucine residue in G**L**G, GS**L**G, and GD^P^ **L**G peptides. Visual inspection of Figure 3A reveals that the Gaussian model predicts more diverse Ramachandran distributions than any of the three force fields. As reported by Schweitzer-Stenner, the context-induced changes in the conformational ensemble of the leucine residue consist of moderate changes in the Gaussian weights, whereas the resulting Ramachandran distributions differ in the position of the *β*-strand-like basin.^61^ Mesostate populations in Figure 3B show that, to some extent, all three force fields qualitatively capture the NN effects on the leucine residue (see also Table S3). Whereas the Gaussian model-based Hellinger distances in Figure S5A exhibit very high to moderate dissimilarities among the three peptides, the corresponding MD-derived Hellinger distances in Figure S5B-D are moderately similar to moderately dissimilar. Of the three MD force fields, Amber ff24EXP-GA predicts the largest Hellinger distances within the moderately dissimilar range.

**Figure 3:**
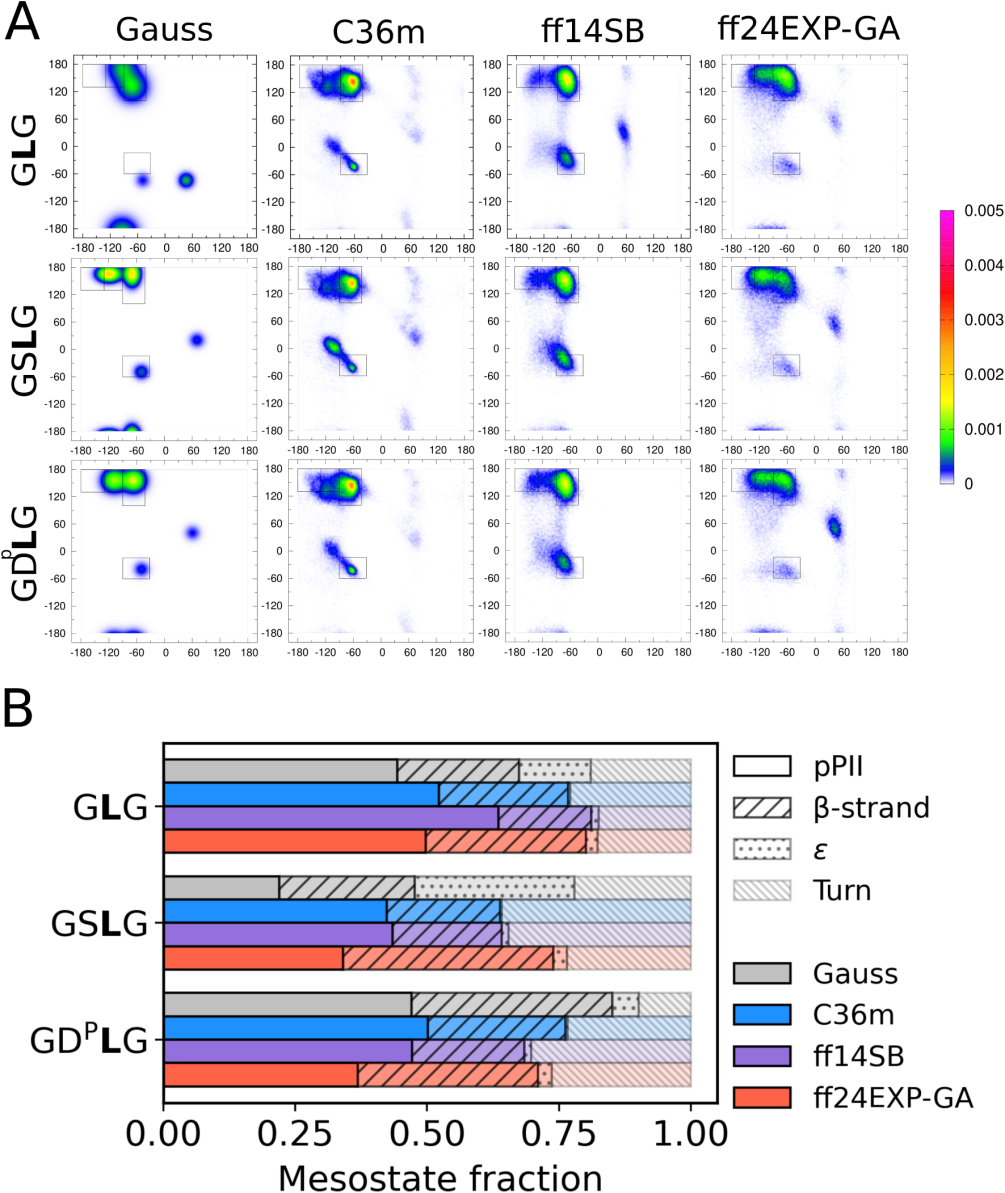
(A) Ramachandran distributions and (B) mesostate populations of leucine residue in G**L**G, GS**L**G, and GD^P^ **L**G peptides obtained within the Gaussian modeling and derived from CHARMM36m, Amber ff14SB, and Amber ff24EXP-GA simulations. Data pertaining to G**L**G Ramachandran distributions for the Gaussian model are reproduced from Andrews et al.^12^ with permission from the Royal Society of Chemistry, and the corresponding MD-derived Ramachandran distributions are taken from Suresh et al. (Copyright 2025, American Chemical Society).^21^

Figures S6 and S7 show a comparison between the calculated and experimental *J*-coupling constants of the leucine residue in GS**L**G and GD^P^ **L**G peptides (see also Table S4). Evaluation of leucine residue ensembles in GS**L**G using the reduced $\chi_{J}^{2}$ values reveals that Amber ff24EXP-GA produces the lowest value, followed by CHARMM36m, the Gaussian model, and Amber ff14SB, indicating that in this case, the force fields perform better or at least comparably to the Gaussian model. In the case of the leucine residue ensembles in GD^P^ **L**G peptides, MD force fields are less accurate than the Gaussian model with a reduced $\chi_{J}^{2}<5$ . CHARMM36m exhibits the best performance among the three force fields with $\chi_{J}^{2}<10$ , followed by Amber ff24EXP-GA and Amber ff14SB with $\chi_{J}^{2}$ values around 10. Thus, the NN effects on the leucine residue are reproduced by MD force fields somewhat better than the effects of unlike NNs on the alanine residue. This observation may not be surprising because the NN effects on the leucine residue are not very strong, and most MD force fields tend to produce similar Ramachandran distributions for distinct residues due to their limited amino acid residue specificity.^12^

### Nearest-Neighbor Effects on Valine

The intrinsic conformational ensemble of the valine residue in G**V**G peptide, derived from the experiment-based Gaussian model, is dominated by *β*-strand, followed by pPII and turn-like conformations.^6,9^ Experimental data-based analysis shows that the *β*-strand conformational ensemble becomes even more dominant for the central valine residue in V**V**V peptide, demonstrating that like NNs shift conformational preferences even more toward the preferred *β*-strand state, while the fraction of pPII conformations is relatively minor^9,36^ (Figure 4A). The most recent Gaussian model analysis of experimental data, reported by Toal *et al.* ^37^ showed that the incorporation of turn-promoting residues, such as aspartic acid and serine in GD^P^ **V**G and GS**V**G peptides, respectively, results in a reduction of pPII and an enhancement of *β*-strand content of the valine residue.^61^ The alanine residue in GA**V**G peptide increases the valine residue’s populations of pPII and *β*-strand basins compared to the conformational preferences of the valine residue in G**V**G peptide. Here, we examine the conformational ensembles of the central valine residue in V**V**V, GD^P^ **V**G, GS**V**G, and GA**V**G peptides using three MD force fields, as described in the Methods. The Gaussian and MD-derived Ramachandran distributions of the central valine residue in G**V**G, V**V**V, GD^P^ **V**G, GS**V**G, and GA**V**G peptides are displayed in Figure 4A with the respective mesostate populations shown in Figure 4B (see also Table S5).

**Figure 4:**
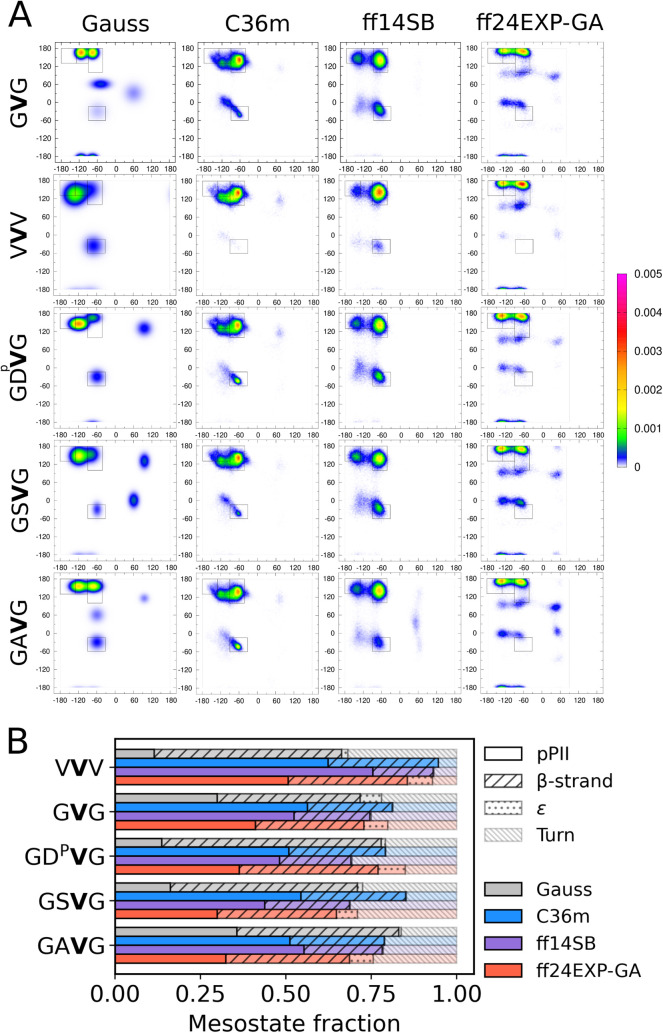
(A) Ramachandran distributions and (B) mesostate populations of valine residue in G**V**G, V**V**V, GD^P^ **V**G, GS**V**G, and GA**V**G peptides obtained within the Gaussian model and derived from CHARMM36m, Amber ff14SB, and Amber ff24EXP-GA simulations. The Gaussian Ramachandran distribution for V**V**V is taken from Schweitzer-Stenner (Copyright 2009, American Chemical Society).^9^ The Gaussian Ramachandran distribution for G**V**G is reproduced from ref 12 with permission from the Royal Society of Chemistry, and the corresponding MD-derived Ramachandran distributions are taken from Suresh and collaborators (Copyright 2025, American Chemical Society).^21^

The comparison of Ramachandran distributions of the valine residue in Figure 4A shows that, while the Gaussian model predicts substantial differences in valine’s conformational preferences across the five peptides, as described above, MD force fields result in Ramachandran distributions that are relatively insensitive to the NNs. Although MD force fields result in slightly different Ramachandran distributions for the central valine residue in G**V**G and V**V**V peptides, the mesostate population changes are not in quantitative agreement with those predicted by the Gaussian model (Figure 4B). Of the three force fields, CHARMM36m alone predicts a slight increase in valine’s *β*-strand population at the expense of its pPII population in V**V**V relative to G**V**G peptide, but this increase is not sufficiently large (see also Table S5). A decrease in the pPII population of the valine residue in GD^P^VG and GS**V**G peptides relative to its intrinsic pPII propensity, due to turn-promoting residues D^P^ and S, is captured moderately well by the three force fields. The conformational ensemble of the valine residue in GA**V**G peptide with comparable pPII and *β*-strand populations, is captured well only in Amber ff24EXP-GA (see Figure 4B and Table S5).

Figure S8 shows the Hellinger distances between pairs of Ramachandran distributions for valine residues obtained from Gaussian modeling and the three MD force fields for the five peptides. The Gaussian model predicts very dissimilar Ramachandran distributions across all contexts, except for valine residues in GD^P^ **V**G versus GS**V**G peptides, which are associated with moderately dissimilar distributions. All three force fields result in moderately similar to moderately dissimilar Ramachandran distributions, which indicates insufficient changes in the conformational ensemble of valine due to NN effects. In this respect, Amber ff24EXP-GA, with the largest number of moderately dissimilar Hellinger distance values, outperforms the other two force fields.

The comparison of experimental and calculated *J*-coupling constants and reduced values for the valine residue is shown in Figures S9, S10, S11, and S12 (see also Table S6). Figure S9 confirms significant deviations in the two Amber force fields with respect to capturing the enhanced *β*-strand ensemble of the valine residue in V**V**V peptide relative to its intrinsic conformational preferences. Only CHARMM36m shows relatively good agreement with experimental data, as reflected by its rather low $\chi_{J}^{2}$ value compared to $\chi_{J}^{2}$ values obtained with Amber ff14SB and Amber ff24EXP-GA. Valine’s conformational dynamics in GD^P^ **V**G peptide are reproduced best by Amber ff24EXP-GA, whereas CHARMM36m and Amber ff14SB result in much larger $\chi_{J}^{2}$ values (Figure S10-vi). The conformational dynamics of the valine residue in GS**V**G peptide are not captured well by the three force fields (Figures S10 and S11). Only Amber ff24EXP-GA adequately reproduces the comparable pPII and *β*-strand populations observed for the valine residue in GA**V**G peptide, as reflected in the ranking of $\chi_{J}^{2}$ values: Gaussian model < Amber ff24EXP-GA < Amber ff14SB < CHARMM36m (Figure S12-vi). Overall, unlike the two Amber force fields, CHARMM36m at least qualitatively captures the cooperative NN effects of the centralvaline in V**V**V, whereas Amber ff24EXP-GA performs the best among the three force fields in reproducing the NN effects on valine’s conformational ensemble exerted by unlike residues, such as alanine, aspartic acid, and serine.

### Nearest-Neighbor Effects on Serine

The effect of NN interactions on the conformational dynamics of the serine residue is here examined by comparing its conformational ensembles in G**S**AG, G**S**LG, and G**S**VG peptides to its intrinsic conformational ensemble in the G**S**G peptide. Experiment-based Ramachandran distributions and mesostate populations of the serine residue in G**S**G and G**S**yG peptides, shown in Figure 5A and Figure 5B, respectively, demonstrate that NNs modify the intrinsic conformational ensemble in an NN-specific manner.

**Figure 5:**
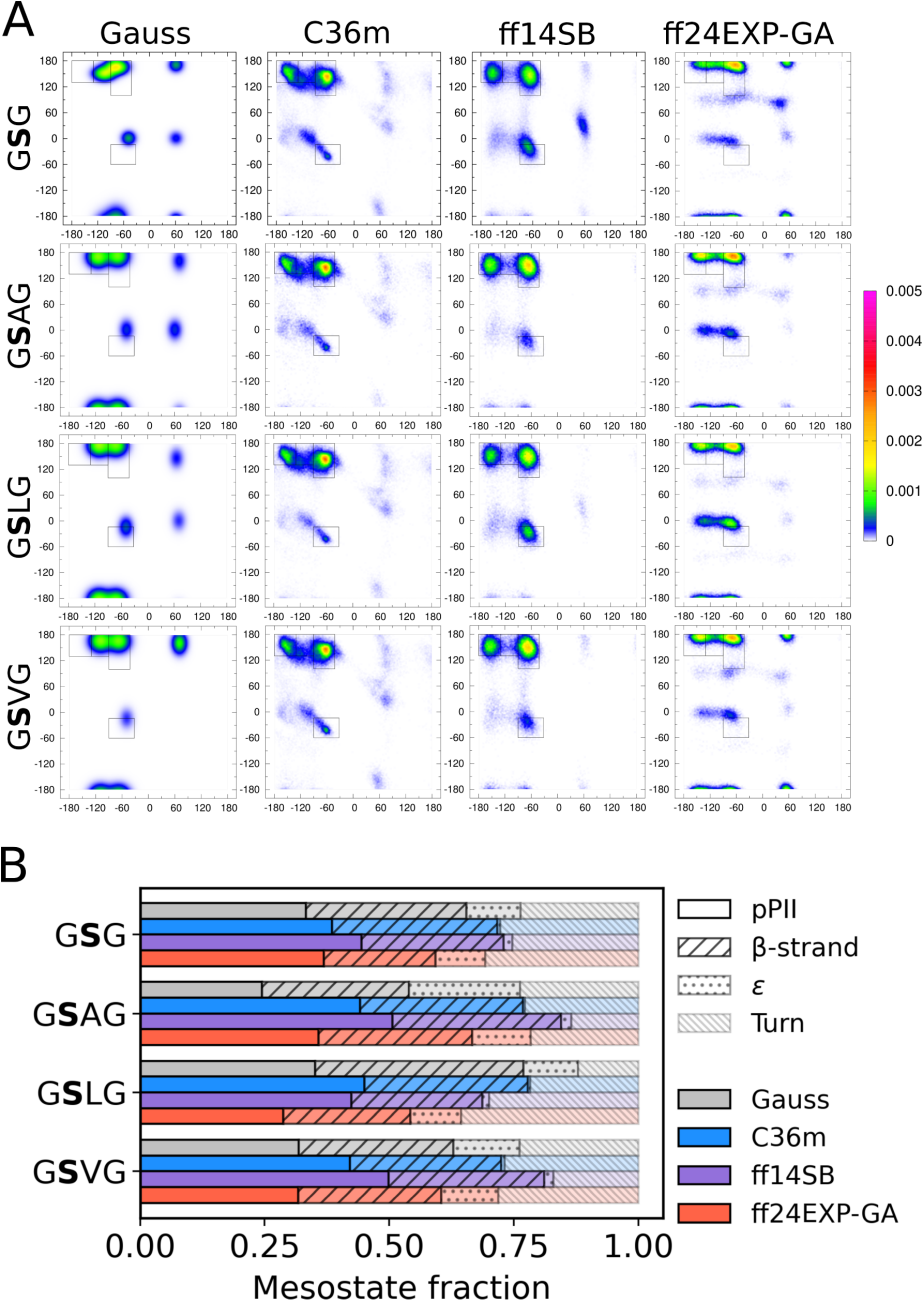
(A) Ramachandran distributions and (B) mesostate populations of serine residue in G**S**G, G**S**AG, G**S**LG, and G**S**VG peptides obtained within the Gaussian model and derived from CHARMM36m, Amber ff14SB, and Amber ff24EXP-GA simulations. Data pertaining to G**S**G Ramachandran distributions for the Gaussian model are reproduced from ref 12 with permission from the Royal Society of Chemistry, and the corresponding MD-derived Ramachandran distributions are taken from Suresh and collaborators (Copyright 2025, American Chemical Society).^21^

Whereas in G**S**G peptide, the serine residue is characterized by pPII, *β*-strand, and a high intrinsic turn-like population,^6^ in the conformational ensemble of the serine residue in G**S**AG peptide, the pPII content is reduced, and the extended conformations in the *ϵ* region (see Figure 1) become more prominent (Figure 5B). The serine residue in G**S**LG peptide shows an increased *β*-strand content and significantly reduced turn content relative to its intrinsic conformational ensemble (Figure 5B). The dominant populations of the serine residue in G**S**AG peptide are similar to those in G**S**G peptide, although the basins are shifted relative to their intrinsic counterparts (Figure 5A-B). We acquire CHARMM36m, Amber ff14SB, and Amber ff24EXP-GA trajectories of GSAG, GSLG, and GSVG peptides and derive the Ramachandran distributions and mesostate populations shown in Figure 5 alongside the Gaussian model results. Visual inspection of Figure 5A suggests that the conformational ensembles of the serine residue are not strongly affected by NNs in the three MD force fields. The four CHARMM36m-derived Ramachandran distributions appear almost identical, whereas there is more variability among the four Ramachandran distributions and mesostate populations derived from the two Amber force fields (Figure 5). The numerical values of mesostate populations are shown in Table S7.

Pairwise Hellinger distances between the Gaussian Ramachandran distributions of the serine residue in G**S**G, G**S**AG, G**S**LG, and G**S**VG peptides in Figure S13A indicate that the serine residue adopts moderately similar conformations in the presence of alanine or leucine residues as NNs. All other Hellinger distances indicate moderately dissimilar Ramachandran distributions (Figure S13A). Both CHARMM36m and Amber ff14SB produce Ramachandran distributions of the serine residue in G**S**G, G**S**AG, G**S**LG, and G**S**VG peptides that are either moderately similar or moderately dissimilar (Figure S13B,C). The moderate similarity between the Ramachandran distributions of the serine residue in G**S**AG and G**S**LG peptides is not captured by any of the three MD force fields. Amber ff24EXP-GA predicts the largest Hellinger distances, indicating predominantly moderately dissimilar pairwise Ramachandran distributions (Figure S13D).

A comparison of calculated and experimental *J*-coupling constants in Figures S14, S15, and S16 (see also numerical values in Table S8) reveals that the Gaussian model outperforms the three MD force fields. Nonetheless, Amber ff24EXP-GA performs significantly better than CHARMM36m and Amber ff14SB, as reflected in the lowest $\chi_{J}^{2}$ values for the serine residue in G**S**AG, G**S**LG, and G**S**VG peptides (Figures S14-vi, S15-vi, and S16-vi).

### Nearest-Neighbor Effects on Aspartic Acid

Experiment-based Gaussian modeling revealed that the intrinsic conformational ensemble of the protonated aspartic acid residue in GD^P^G peptide is dominated by asx turn conformations, which are likely stabilized by the carboxylic acid side chain forming a hydrogen bond with one of the backbone functional groups.^7,8^ Toal and collaborators demonstrated that NNs such as leucine and valine residues in G**D^P^** LG and G**D^P^** VG peptides, respectively, significantly affect the intrinsic propensities of the protonated aspartic acid by diminishing the asx turn content and enhancing both pPII and *β*-strand conformations.^37^ Gaussian Ramachandran distributions and mesostate populations demonstrate that the leucine residue as an NN affects the conformational ensemble of the aspartic acid residue more than the valine residue (Figure 6). We performed MD simulations of GD^P^LG and GD^P^VG peptides in the three MD force fields, derived the resulting Ramachandran distributions of the protonated aspartic acid residue in G**D^P^** LG and G**D^P^** VG peptides, and compared the MD-derived Ramachandran distributions and mesostate populations to their intrinsic conformational ensemble of the protonated aspartic acid residue in GD^P^G peptide. MD-derived Ramachandran distributions are shown in Figure 6A and the corresponding mesostate populations are displayed in Figure 6B (see also Table S9), alongside the Gaussian model counterparts.

**Figure 6:**
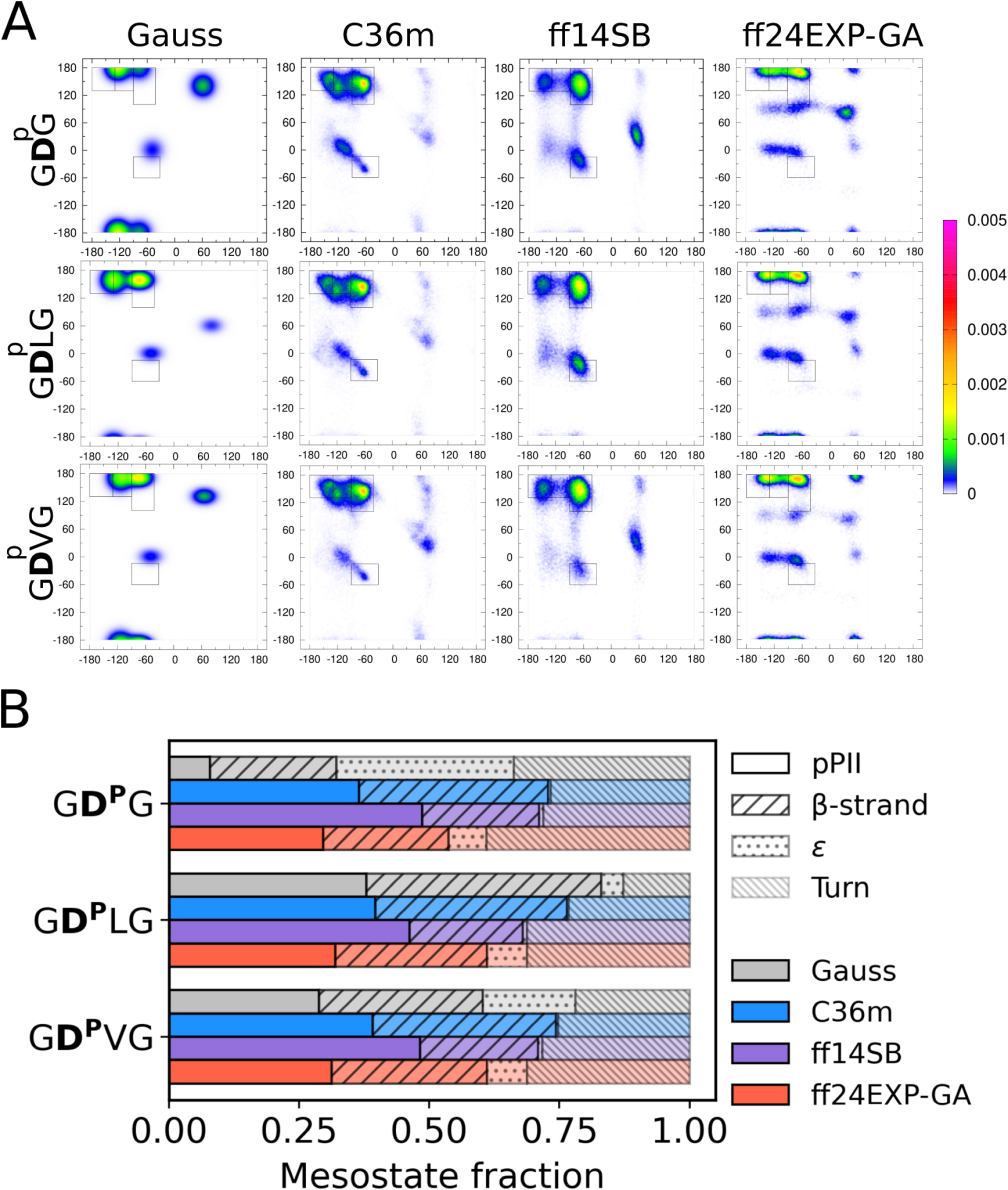
(A) Ramachandran distributions and (B) mesostate populations of protonated aspartic acid (D^P^) residue in GD^P^G, GD^P^LG, and GD^P^VG peptides obtained within the Gaussian model and derived from CHARMM36m, Amber ff14SB, and Amber ff24EXP-GA simulations. Data pertaining to G**D^P^** G Ramachandran distributions for the Gaussian model are reproduced from ref.^12^ with permission from the Royal Society of Chemistry, and the corresponding MD-derived Ramachandran distributions are taken from Suresh and collaborators (Copyright 2025, American Chemical Society).^21^

The three MD force fields capture the NN effects of aliphatic residues on the aspartic acid residue with varying degrees of accuracy. Of the three MD force fields, only Amber ff24EXP-GA reproduces the experimental observation of reduced turn populations of the aspartic acid residue in both G**D^P^** LG and G**D^P^** VG peptides (Figure 6B and Table S9).

A comparison of Hellinger distances in Figure S17 reveals that the Gaussian model predicts overall larger distances among the Ramachandran distributions of the aspartic acid residue in G**D** ^P^G, G**D** ^P^LG, and G**D** ^P^VG peptides. The Gaussian model shows that the leucine residue indeed exerts a stronger effect on the Ramachandran distribution of the aspartic acid residue than the valine residue. Figure S17 demonstrates that CHARMM36m underestimates the effect of NN on the aspartic acid residue more than the two Amber force fields.

A direct comparison of Gaussian and MD-derived *J*-coupling constants to their respective experimental values for the aspartic acid residue in G**D^P^** LG and G**D^P^** VG peptides is shown in Figures S18 and S19 (see also Table S10). Of the three MD force fields, Amber ff24EXP-GA is associated with the lowest $\chi_{J}^{2}$ values for the aspartic acid residue in these two peptides (Figures S18-vi and S19-vi).

To delve into the effects of NNs on the conformational ensemble of aspartic acid residue, we additionally performed MD simulations of cationic GD^P^D^P^G and GD^P^D^P^D^P^G peptides within all three force fields (see *Methods*) and examined the conformational dynamics of all aspartic acid residues in GD^P^D^P^G, and GD^P^D^P^D^P^G, for which experimental *J*-coupling constants have been reported previously.^37,40,64^ The experimental data and Gaussian model analysis reported by Milorey and collaborators revealed an enhancement of different turn mesostates for several aspartic acid residues in GD^P^D^P^G and GD^P^D^P^D^P^G peptides relative to the intrinsic conformational ensemble of the aspartic acid residue in G**D** ^**P**^G peptide.^40^ The Gaussian- and MD-derived Ramachandran distributions and mesostate populations of aspartic acid residues in GD^P^G, GD^P^D^P^G, and GD^P^D^P^D^P^G peptides are displayed in Figure 7 (see also Table S11).

**Figure 7:**
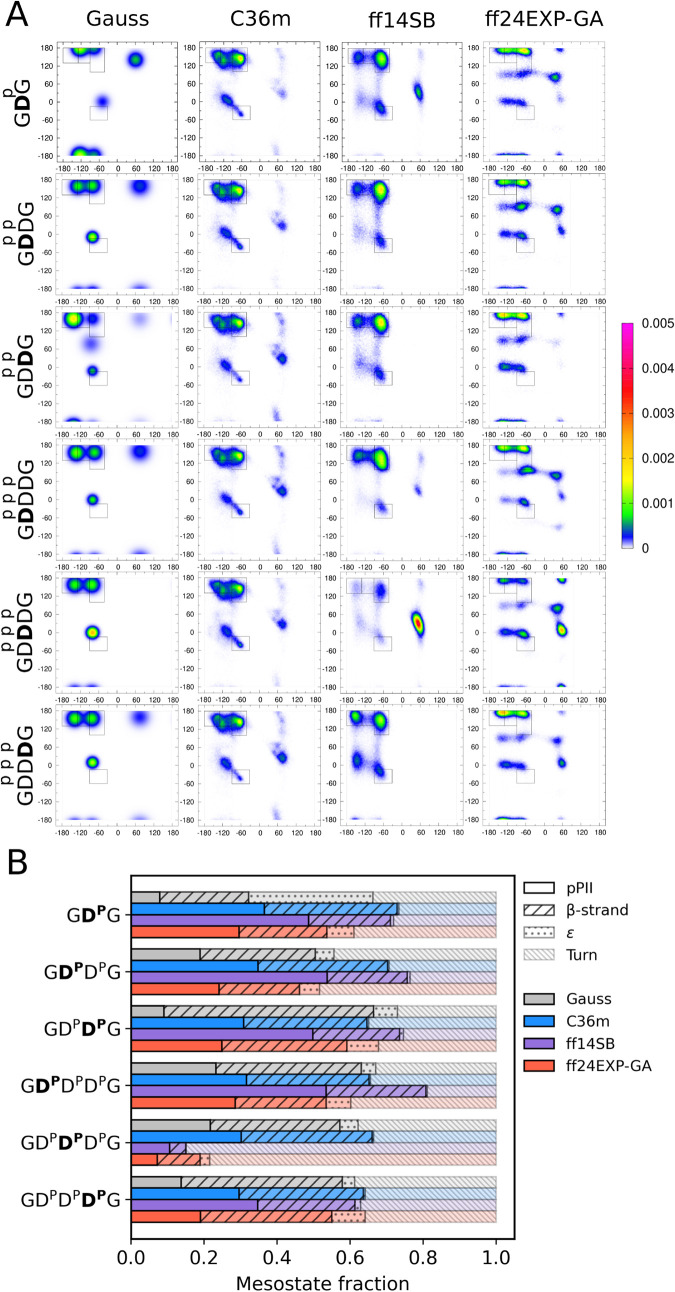
(A) Ramachandran distributions and (B) mesostate populations of protonated aspartic acid residue in GD^P^G, GD^P^D^P^G, and GD^P^D^P^D^P^G peptides obtained within the Gaussian model and derived from CHARMM36m, Amber ff14SB, and Amber ff24EXP-GA simulations. The Gaussian Ramachandran distributions were generated based on parameters reported by Milorey and collaborators (Copyright 2021, American Chemical Society).^40^

Gaussian modeling reveals that, relative to the intrinsic conformational ensemble of the aspartic acid residue, the first aspartic acid residue in GD^P^D^P^G peptide exhibits a markedly increased I/II’ *β*_*i*+2_ turn and reduced *β*-strand content. In contrast, the second aspartic acid residue in GD^P^D^P^G peptide displays predominant *β*-strand conformations, with notably lower turn and pPII populations relative to both the first aspartic acid residue in the same peptide and the aspartic acid residue in G**D** ^**P**^G peptide.^40^ The CHARMM36m-derived Ramachandran distributions do not show large context dependence, resulting in rather similar Ramachandran distributions for all aspartic acid residues in GD^P^G and GD^P^D^P^G peptides, whereas both Amber force fields exhibit a bit more context dependence. Hellinger distances among Gaussian Ramachandran distributions of aspartic acid residues in these two peptides are all very dissimilar, whereas CHARMM36m predicts very similar distributions, underestimating the context dependence more than the two Amber force fields (Figure S20), which exhibit moderately to very dissimilar distributions. A comparison of calculated and experimental *J-*coupling constants in Figures. S21 and S22 (see also Table S12) shows that, of the three MD force fields, CHARMM36m and Amber ff24EXP-GA result in the lowest $\chi_{J}^{2}$ values for the first and second aspartic acid residues in GD^P^D^P^G peptides, respectively (Figures. S21-vi and S22-vi).

To obtain further insights into the neighboring effects of like residues on the conformational ensemble of the aspartic acid residue, we acquired MD simulations of the cationic GD^P^D^P^D^P^G peptide in three MD force fields (see *Methods*) and analyzed the conformational ensembles of all three aspartic acid residues. This is the longest peptide examined in this work. Experiment-based Gaussian Ramachandran distributions and MD-derived Ramachandran distributions are shown in Figure 7A and the respective mesostate populations are shown in Figure 7B (see also Table S11). The experiment-based Gaussian Ramachandran distribution of the first aspartic acid residue in GD^P^D^P^D^P^G peptide is similar to that of the aspartic acid residue in G**D** ^**P**^G peptide (Figure 7A). For the central aspartic acid residue in GD^P^D^P^D^P^G peptide, the Gaussian Ramachandran distribution and mesostate populations indicate balanced pPII, *β*-strand, and I/II’ *β*_*i*+2_-turn conformations, with no detectable asx-turn-like conformations. The conformational ensemble of the third aspartic acid residue in GD^P^D^P^D^P^G peptide exhibits reduced asx-turn-like conformations and a modest enrichment of pPII and *β*-strand conformations (Figure 7). Gaussian Ramachandran distributions for these three aspartic acid residues are very dissimilar from the intrinsic Ramachandran distribution, as assessed by the Hellinger distance, but are moderately similar or dissimilar to each other (Figure S23A). CHARMM36m results in very similar Ramachandran distributions for all three aspartic acid residues, which is also confirmed using the Hellinger distance comparisons (Figure S23B). Both Amber ff14SB and Amber ff24EXP-GA predict moderately to very dissimilar Ramachandran distributions for the three aspartic acid residues (Figure. S23C and D).

A comparison of calculated and experimental *J*-coupling constants of the three aspartic acid residues in GD^P^D^P^D^P^G peptide in Figures. S24, S25, and S26 (see also Table S13) shows that CHARMM36m outperforms both Amber force fields and results in the lowest $\chi_{J}^{2}$ values (Figures. S24-vi, S25-vi, and S26-vi). For all three aspartic acid residues, the $\chi_{J}^{2}$ values from the lowest to the highest are obtained within CHARMM36m as the most accurate, followed by Amber ff24EXP-GA, and Amber ff14SB, which predicts the least accurate results.

Both Amber force fields produce highly inaccurate results for the central aspartic acid residue in GD^P^D^P^D^P^G peptide, which is reflected in the respective Ramachandran distributions in Figure 7A as enhanced turn populations, mostly in the right-hand side of the Ramachandran space (*ϕ* > 0). This anomalous behavior strongly deviates from the experimental predictions. To investigate the cause of the peculiar conformational ensemble of the central aspartic acid residue in GD^P^D^P^D^P^G peptide in the two Amber force fields, we performed a cluster analysis of GD^P^D^P^D^P^G conformational ensembles within all three MD force fields. Figure. S27 shows the top five clusters of GD^P^D^P^D^P^G conformations in a ranking order from the most (1) to the least (5) populated, for each of the three force fields. Whereas the two most populated clusters in CHARMM36m, which encompass about 89% of all conformations, correspond to extended conformations, in both Amber force fields, the most populated cluster of GD^P^D^P^D^P^G conformations, containing 78% (Amber ff14SB) and 66% (Amber ff24EXP-GA) of all conformations, is associated with a turn-like structure. Importantly, the average dihedral angles of the central aspartic acid in GD^P^D^P^D^P^G peptide associated with each of the five predominant clusters in Figure. S28 reveal that the most populated clusters in Amber force fields constrain the conformational ensemble of the central aspartic acid to turn regions, I’/III’ *β*_*i*+1_ and I’/II *β*_*i*+2_ in Amber ff14SB and Amber ff24EXP-GA, respectively. A visual inspection of conformations belonging to cluster 1 in the two Amber force fields suggests a strong interaction between the protonated amino group of the N-terminus and the carboxylic acid side chain group of the third aspartic acid residue, which restricts the conformational ensemble of the central aspartic acid residue between these two strongly interacting moieties to the turn regions in the *ϕ* > 0 half. To test this observation, we calculated the histogram of distances between the N-terminal protonated amino group and the carboxylic acid side chain group of the third aspartic acid residue along the trajectory for each of the three force fields. Results in Figure. S29 reveal three peaks in the Amber ff14SB-derived histogram of distances, dominated by distances at ∼0.2 nm. In contrast, the CHARMM36m-derived histogram of distances is strongly dominated by distances centered at ∼ 0.9 nm, and there is no peak at ∼0.2 nm. The histogram of distances derived from Amber ff24EXP-GA shows a bimodal distribution with distances centered at ∼0.9 nm and ∼0.2 nm. These observations combined demonstrate that, unlike CHARMM36m, the two Amber force fields predict strongly (Amber ff14SB) or partially (Amber ff24EXP-GA) folded cationic GD^P^D^P^D^P^G peptide. Consequently, the Amber-derived conformational ensembles of the central aspartic acid residue in GD^P^D^P^D^P^G peptide are strongly affected by tertiary contacts, which lead to large deviations from experimental data.

## Conclusions

MD is an important tool for studying IDPs, complementing experimental approaches by elucidating processes at an atomistic level of detail that occur at short time scales, which are often inaccessible to experiments. The reliability of MD predictions, however, depends on the accuracy of the underlying MD force field. Systematic benchmarking of MD force fields provides critical insights into their strengths and limitations, informing optimal simulation strategies and guiding force field development. Recent studies revealed that commonly used MD force fields do not capture the intrinsic conformational dynamics of guest residues x in short GxG peptides in water, in agreement with experimental data.^10–13^ Using Amber ff14SB as a parent force field, Suresh and collaborators used spectroscopic data on guest glycine and alanine in GGG and GAG peptides to develop Amber ff24EXP-GA, which reproduces the experimental data on 14 guest residues x in GxG peptides significantly better than Amber ff14SB and exhibits more residue specificity than Amber ff14SB and CHARMM36m.^21^ We here ask to what extent three MD force fields (CHARMM36m, Amber ff14SB, and Amber ff24EXP-GA) capture the NN effects on the conformational dynamics of a residue of interest in selected tripeptides and tetrapeptides in water. The short peptides have been selected based on the availability of experimental data, which comprise five *J*-coupling constants.^36,37,39^ These experimental data are used to derive Gaussian Ramachandran distributions that best fit the experimental data and can thus be used as benchmarks in the assessment of MD force fields.^9,61^ The results of the MD force-field assessment performed in this work are summarized in Figure 8, which compares the reduced $\chi_{J}^{2}$ values of 16 amino acid residues in tri- and tetrapeptides derived from the three MD force fields alongside the benchmark Gaussian model predictions. The average per-residue $\chi_{J}^{2}$ values obtained in the Gaussian model and derived from the three MD force fields, with the respective SEM values in Table 1 demonstrate that all MD force fields perform significantly worse than the benchmark Gaussian model. Amber ff24EXP-GA outperforms the parent force field Amber ff14SB and performs comparably to CHARMM36m.

**Figure 8:**
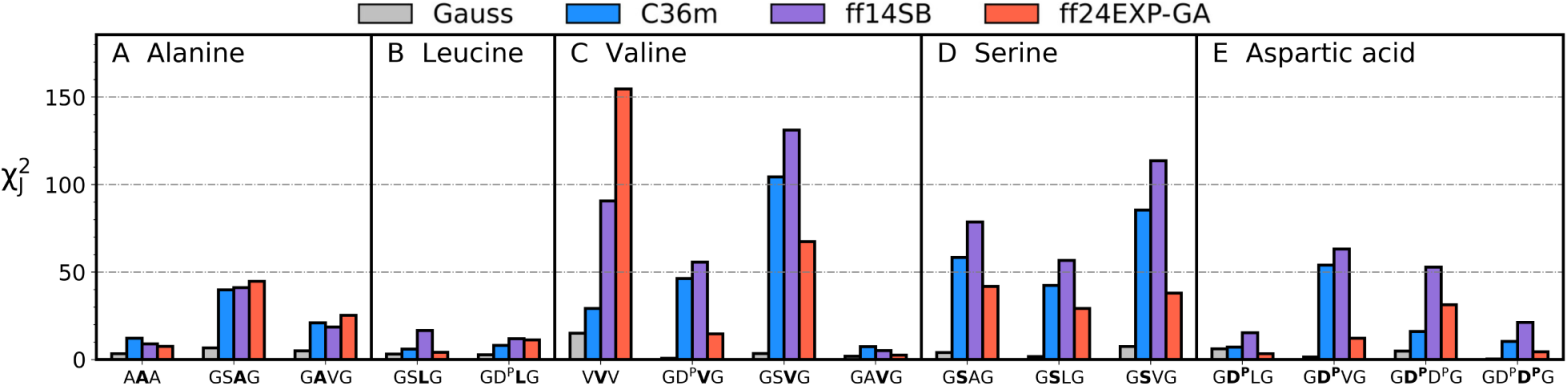
Assessment of the Gaussian model and MD force fields with respect to their ability to reproduce experimental NN effects for (A) alanine, (B) leucine, (C) valine, (D) serine, and (E) protonated aspartic acid residues in short unfolded tri- and tetrapeptides.

**Table 1:** The Average (⟨.⟩) and Standard Error of Mean *σ*_SEM_ of $\chi_{J}^{2}$ Values of All Residues Assessed per Model/Force Field

Model	$\langle \chi_{J}^{2}\rangle \pm \sigma_{\mathrm{SEM}}$
Gaussian	4.04 ± 3.34
CHARMM36m	32.54 ± 26.43
Amber ff14SB	46.60 ± 34.72
Amber ff24EXP-GA	31.02 ± 36.76

Our results demonstrate that the three force fields exhibit highly context-dependent performance with respect to reproducing experimental $\chi_{J}^{2}$ values, mesostate populations, and Hellinger distances between Ramachandran distributions of amino acid residues across different NN environments. Whereas none of the three force fields exhibits superior performance in describing the influence of like and unlike NN residues on the conformational dynamics of the residue of interest in short unfolded peptides, Amber ff24EXP-GA emerges as the most accurate force field, achieving the lowest $\chi_{J}^{2}$ for 11 out of 14 contexts shown in Figure 8. Amber ff24EXP-GA accurately models the serine residue in all serine-containing peptides in this study, the valine residue with unlike neighbors, and the aspartic acid residue neighbored by unlike residues. Amber ff24EXP-GA exhibits the lowest accuracy for the alanine residue when it is neighbored by unlike residues in G**A**VG and GS**A**G peptides but accurately captures the pPII enhancement and hence has the lowest $\chi_{J}^{2}$ value for the central alanine in A**A**A peptide. Amber ff24EXP-GA outperforms its parent force field, Amber ff14SB, for all residues examined here except for the central valine residue in the VVV peptide. For the central valine residue in the VVV peptide, Amber ff24EXP-GA strongly deviates from experimental constraints and performs the worst of the three force fields. Although Amber ff24EXP-GA performs better than the other two force fields for residues shown in Figure 8, the ability of CHARMM36m to capture the NN experimental data is comparable to that of Amber ff24EXP-GA (Table 1). Furthermore, CHARMM36m outperforms the two Amber force fields in capturing the conformational dynamics of all protonated aspartic acid residues in GD^P^ **D^P^** D^P^G peptide. Cluster and distance analyses of GD^P^ **D^P^** D^P^G peptide in the three MD force fields revealed that, in the two Amber force fields, this peptide forms a tertiary contact between the protonated N-terminal amino group and the carboxylic acid side group of the third aspartic acid residue, which sterically constrains the central aspartic acid residue to turn conformations in the *ϕ* > 0 region of the Ramachandran space. This means that, in Amber ff24EXP-GA and even more so in Amber ff14SB, GD^P^ **D^P^** D^P^G peptide is not unfolded, which is at odds with experimental data. For this reason, this peptide is not included in the summary of results of this study in Figure 8 and Table 1. The deviations of the conformational ensemble of the central aspartic acid in GD^P^ **D^P^** D^P^G peptide in the two Amber force fields from experimental predictions also illustrate the extent to which tertiary structure may affect the conformational dynamics of the residue of interest.

To summarize, we here demonstrate that Amber ff24EXP-GA, which has been optimized using experiment-based Gaussian Ramachandran distributions of glycine and alanine residues in GGG and GAG peptides, respectively, results in amino acid residue-specific Ramachandran distributions of 14 guest residues x in GxG peptides that significantly better reproduce the experimental data than the parent Amber ff14SB.^21^ It also captures the effects of NNs on various residues of interest in unfolded tri- and tetrapeptides better than Amber ff14SB. This work thus shows that the improvements in shorter unfolded peptides are transferable to longer unfolded peptides, further validating Amber ff24EXP-GA. Although CHARMM36m lacks the residue-specific accuracy that Amber ff24EXP-GA often achieves, it maintains a strong average agreement with experiment data across the tested residues and peptides. This likely reflects the distinct parameterization of CHARMM36m, whereby the coupled *ϕ*–*ψ* correction-map potential is fit using larger IDPs with an emphasis on global structural features, such as overall helical content, left-handed helix populations, and chain dimensions, resulting in lower residue specificity yet overall good average accuracy with respect to $\chi_{J}^{2}$ values of various amino acid residues in unfolded peptides. Amber ff24EXP-GA’s focus on residue-specific intrinsic propensities versus CHARMM36m’s emphasis on global structural properties and secondary structure propensities highlights a fundamental trade-off in force-field development and improvement. Neither approach fully captures the interplay between the intrinsic preferences of amino acid residues in water and their modifications due to NN effects. Next-generation parameterization will need to optimize both local residue-specific accuracy and transferability to diverse sequence contexts, as well as global properties of longer unfolded peptides and IDPs.

## Data and Software Availability

Data and Software Availability: All analysis scripts used for calculating *J*-coupling constants, Hellinger distances, and mesostate populations, alongside all the Gaussian and MD-derived Ramachandran distributions used in this study, are provided in Supporting Information.

## Supplementary Material







## Notes

Data and Analysis Codes (ZIP)
Gaussian Modeling and J-coupling constants (ZIP)
Tables (S1–S13) with the mesostate populations, absolute differences between calculated and experimental *J*-coupling constants, and $\chi_{J}^{2}$ values for amino acid residues in different NN contexts within the Gaussian model and the three MD force fields; figures (S1–26) with Hellinger distance comparisons of a residue of interest in various contexts, absolute differences between calculated and experimental values of the five *J*-coupling constants, and reduced values for each residue of interest within the Gaussian model and the three MD force fields; figures (S27–S29) with the predominant five clusters of GD^P^D^P^D^P^G conformations derived from CHARMM36m, Amber ff14SB, and Amber ff24EXP-GA simulations; the average dihedral angles for the central aspartic acid residue in GD^P^D^P^D^P^G peptide for the five predominant clusters of conformations; time evolution and histograms of the minimum distance, *d*_min_, between ${\mathrm{NH}}_{3}^{+}$ group of GLY1 and the side chain COOH group of ASPH4 in GDPDPDPG peptide derived from CHARMM36m, Amber ff14SB, and Amber ff24EXP-GA simulations (PDF)
