# Supplementary material for: Nearest-Neighbor Effects in Short Unfolded Peptides: An Assessment of Molecular Dynamics Force Fields
Source: J Chem Inf Model. 2026 Jun 4;66(12):7190–206. doi: 10.1021/acs.jcim.6c00438 (PMC13292223; doi:10.1021/acs.jcim.6c00438)
Supplement: Supplementary file 2 [file ci6c00438_si_002.zip › Gaussian_Modeling_Jcoupling/REDME_Matlab_J_coupling.docx]

**Workflow for the calculation of Ramachandran plots and scalar J-coupling constants for GxG and GxyG peptides.**

**General description**

The program uses the mole fractions, φ and ψ positions and the respective halfwidths of Gaussian sub-distributions as input parameters. The respective parameter values for GxG peptides were taken from previous papers.^1–5^ All off-diagonal correlation parameters in the two-dimensional Gaussian formalism were set to zero, so that all basins appear elliptical with their axis along φ and ψ in the Ramachandran plot. Each Gaussian sub-distribution is associated with a secondary structure. The Ramachandran plots are calculated as the superposition of the Gaussian sub-distributions and used as probability density function to calculate the ensemble average of J-coupling constants by use of Karplus equations. The underlying mathematics is described in ref.^6^

**Organization of the program**

After defining some parameters that allow the switch between different sets of Karplus equations and the number of peptides for which calculations should be carried out, the input parameters are all listed. They are organized as follows:

1. The mole fractions chi(n,m), n: number of secondary structures, m: number of peptides,
2. The phimax(n,m) and psimax(n,m) coordinates of the Gaussian sub-distribution positions in deg.
3. The wpi(n,m) and wpsi(n,m) halfwidths in deg
4. The off-diagonal correlation parameter cov(n,m)
5. The experimental J-coupling constants J1(m),…,J5(m) and the respective experimental uncertainties.^7^

The number is related to secondary structure as follows: n=1: pPII; n=2: β-strand, n=3: right handed helical; n=4: left-handed helical; n=5: can vary, options are inverse γ- or asx-turns (just check the dihedral angles), n=6-9 accounts for the boundary overlap of n=1,2, and 5. The J-coupling parameters are defined as follows: J1: ^3^J(H^N^H^Cα^), J2: ^3^J(H^N^C’), J3: ^3^J(H^Cα^C’), J4: ^3^J(H^N^C_β_), and J5: ^1^J(NC_α_) (lines 23-2374)

The peptides are listed at the respective tops of the parameter list. The tetrapeptides are listed with respect to the central residue of the tripeptide. The list starts with GAG (m=1), followed by the parameters of alanine in GsAG, GdAG, GAvG and GAlG (lower case indicates neighbors of alanine, the one letter code of amino acids is used). The remainder of the parameter list is now self-explanatory. The number of peptides can be limited by the k1max number at the beginning of the program. K1max=33 means that Gaussian Ramachandran plots and J-coupling constants of all 33 peptides considered will be calculated.

The calculation of Ramachandran plots and J-coupling constants starts on line 2375. A loop associated with k1=1,2,3,…,kmax calculates them for all to be considered peptides. J-coupling parameters, computational errors and probability densities are calculated for φ- and ψ-values in increments of 2 deg for the entire Ramachandran space. Once the entire space has been sampled, the ensemble average of J-coupling and their statistical errors (experimental and computational combined by Gaussian error propagation) are calculated (2590-2599). From 2600 to the end, individual coupling constants, their uncertainties are read out for each peptide number by means of if/elseif commands. The reduced chi-square functions for each peptide are calculated. The respective Ramachandran probability density functions are read out and plotted.

**References**

(1) Hagarman, A.; Measey, T. J.; Mathieu, D.; Schwalbe, H.; Schweitzer-Stenner, R. Intrinsic Propensities of Amino Acid Residues in GxG Peptides Inferred from Amide I’ Band Profiles and NMR Scalar Coupling Constants. *J. Am. Chem. Soc.* 2010, *132* (2), 540–551. https://doi.org/10.1021/ja9058052.

(2) Hagarman, A.; Mathieu, D.; Toal, S.; Measey, T. J.; Schwalbe, H.; Schweitzer-Stenner, R. Amino Acids with Hydrogen-Bonding Side Chains Have an Intrinsic Tendency to Sample Various Turn Conformations in Aqueous Solution. *Chemistry - A European Journal* 2011, *17* (24), 6789–6797. https://doi.org/10.1002/chem.201100016.

(3) Schweitzer-Stenner, R.; Hagarman, A.; Toal, S.; Mathieu, D.; Schwalbe, H. Disorder and Order in Unfolded and Disordered Peptides and Proteins: A View Derived from Tripeptide Conformational Analysis. I. Tripeptides with Long and Predominantly Hydrophobic Side Chains. *Proteins: Structure, Function and Bioinformatics* 2013, *81* (6), 955–967. https://doi.org/10.1002/prot.24225.

(4) Milorey, B.; Schwalbe, H.; O’Neill, N.; Schweitzer-Stenner, R. Repeating Aspartic Acid Residues Prefer Turn-like Conformations in the Unfolded State: Implications for Early Protein Folding. *J. Phys. Chem. B* 2021, *125* (41), 11392–11407. https://doi.org/10.1021/acs.jpcb.1c06472.

(5) Schweitzer-Stenner, R. Nearest Neighbour Interactions between Amino Acid Residues in Short Peptides and Coil Libraries. January 22, 2026. https://doi.org/10.64898/2026.01.19.700493.

(6) Schweitzer-Stenner, R. Distribution of Conformations Sampled by the Central Amino Acid Residue in Tripeptides Inferred from Amide i Band Profiles and NMR Scalar Coupling Constants. *J. Phys. Chem. B* 2009, *113* (9), 2922–2932. https://doi.org/10.1021/jp8087644.

(7) Toal, S. E.; Kubatova, N.; Richter, C.; Linhard, V.; Schwalbe, H.; Schweitzer‐Stenner, R. Randomizing the Unfolded State of Peptides (and Proteins) by Nearest Neighbor Interactions between Unlike Residues. *Chemistry - A European Journal* 2015, *21*, 5173–5192.
