# Supplementary material for: Nearest-Neighbor Effects in Short Unfolded Peptides: An Assessment of Molecular Dynamics Force Fields
Source: J Chem Inf Model. 2026 Jun 4;66(12):7190–206. doi: 10.1021/acs.jcim.6c00438 (PMC13292223; doi:10.1021/acs.jcim.6c00438)
Supplement: Supplementary file 3 [file ci6c00438_si_003.pdf]

Supporting Information: Nearest-Neighbor Effects in Short Unfolded Peptides: An Assessment of Molecular Dynamics Force Fields†

Athul Suresh,† Reinhard Schweitzer-Stenner,‡ and Brigita Urbanc*,†

†*Department of Physics, Drexel University, Philadelphia, PA 19104, USA*

‡*Department of Chemistry, Drexel University, Philadelphia, PA 19104, USA*

E-mail: bu25@drexel.edu

Supporting Tables

Table S1: Mesostate populations (%) for alanine residues in cationic AAA, GAG, GSAG, and GAVG peptides obtained from the Gaussian model and the three MD force fields. The Gaussian Ramachandran distributions of alanine residues in GAG and AAA are taken from Zhang et al.[S2] (Copyright 2020 American Chemical Society), whereas the Gaussian Ramachandran distributions of alanine residues in GSAG, and GAVG are reproduced from Schweitzer-Stenner[S6] with permission from the Royal Society of Chemistry. MD-derived Ramachandran distribution for alanine residue in GAG is taken from Suresh et al.[S4]

**Peptide**	**Mesostate**	**Gaussian**	**C36m**	**ff14SB**	**ff24EXP-GA**
	pPII	77.6	58.8	73.9	73.1
A**A**A	β-strand	13.4	23.3	17.6	16.7
	ε	0.0	1.0	3.4	4.0
	turn	9.0	16.9	5.1	6.2
	pPII	61.1	56.1	49.9	63.3
G**A**G	β-strand	20.2	23.4	19.4	20.0
	ε	8.00	0.9	2.4	4.0
	turn	10.8	19.6	28.3	12.7
	pPII	39.0	51.6	54.8	58.7
GS**A**G	β-strand	29.6	23.2	15.6	21.3
	ε	2.4	0.9	2.5	3.7
	turn	29.0	24.3	27.1	16.3
	pPII	39.4	57.0	63.4	66.5
G**A**VG	β-strand	32.1	23.5	19.5	18.8
	ε	7.5	0.7	2.7	3.4
	turn	21.0	18.7	14.4	11.3

Table S2: Absolute errors between calculated and experimental J-coupling constants and χ_J^2 values for alanine residue in AAA, GSAG, and GAVG peptides obtained from the Gaussian model and the three MD force fields. The Gaussian Ramachandran distribution of alanine residue in AAA is taken from Zhang et al.[S2] (Copyright 2020 American Chemical Society), whereas the Gaussian Ramachandran distributions of alanine residues in GSAG and GAVG peptides are reproduced from Schweitzer-Stenner[S6] with permission from the Royal Society of Chemistry.

**Peptide**	**Model**	$^3J(H^N, H^{C_\alpha})$ (Hz)	$^3J(H^N, H^{C'})$ (Hz)	$^3J(H^{C_\alpha}, C')$ (Hz)	$^3J(N, H^{C_\beta})$ (Hz)	$^1J(N, C^\alpha)$ (Hz)	χ_J^2
A**A**A	Gaussian	0.002	0.143	0.298	0.244	0.081	3.315
	C36m	0.401	0.145	0.007	0.616	0.010	12.120
	ff14SB	0.178	0.016	0.249	0.435	0.291	8.947
	ff24EXP-GA	0.259	0.206	0.262	0.374	0.183	7.631
GS**A**G	Gaussian	0.314	0.171	0.503	0.280	0.194	6.612
	C36m	0.102	0.358	1.335	0.352	0.137	39.886
	ff14SB	0.148	0.234	1.443	0.157	0.104	41.103
	ff24EXP-GA	0.306	0.397	1.384	0.199	0.061	44.639
G**A**VG	Gaussian	0.166	0.094	0.148	0.700	0.228	4.986
	C36m	0.474	0.004	0.701	0.639	0.203	20.964
	ff14SB	0.532	0.098	0.750	0.548	0.002	18.585
	ff24EXP-GA	0.854	0.039	0.840	0.451	0.074	25.254

Table S3: Mesostate populations (%) for leucine residue in GLG, GSLG, and GD^{P}LG peptides obtained from the Gaussian model and the three MD force fields. The Gaussian model and MD-derived Ramachandran distributions for leucine in GLG are taken from Schweitzer-Stenner(Copyright 2009 American Chemical Society)[S1] and Suresh et al. (Copyright 2025 American Chemical Society),[S4] respectively. The Gaussian Ramachandran distributions for GSLG and GD^{P}LG used in this analysis are reproduced from Schweitzer-Stenner[S6] with permission from the Royal Society of Chemistry.

Peptide	Mesostate	Gaussian	C36m	ff14SB	ff24EXP-GA
G**L**G	pPII	44.3	52.2	63.6	49.7
	β-strand	23.1	24.5	17.5	30.3
	ε	13.6	0.4	1.4	2.3
	turn	19.0	22.9	17.5	17.7
GS**L**G	pPII	22.0	42.4	43.5	34.1
	β-strand	25.7	21.5	20.6	39.9
	ε	30.3	0.02	1.4	2.6
	turn	22.0	35.9	34.5	23.4
GD^{P}**L**G	pPII	47.1	50.2	47.2	36.9
	β-strand	38.0	26.1	21.2	34.2
	ε	5.0	0.3	1.3	2.5
	turn	10.9	23.4	30.3	26.4

Table S4: Absolute errors (Hz) between calculated and experimental J-coupling constants for leucine residue L in GSLG and GD^{P}LG in the Gaussian model and the three MD force fields. χ^2_{J} denotes the reduced chi-squared value. The Gaussian Ramachandran distributions for GSLG and GD^{P}LG **used in this analysis are reproduced from Schweitzer-Stenner[S6] with permission from the Royal Society of Chemistry.**

**Peptide**	**Model**	$^3\mathrm{J}(\mathrm{H^N}, \mathrm{H^{C_\alpha}})$ (Hz)	$^3\mathrm{J}(\mathrm{H^N}, \mathrm{H^{C'}})$ (Hz)	$^3\mathrm{J}(\mathrm{H^{C_\alpha}}, \mathrm{C'})$ (Hz)	$^3\mathrm{J}(\mathrm{N}, \mathrm{H^{C_\beta}})$ (Hz)	$^1\mathrm{J}(\mathrm{N}, \mathrm{C^\alpha})$ (Hz)	χ^2_{J}
GS**L**G	Gaussian	0.075	0.284	0.338	0.089	0.543	3.170
	C36m	0.339	0.194	0.298	0.076	0.276	5.983
	ff14SB	0.450	0.119	0.717	0.163	0.169	16.644
	ff24EXP-GA	0.069	0.380	0.184	0.235	0.001	4.088
GD^{P}**L**G	Gaussian	0.063	0.178	0.093	0.014	0.940	2.805
	C36m	0.011	0.055	0.430	0.071	0.415	8.124
	ff14SB	0.138	0.064	0.570	0.092	0.416	11.869
	ff24EXP-GA	0.342	0.363	0.165	0.273	0.545	11.174

Table S5: Mesostate populations (%) for valine residues V in VVV (central), GVG, GD^{P}VG, GSVG, and GAVG peptides in the Gaussian model and the three MD force fields. The Gaussian Ramachandran distribution for cental valine residue in VVV is taken from Schweitzer-Stenner,[S1] whereas the Gaussian Ramachandran distributions of valine residues in GD^{P}VG, GSVG, and GAVG used in this analysis are reproduced from[S6] with permission from the Royal Society of Chemistry. The Gaussian Ramachandran distribution of valine residue in GVG peptide is reproduced from Andrews et al.[S3] with permission from the Royal Society of Chemistry, whereas the corresponding MD-derived Ramachandran distributions are taken from Suresh et al. (Copyright 2025 American Chemical Society).[S4]

Peptide	Mesostate	Gaussian	C36m	ff14SB	ff24EXP-GA
	pPII	11.5	62.4	75.5	50.7
V**V**V	β-strand	54.7	32.2	17.6	34.9
	ε	1.9	0.0	0.2	7.3
	turn	31.9	5.4	6.7	7.1
	pPII	30.0	56.3	52.4	41.1
G**V**G	β-strand	41.8	24.8	22.2	31.7
	ε	6.2	0.2	0.4	7.0
	turn	22.0	18.7	25.0	20.2
	pPII	13.7	50.9	48.2	36.4
GD^P**V**G	β-strand	64.2	28.3	20.8	40.7
	ε	1.3	0.1	0.3	7.9
	turn	20.8	20.7	30.7	15.0
	pPII	16.2	54.5	43.9	29.9
GS**V**G	β-strand	54.7	30.6	24.7	34.9
	ε	1.6	0.1	0.2	6.1
	turn	27.5	14.8	31.2	29.1
	pPII	16.2	51.2	55.4	32.5
GA**V**G	β-strand	47.4	27.6	22.8	36.2
	ε	0.7	0.0	0.3	6.9
	turn	16.2	21.2	21.5	24.4

Table S6: Absolute errors (Hz) between calculated and experimental J-coupling constants for valine residues V in VVV (central), GD^{P}VG, GSVG, and GAVG in the Gaussian model and the three MD-force fields. χ^2_J denotes the reduced chi-squared value. The Gaussian Ramachandran distribution of valine residue in VVV is taken from Schweitzer-Stenner,[S1] whereas Gaussian Ramachandran distributions of valine residues in GD^{P}VG, GSVG, and GAVG peptides are reproduced from Schweitzer-Stenner[S6] with permission from the Royal Society of Chemistry. The Gaussian model and MD-derived Ramachandran distributions of valine in GVG peptide are reproduced from Andrews et al.[S3] with permission from the Royal Society of Chemistry and Suresh et al. (Copyright 2025 American Chemical Society),[S4] respectively.

**Peptide**	**Model**	$^3J(H^N, H^{C_\alpha})$ (Hz)	$^3J(H^N, H^{C'})$ (Hz)	$^3J(H^{C_\alpha}, C')$ (Hz)	$^3J(N, H^{C_\beta})$ (Hz)	$^1J(N, C^\alpha)$ (Hz)	χ^2_J
V**V**V	Gaussian	0.154	0.568	0.177	0.404	0.135	15.070
	C36m	1.177	0.252	0.498	0.404	0.169	29.154
	ff14SB	1.869	0.422	0.769	0.583	0.449	90.601
	ff24EXP-GA	0.838	0.391	0.430	0.156	1.045	154.619
GD^{P}**V**G	Gaussian	0.220	0.025	0.162	0.001	0.068	0.7188
	C36m	1.586	0.107	0.894	0.487	0.345	46.232
	ff14SB	1.735	0.265	1.026	0.438	0.294	55.636
	ff24EXP-GA	0.670	0.236	0.638	0.005	0.479	14.722
GS**V**G	Gaussian	0.443	0.010	0.341	0.078	0.095	3.469
	C36m	1.724	0.201	1.640	0.306	0.331	104.287
	ff14SB	1.744	0.308	1.697	0.238	0.414	131.093
	ff24EXP-GA	0.903	0.333	1.262	0.141	0.118	67.384
GA**V**G	Gaussian	0.065	0.232	0.258	0.020	0.112	1.948
	C36m	0.544	0.246	0.492	0.294	0.528	7.369
	ff14SB	0.637	0.064	0.391	0.211	0.299	5.164
	ff24EXP-GA	0.401	0.000	0.155	0.279	0.155	2.530

Table S7: Mesostate populations (%) for serine residue S in GSG, GSAG, GSLG, and GSVG peptides in the Gaussian model and the three MD force fields. The Gaussian Ramachandran distributions for GSAG, GSLG, and GSVG used in this analysis are reproduced from Schweitzer-Stenner[S6] with permission from the Royal Society of Chemistry. The Gaussian model and MD-derived Ramachandran distributions of serine in GSG peptide are reproduced from Andrews et al.[S3] with permission from the Royal Society of Chemistry and Suresh et al.[S4] (Copyright 2025 American Chemical Society), respectively.

Peptide	Mesostate	Gaussian	C36m	ff14SB	ff24EXP-GA
GSG	pPII	33.3	38.6	44.4	36.9
	β-strand	32.2	33.2	28.6	22.4
	ε	10.9	0.5	1.7	10.0
	turn	23.6	27.7	25.3	30.7
GSAG	pPII	24.4	44.1	50.6	35.9
	β-strand	29.4	32.7	33.9	30.8
	ε	22.4	0.5	2.0	11.7
	turn	23.8	22.7	13.5	21.6
GSLG	pPII	35.1	45.0	42.4	28.7
	β-strand	41.8	32.8	26.3	25.5
	ε	11.0	0.5	1.4	10.2
	turn	12.1	21.7	29.9	35.6
GSVG	pPII	31.8	42.1	49.8	31.7
	β-strand	31.1	30.4	31.2	28.8
	ε	13.3	0.7	2.0	11.4
	turn	23.8	26.9	17.0	28.1

Table S8: Absolute errors (Hz) between calculated and experimental J-coupling constants for serine residue S in GSAG, GSLG, and GSVG peptides in the Gaussian model and the three MD force fields. χ^2_J denotes the reduced chi-squared value. The Gaussian Ramachandran distributions for serine residues in GSAG, GSLG, and GSVG are reproduced from Schweitzer-Stenner[S6] with permission from the Royal Society of Chemistry. The Gaussian model and MD-derived Ramachandran distributions of serine in GSG peptide are reproduced from Andrews et al.[S3] with permission from the Royal Society of Chemistry and Suresh et al. (Copyright 2025 American Chemical Society),[S4] respectively.

**Peptide**	**Model**	$^3J(H^N, H^{C_\alpha})$ (Hz)	$^3J(H^N, H^{C'})$ (Hz)	$^3J(H^{C_\alpha}, C')$ (Hz)	$^3J(N, H^{C_\beta})$ (Hz)	$^1J(N, C^\alpha)$ (Hz)	χ^2_J
G**S**AG	Gaussian	0.239	0.020	0.517	0.236	0.509	5.673
	C36m	0.289	0.395	1.206	0.290	1.061	58.323
	ff14SB	0.459	0.435	1.522	0.255	0.796	78.504
	ff24EXP-GA	0.255	0.213	1.174	0.406	0.519	41.839
G**S**LG	Gaussian	0.267	0.454	0.067	0.188	0.494	1.738
	C36m	0.263	0.858	0.340	0.251	1.073	42.440
	ff14SB	0.502	0.863	0.701	0.161	1.093	56.658
	ff24EXP-GA	0.356	0.656	0.432	0.391	0.800	29.162
G**S**VG	Gaussian	0.072	0.008	0.652	0.246	0.400	7.523
	C36m	0.344	0.433	1.308	0.386	1.113	85.442
	ff14SB	0.554	0.487	1.736	0.346	0.840	113.625
	ff24EXP-GA	0.173	0.400	0.877	0.579	0.450	38.011

Table S9: Mesostate populations (%) for protonated aspartic acid residues $\mathrm{D^P}$ in $\mathrm{GD^PG}$, $\mathrm{GD^PLG}$, and $\mathrm{GD^PVG}$ peptides in the Gaussian model and the three MD force fields. The Gaussian Ramachandran distributions for protonated aspartic acid residues in $\mathrm{GD^PLG}$ and $\mathrm{GD^PVG}$ are reproduced from Schweitzer-Stenner[S6] with permission from the Royal Society of Chemistry. The Gaussian model and MD-derived Ramachandran distributions of protonated aspartic acid residue in $\mathrm{GD^PG}$ peptide are reproduced from Andrews et al.[S3] with permission from the Royal Society of Chemistry and Suresh et al. (Copyright 2025 American Chemical Society),[S4] respectively.

Peptide	Mesostate	Gaussian	C36m	ff14SB	ff24EXP-GA
$\mathrm{GD^PG}$	pPII	7.9	36.5	48.7	29.6
	β-strand	24.3	36.3	22.4	24.0
	ε	34.0	0.5	0.8	7.4
	turn	33.8	26.7	28.1	39.0
$\mathrm{GD^PLG}$	pPII	38.0	39.7	46.2	31.9
	β-strand	45.1	36.7	21.7	29.2
	ε	4.2	0.4	0.8	7.7
	turn	12.7	23.2	31.3	31.2
$\mathrm{GD^PVG}$	pPII	28.9	39.1	48.3	31.3
	β-strand	31.5	35.2	22.6	29.7
	ε	17.8	0.4	0.8	7.7
	turn	21.9	25.3	28.3	31.2

Table S10: Absolute errors (Hz) between calculated and experimental J-coupling constants for protonated aspartic acid residues $\mathrm{D^P}$ in in $\mathbf{GD^PLG}$ and $\mathbf{GD^PVG}$ peptides in the Gaussian model and the three MD force fields. χ^2_J denotes the reduced chi-squared value. The Gaussian Ramachandran distributions of protonated aspartic acid resiudes in $\mathbf{GD^P}$LG and $\mathbf{GD^P}$VG are reproduced from Schweitzer-Stenner[S6] with permission from the Royal Society of Chemistry.

**Peptide**	**Model**	$^3\mathrm{J}(\mathrm{H^N}, \mathrm{H^{C_\alpha}})$ (Hz)	$^3\mathrm{J}(\mathrm{H^N}, \mathrm{H^{C'}})$ (Hz)	$^3\mathrm{J}(\mathrm{H^{C_\alpha}}, \mathrm{C'})$ (Hz)	$^3\mathrm{J}(\mathrm{N}, \mathrm{H^{C_\beta}})$ (Hz)	$^1\mathrm{J}(\mathrm{N}, \mathrm{C^\alpha})$ (Hz)	χ^2_J
$\mathrm{G\mathbf{D^P}LG}$	Gaussian	0.313	0.142	0.493	0.168	0.233	6.131
	C36m	0.373	0.065	0.429	0.124	0.241	7.080
	ff14SB	0.463	0.244	0.982	0.103	0.315	15.266
	ff24EXP-GA	0.336	0.259	0.311	0.241	0.049	3.331
$\mathrm{G\mathbf{D^P}VG}$	Gaussian	0.242	0.010	0.232	0.007	0.174	0.9241
	C36m	0.207	0.079	0.416	0.046	0.812	53.984
	ff14SB	0.490	0.375	0.582	0.050	0.837	63.083
	ff24EXP-GA	0.209	0.281	0.430	0.192	0.344	12.197

Table S11: Mesostate populations (%) for protonated aspartic acid residues in GD^{P}G, GD^{P}D^{P}G, and GD^{P}D^{P}D^{P}G peptides in the Gaussian model and the three MD force fields. The Gaussian Ramachandran distributions of all aspartic acid residues in GD^{P}D^{P}G and GD^{P}D^{P}D^{P}G peptides are taken from Milorey et al.[S5] The Gaussian model and MD-derived Ramachandran distributions of protonated aspartic acid residue in GD^{P}G peptide are taken from Andrews et al.[S3] with permission from the Royal Society of Chemistry and Suresh et al. (Copyright 2025 American Chemical Society),[S4] respectively.

**Peptide**	**Mesostate**	**Gaussian**	**C36m**	**ff14SB**	**ff24EXP-GA**
GD$^{\mathbf{P}}$G (bold D$^{\mathbf{P}}$)	pPII	7.9	36.5	48.7	29.6
	β-strand	24.3	36.3	22.4	24.0
	ε	34.0	0.5	0.8	7.4
	turn	33.8	26.7	28.1	39.0
G**D$^{\mathbf{P}}$**D^{P}G	pPII	18.9	34.8	53.8	24.1
	β-strand	31.6	35.5	21.9	22.1
	ε	5.1	0.3	0.8	5.4
	turn	44.4	29.3	23.5	48.4
GD^{P}**D$^{\mathbf{P}}$**G	pPII	9.0	30.9	49.8	24.9
	β-strand	57.4	33.7	23.7	34.2
	ε	6.6	0.5	1.1	8.7
	turn	27.0	34.9	25.4	32.2
G**D$^{\mathbf{P}}$**D^{P}D^{P}G	pPII	23.3	31.6	53.5	28.6
	β-strand	39.8	33.6	27.3	24.9
	ε	3.9	0.3	0.4	6.7
	turn	33.0	34.5	18.8	39.8
GD^{P}**D$^{\mathbf{P}}$**D^{P}G	pPII	21.7	30.2	10.6	7.2
	β-strand	35.5	35.9	4.4	11.6
	ε	5.0	0.4	0.1	2.6
	turn	37.7	33.5	84.9	78.5
GD^{P}D^{P}**D$^{\mathbf{P}}$**G	pPII	13.7	29.7	34.7	19.1
	β-strand	44.1	34.0	26.7	35.9
	ε	3.4	0.5	1.6	9.1
	turn	38.8	35.9	37.1	35.8

Table S12: Absolute errors (Hz) between calculated and experimental J-coupling constants for the two protonated aspartic acid residues $\mathrm{D^P}$ in $\mathrm{GD^PD^PG}$ peptide in the Gaussian model and the three MD force fields. χ^2_J denotes the reduced chi-squared value. The Gaussian Ramachandran distributions of aspartic acid residues in $\mathrm{GD^PD^PG}$ are taken from Milorey et al. (Copyright 2021 American Chemical Society).[S5]

**Peptide**	**Model**	$^3\mathrm{J}(\mathrm{H^N}, \mathrm{H^{C_\alpha}})$ (Hz)	$^3\mathrm{J}(\mathrm{H^N}, \mathrm{H^{C'}})$ (Hz)	$^3\mathrm{J}(\mathrm{H^{C_\alpha}}, \mathrm{C'})$ (Hz)	$^3\mathrm{J}(\mathrm{N}, \mathrm{H^{C_\beta}})$ (Hz)	$^1\mathrm{J}(\mathrm{N}, \mathrm{C^\alpha})$ (Hz)	χ^2_J
G**D^P**D^PG	Gaussian	0.006	0.271	0.155	0.095	0.180	4.834
	C36m	0.161	0.344	0.486	0.055	0.679	16.041
	ff14SB	0.863	0.531	1.173	0.245	0.577	52.798
	ff24EXP-GA	0.247	0.729	0.203	0.135	0.747	31.356
GD^P**D^P**G	Gaussian	0.004	0.143	0.078	0.166	0.104	0.413
	C36m	0.326	0.601	0.596	0.326	0.524	10.418
	ff14SB	1.112	0.466	0.513	0.538	0.417	21.179
	ff24EXP-GA	0.035	0.521	0.049	0.080	0.094	4.476

Table S13: Absolute errors (Hz) between calculated and experimental J-coupling constants for protonated aspartic acid residues in $\mathrm{GD^PD^PD^PG}$ peptide in the Gaussian model and the three MD force fields. χ^2_J denotes the reduced chi-squared value. The Gaussian Ramachandran distributions of all three aspartic acid residues in $\mathrm{GD^PD^PD^PG}$ peptide are taken from Milorey et al. (Copyright 2025 American Chemical Society).[S5]

Peptide	Model	$^3\mathrm{J}(\mathrm{H^N},\mathrm{H^{C_\alpha}})$ (Hz)	$^3\mathrm{J}(\mathrm{H^N},\mathrm{H^{C'}})$ (Hz)	$^3\mathrm{J}(\mathrm{H^{C_\alpha}},\mathrm{C'})$ (Hz)	$^3\mathrm{J}(\mathrm{N},\mathrm{H^{C_\beta}})$ (Hz)	$^1J(\mathrm{N},\mathrm{C^\alpha})$ (Hz)	χ^2_J
$\mathrm{G\mathbf{D^P}D^PD^PG}$	Gaussian	0.026	0.198	0.167	0.108	0.183	1.470
	C36m	0.119	0.085	0.009	0.138	0.777	7.921
	ff14SB	0.792	0.320	0.849	0.346	0.752	31.290
	ff24EXP-GA	0.794	0.717	0.375	0.083	0.704	20.607
$\mathrm{GD^P\mathbf{D^P}D^PG}$	Gaussian	0.169	0.124	0.942	0.130	0.203	3.685
	C36m	0.023	0.055	1.546	0.021	0.449	7.579
	ff14SB	0.637	0.971	4.645	0.287	1.261	99.768
	ff24EXP-GA	0.409	0.714	3.439	0.256	0.753	52.979
$\mathrm{GD^PD^P\mathbf{D^P}G}$	Gaussian	0.434	0.030	0.715	0.059	0.277	7.179
	C36m	0.317	0.113	0.185	0.134	0.534	4.869
	ff14SB	0.874	0.530	0.832	0.220	0.483	28.600
	ff24EXP-GA	0.021	0.438	0.091	0.502	0.145	17.115

Supporting Figures

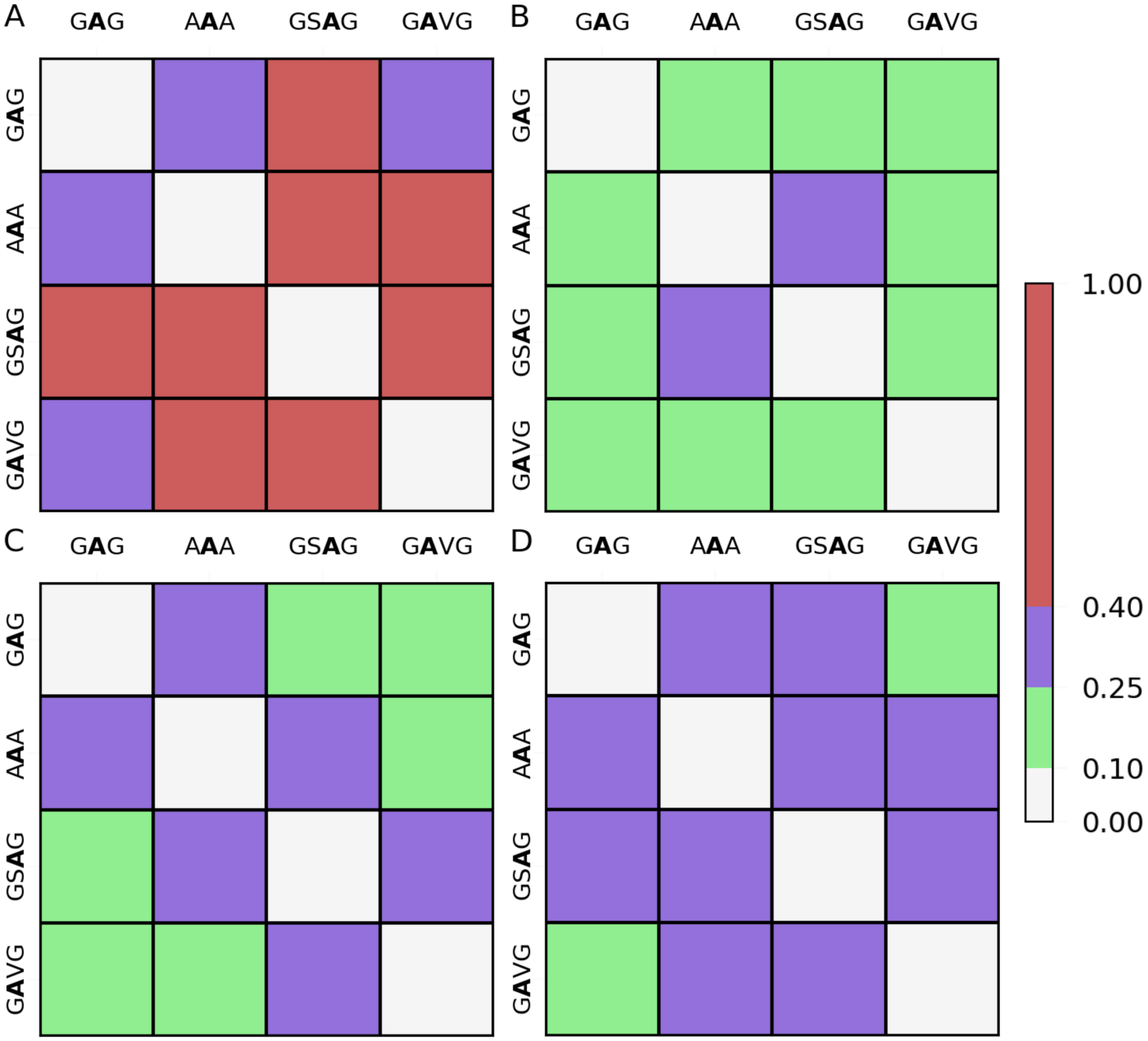

Figure S1: Hellinger distances between pairs of Ramachandran distributions of alanine residue in alanine residue-containing peptides, derived from (A) the Gaussian model, (B) CHARMM36m, (C) Amber ff14SB, and (D) Amber ff24EXP-GA simulations. The red, purple, green and, white colors correspond to very dissimilar, moderately dissimilar, moderately similar, and very similar Ramachandran distributions, respectively. The Gaussian Ramachandran distributions of alanine residue in G**A**G and A**A**A peptides, and alanine residue in GS**A**G and G**A**VG peptides used in this calculation are reproduced from Zhang et al. (Copyright 2020 American Chemical Society)[S2] and from Schweitzer-Stenner[S6] with permission from the Royal Society of Chemistry, respectively. MD-derived Ramachandran distributions for alanine residue in G**A**G peptide used in this calculation are taken from Suresh et al. (Copyright 2025 American Chemical Society).[S4]

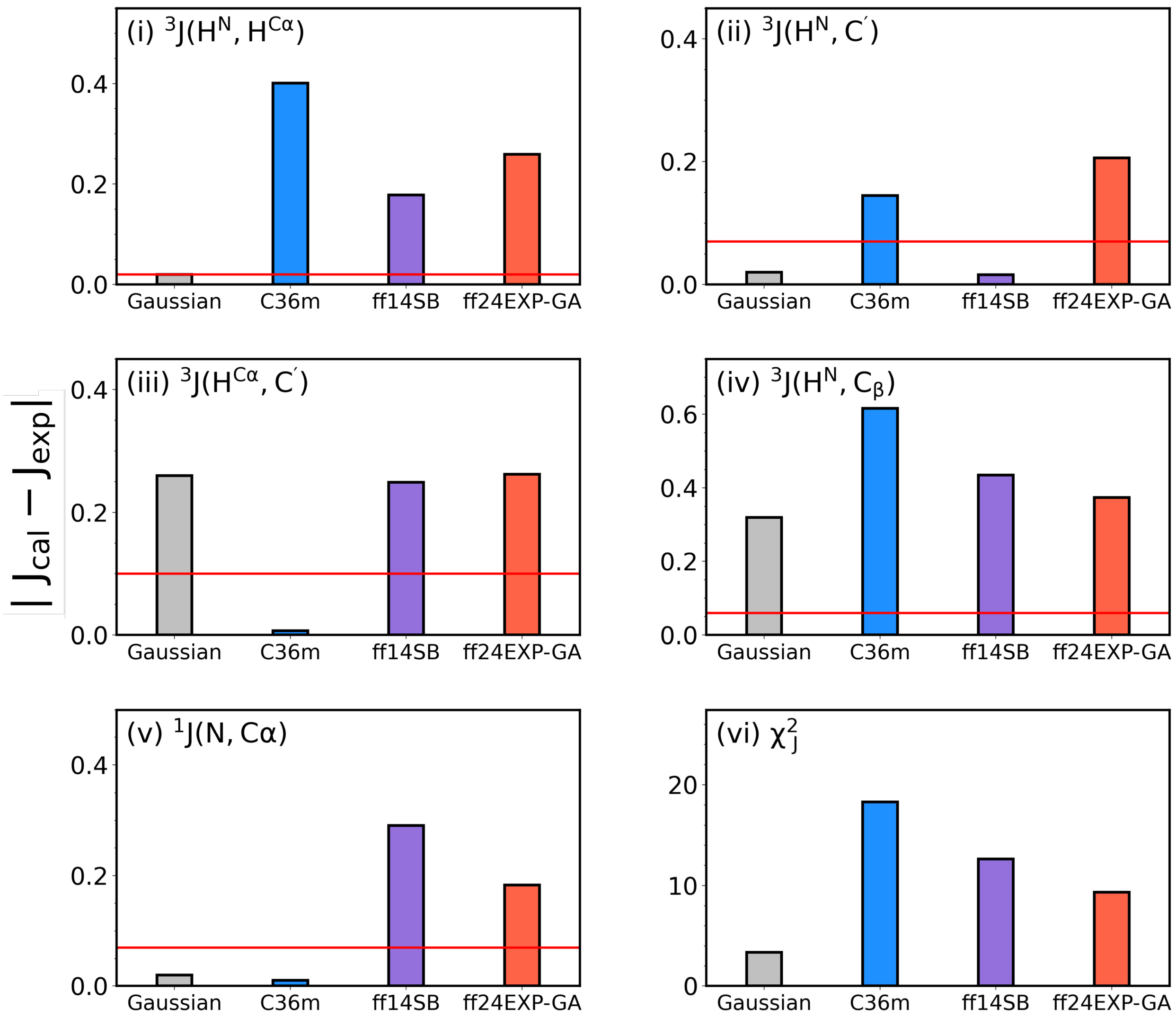

Figure S2: Comparison between experimental and computed J-coupling constants and amide I' profiles of the central alanine residue in A**A**A. (i-v) Absolute differences between calculated and experimental values of the five J-coupling constants for the Gaussian model and the three MD force fields. Red lines correspond to experimental uncertainties. (vi) Reduced $\chi^2{}_J$ values. The Gaussian Ramachandran distribution used in this calculation is taken from Zhang et al. (Copyright 2020 American Chemical Society).[S2]

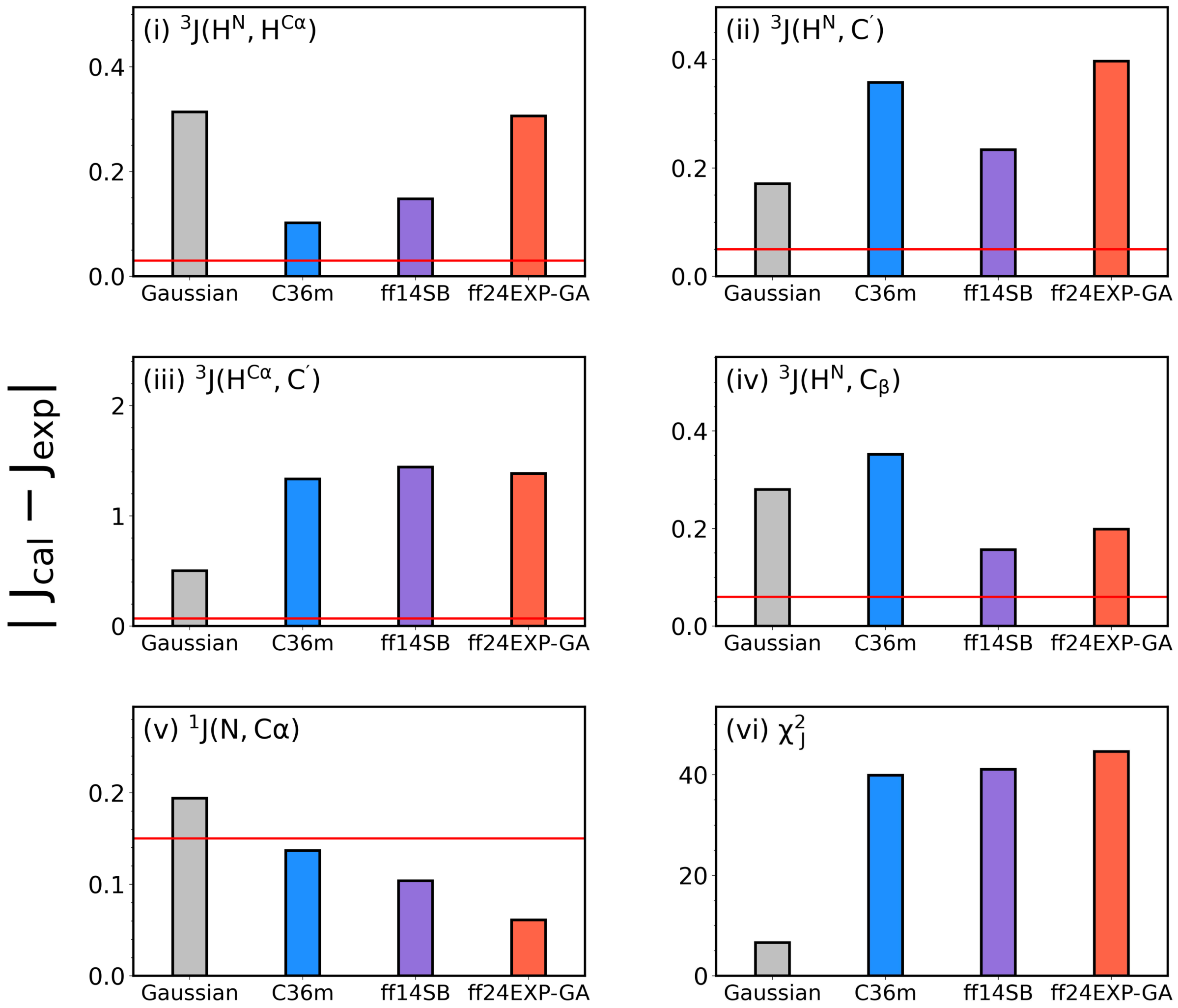

Figure S3: Comparison of experimental and computed J-coupling constants of alanine residue in GS**A**G peptide. (i-v) Absolute differences between calculated and experimental values of the five J-coupling constants for the Gaussian model and the three MD force fields. Red lines correspond to experimental uncertainties. (vi) Reduced $\chi^2{}_{\mathrm{J}}$ values. The Gaussian Ramachandran distribution used in this calculation is reproduced from Schweitzer-Stenner[S6] with permission from the Royal Society of Chemistry.

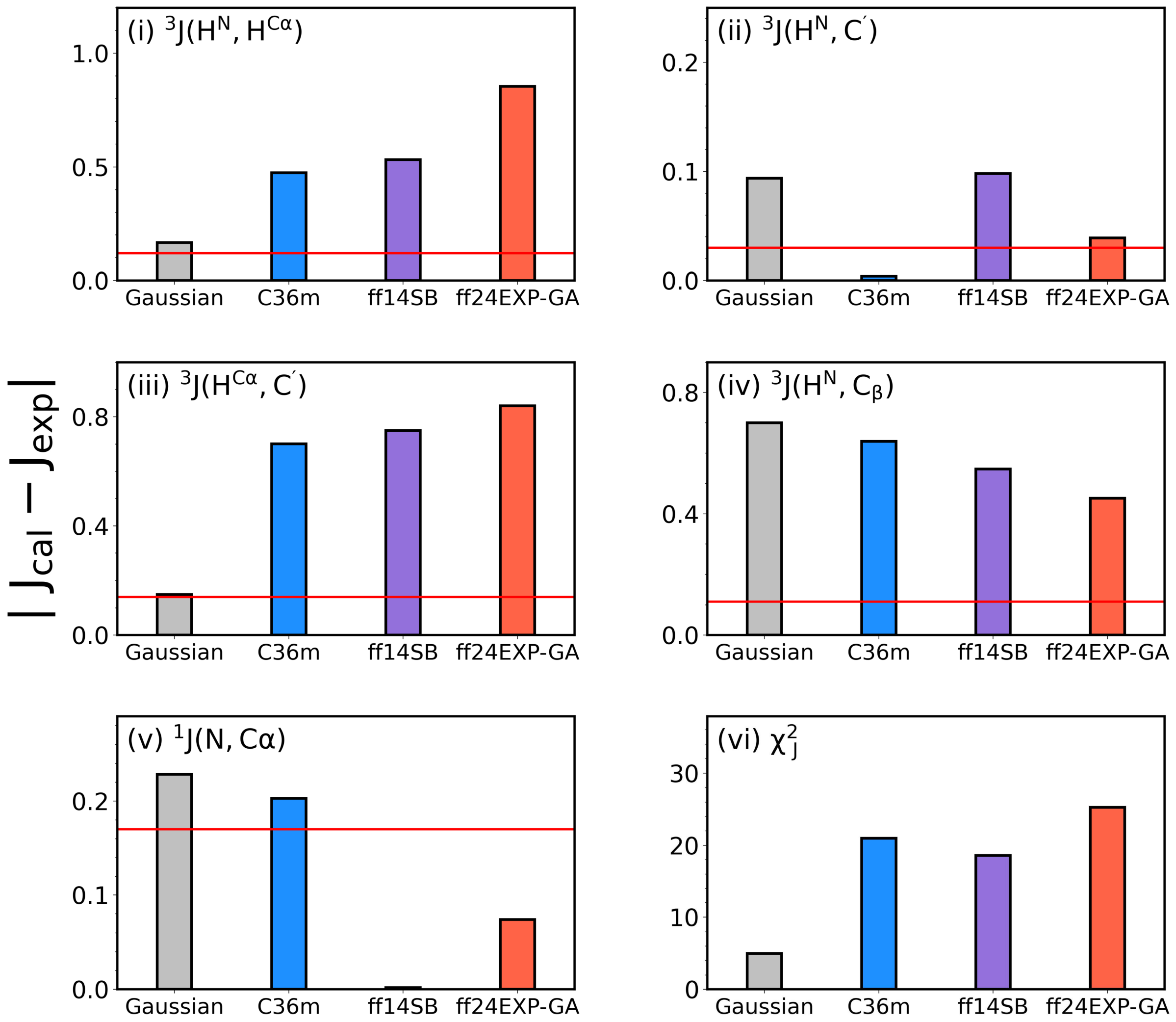

Figure S4: Comparison of experimental and computed J-coupling constants of alanine residue in G**A**VG peptide. (i-v) Absolute differences between calculated and experimental values of the five J-coupling constants for the Gaussian model and the three MD force fields. Red lines correspond to experimental uncertainties. (vi) Reduced $\chi^2{}_J$ values. The Gaussian model Ramachandran distribution used in this calculation is reproduced from Schweitzer-Stenner[S6] with permission from the Royal Society of Chemistry.

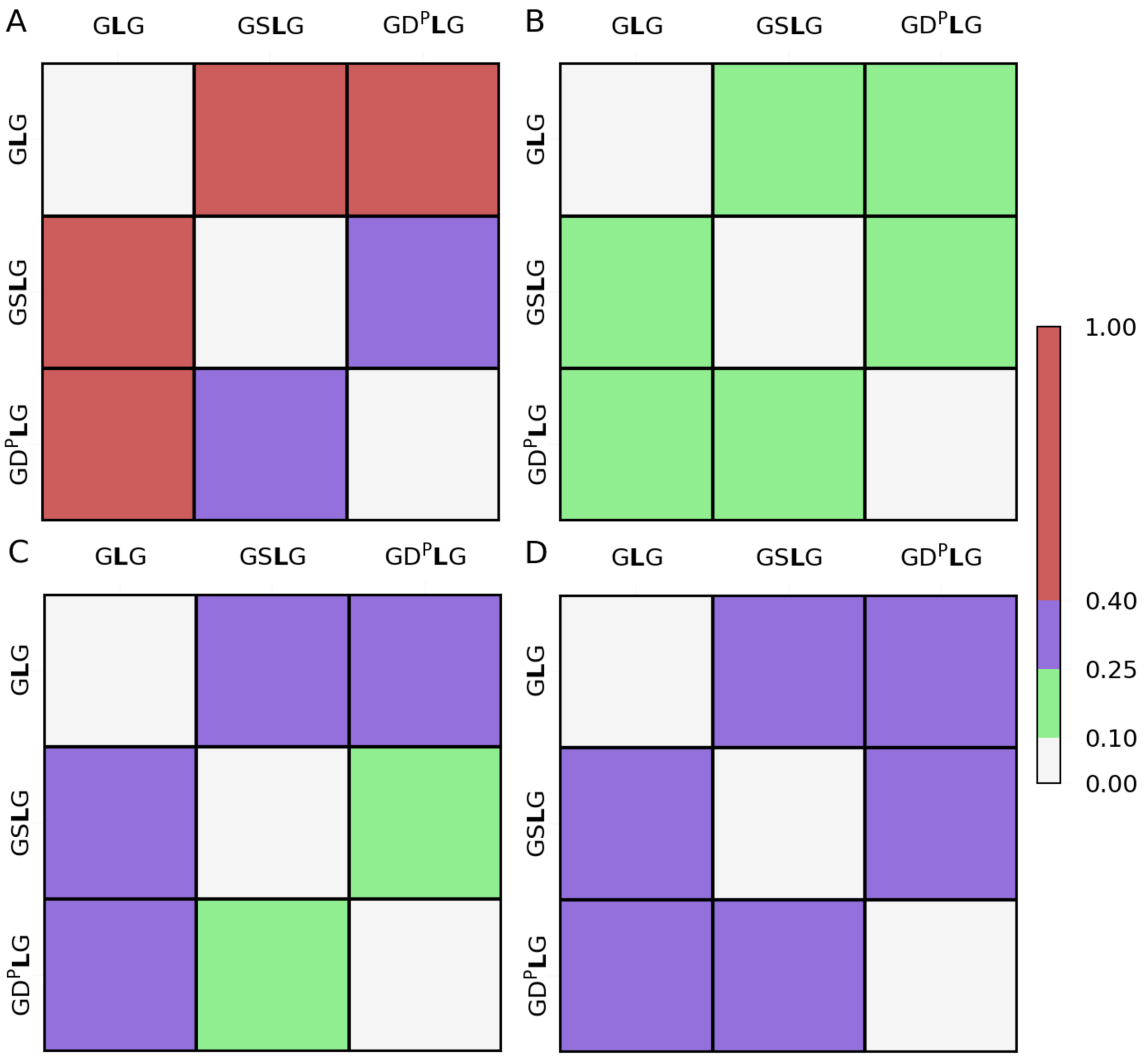

Figure S5: Hellinger distances between pairs of Ramachandran distributions of leucine residue in leucine residue-containing peptides, derived from (A) the Gaussian model, (B) CHARMM36m, (C) Amber ff14SB, and (D) Amber ff24EXP-GA simulations. The red, purple, green, and white colors correspond to very dissimilar, moderately dissimilar, moderately similar, and very similar Ramachandran distributions, respectively. The Gaussian and MD-derived Ramachandran distributions for leucine residue in GLG peptide used in this calculation are taken from Schweitzer-Stenner (Copyright 2009 American Chemical Society)[S1] and Suresh et al. (Copyright 2025 American Chemical Society),[S4] respectively. The Gaussian Ramachandran distributions for GS**L**G and GD^{P}**L**G peptides used in this calculation are reproduced from Schweitzer-Stenner[S6] with permission from the Royal Society of Chemistry.

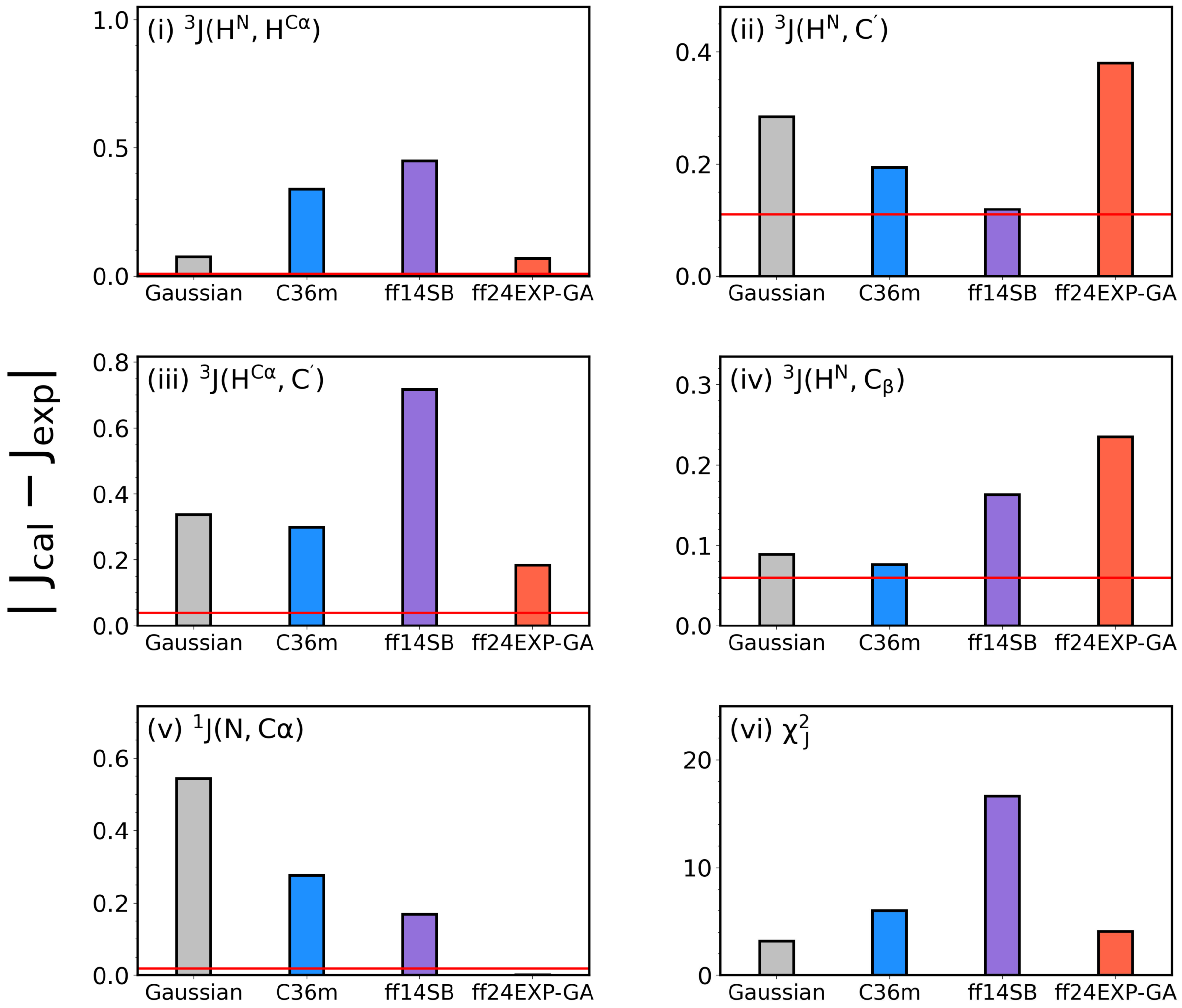

Figure S6: Comparison of experimental and computed J-coupling constants of leucine residue in GS**L**G peptide. (i-v) Absolute differences between calculated and experimental values of the five J-coupling constants for the Gaussian model and the three MD force fields. Red lines correspond to experimental uncertainties. (vi) Reduced $\chi^2{}_\mathrm{J}$ values. The Gaussian Ramachandran distributions used in this calculation are reproduced from Schweitzer-Stenner[S6] with permission from the Royal Society of Chemistry.

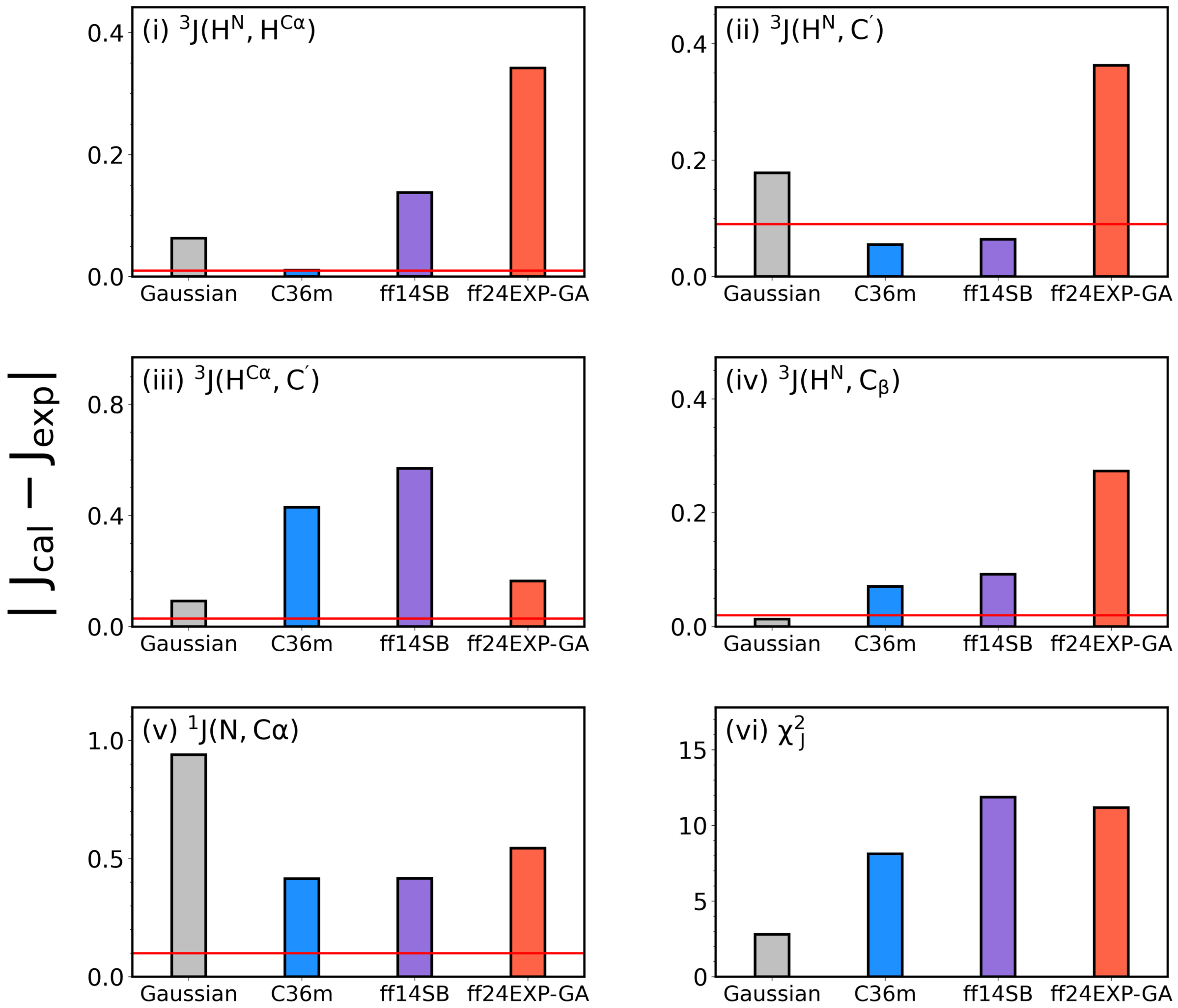

Figure S7: Comparison of experimental and computed J-coupling constants of leucine residue in GD^{P}**L**G peptide. (i-v) Absolute differences between calculated and experimental values of the five J-coupling constants for the Gaussian model and the three MD force fields. Red lines correspond to experimental uncertainties. (vi) Reduced $\chi^2{}_{\mathrm{J}}$ values. The Gaussian Ramachandran distributions used in this calculation are reproduced from Schweitzer-Stenner[S6] with permission from the Royal Society of Chemistry.

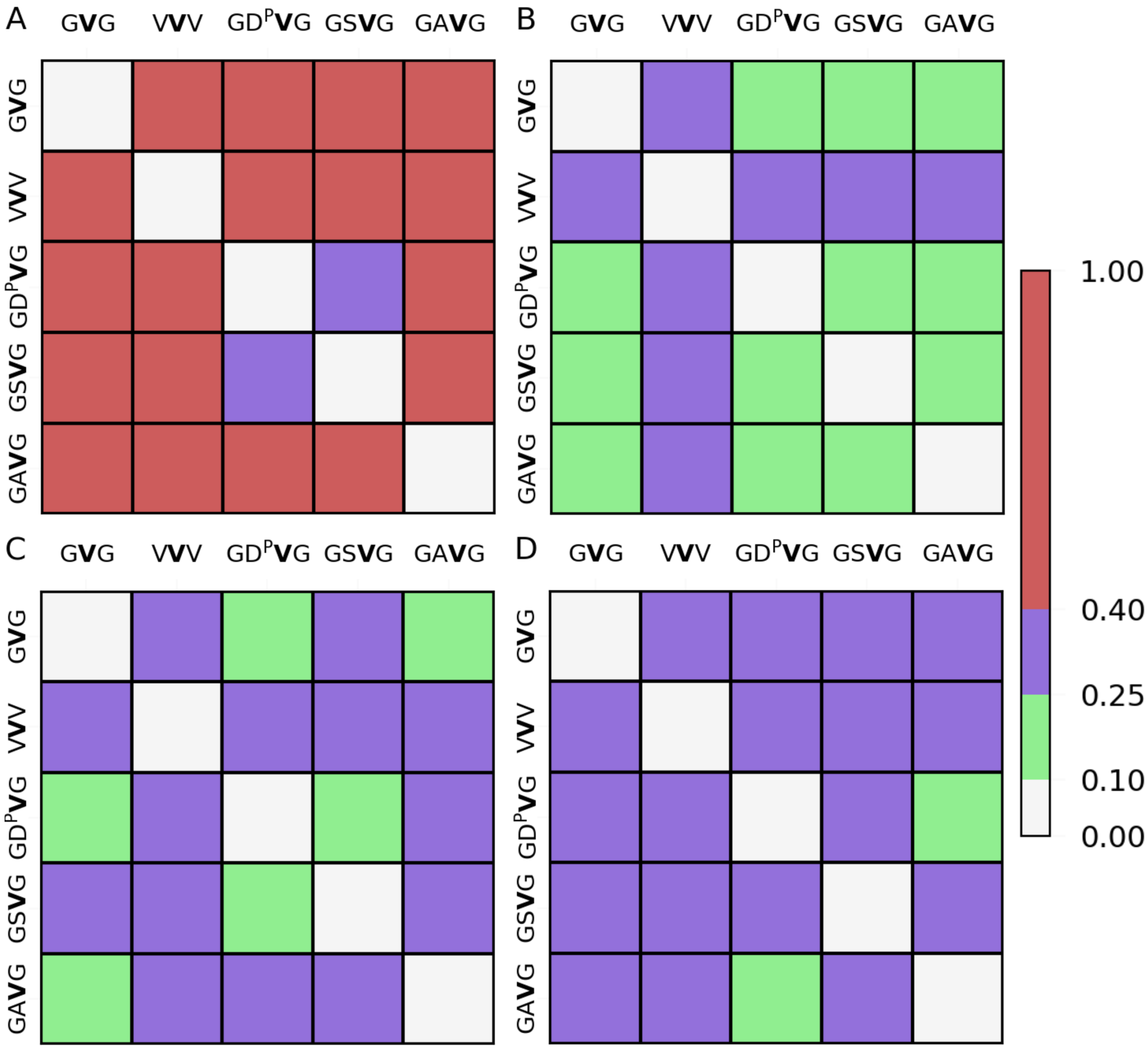

Figure S8: Hellinger distances between pairs of Ramachandran distributions of valine residue in valine-containing peptides, derived from (A) the Gaussian model, (B) CHARMM36m, (C) Amber ff14SB, and (D) Amber ff24EXP-GA simulations. The red, purple, green, and white colors correspond to very dissimilar, moderately dissimilar, moderately similar, and very similar Ramachandran distributions, respectively. The Gaussian Ramachandran distributions for V**V**V, GD^{P}**V**G, GS**V**G, and GA**V**G peptides used in this analysis are taken from Schweitzer-Stenner[S1] and reproduced from Schweitzer-Stenner[S6] with permission from the Royal Society of Chemistry. The Gaussian model and MD-derived Ramachandran distributions of valine residue in GVG peptide used in this calculation are reproduced from Andrews et al.[S3] with permission from the Royal Society of Chemistry and from Suresh et al. (Copyright 2025 American Chemical Society),[S4] respectively.

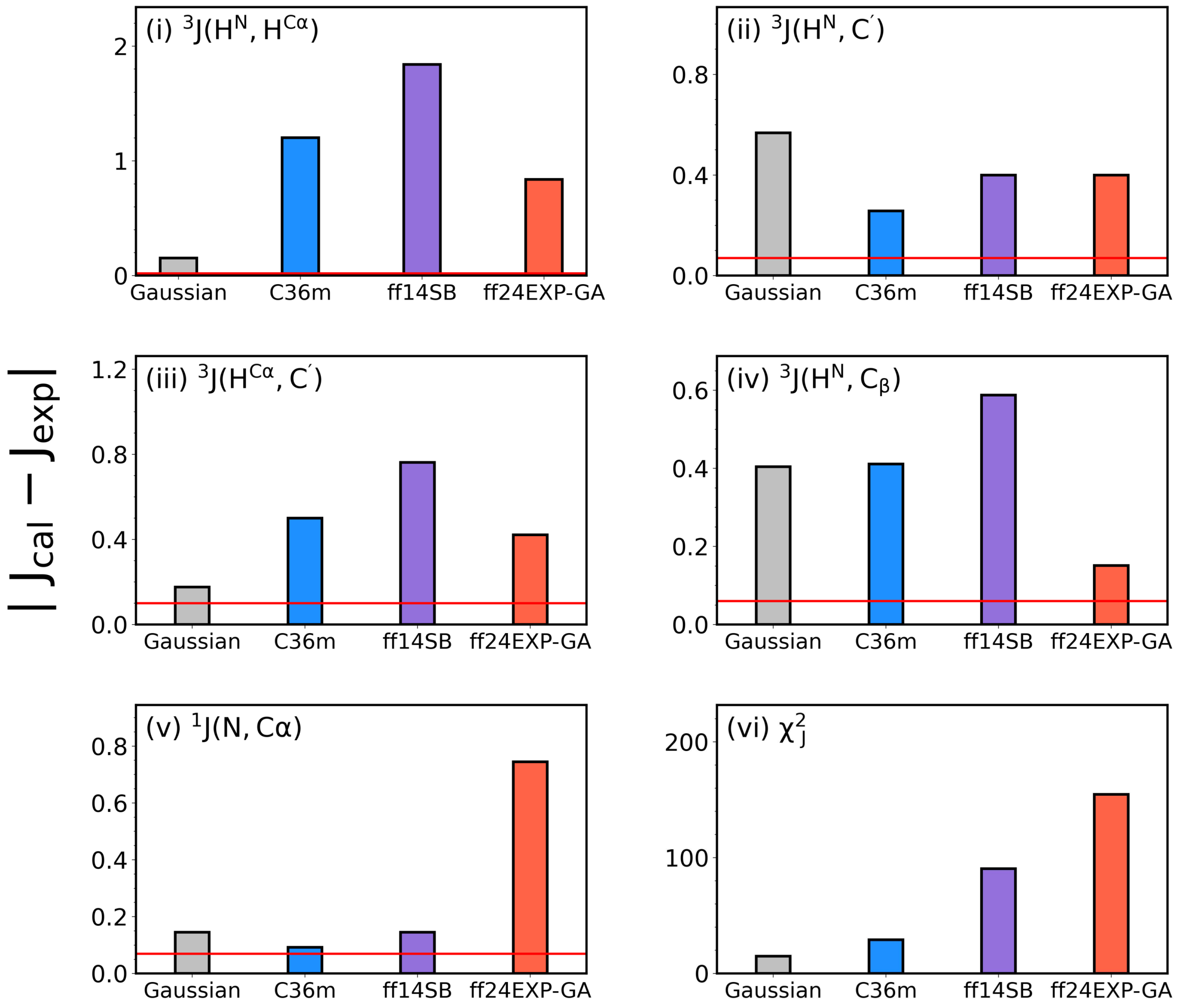

Figure S9: Comparison between experimental and computed J-coupling constants of the central valine residue in V**V**V peptide. (i-v) Absolute differences between calculated and experimental values of the five J-coupling constants for the Gaussian model and the three MD force fields. Red lines correspond to experimental uncertainties. (vi) Reduced ${\chi^2}_J$ values for the Gaussian model and the three MD force fields. The Gaussian Ramachandran distribution used in this calculation is taken from Schweitzer-Stenner (Copyright 2009 American Chemical Society).[S1]

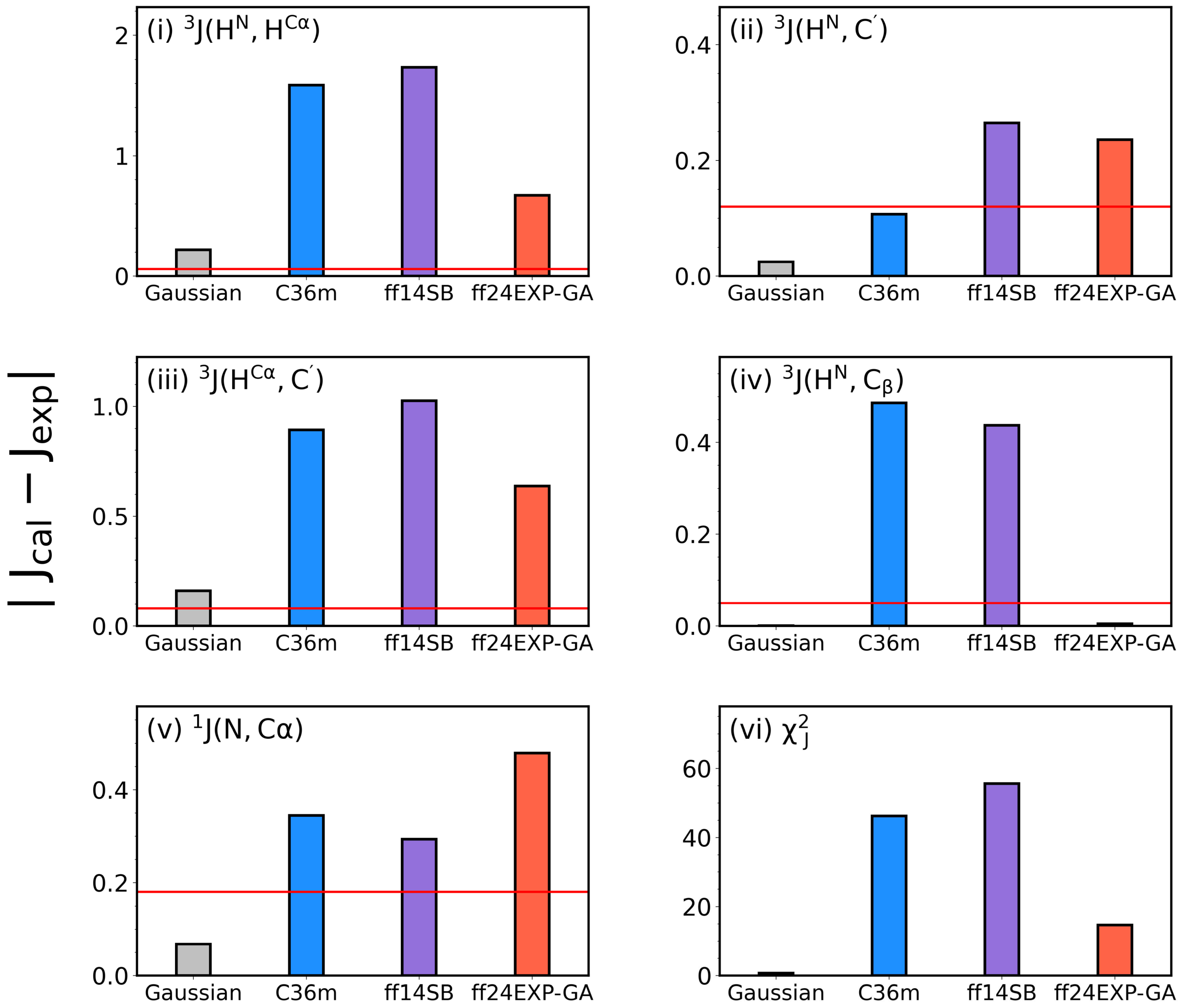

Figure S10: Comparison of experimental and computed J-coupling constants of valine residue in GD^P**V**G peptide. (i-v) Absolute differences between calculated and experimental values of the five J-coupling constants for the Gaussian model and the three MD force fields. Red lines correspond to experimental uncertainties. (vi) Reduced $\chi^2{}_J$ values. The Gaussian Ramachandran distribution for this calculation is reproduced from Schweitzer-Stenner[S6] with permission from the Royal Society of Chemistry.

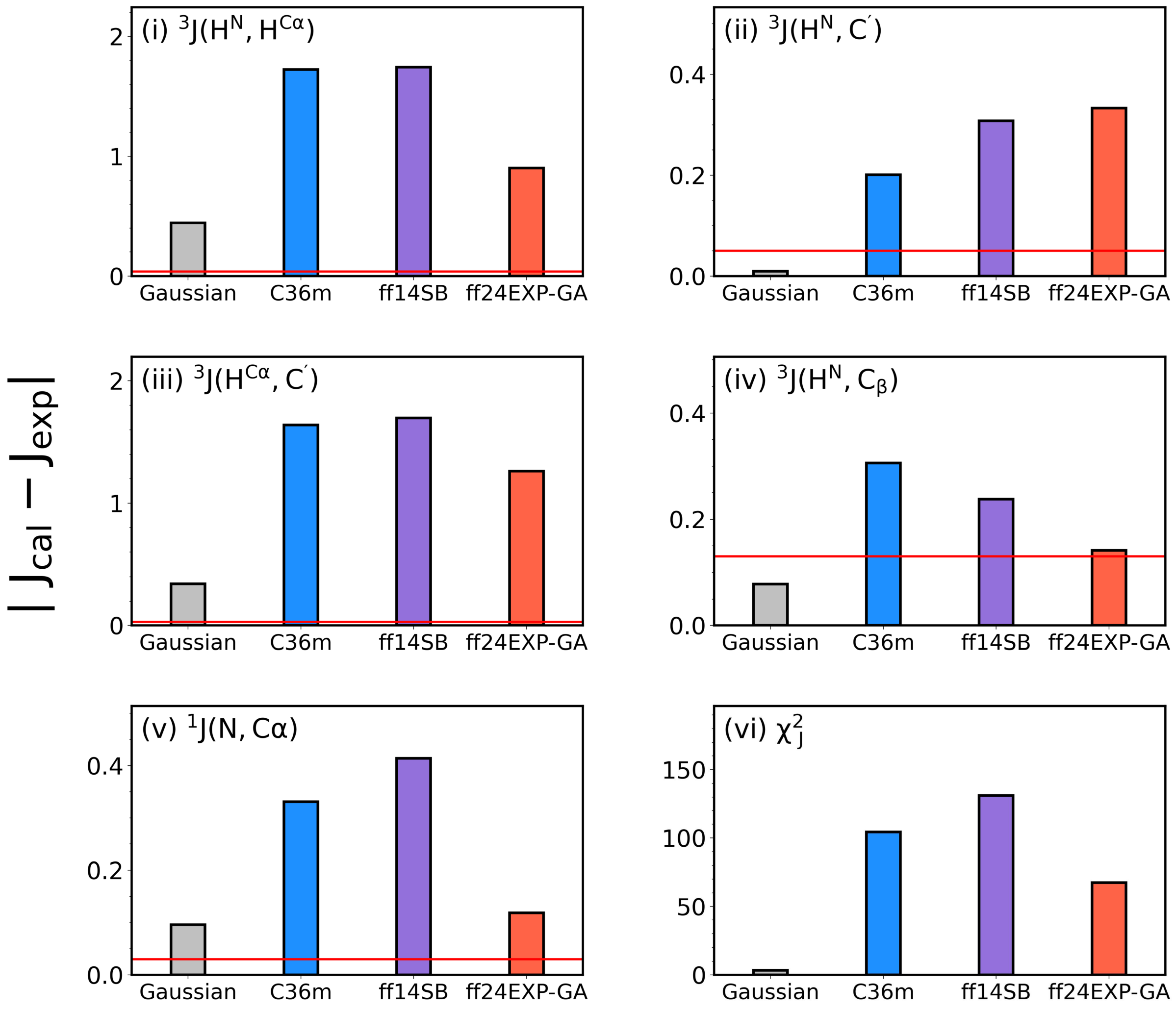

Figure S11: Comparison of experimental and computed J-coupling constants of valine residue in GS**V**G peptide. (i-v) Absolute differences between calculated and experimental values of the five J-coupling constants for the Gaussian model and the three MD force fields. Red lines correspond to experimental uncertainties. (vi) Reduced ${\chi^2}_J$ values. The Gaussian Ramachandran distribution used in this calculation is reproduced from Schweitzer-Stenner[S6] with permission from the Royal Society of Chemistry.

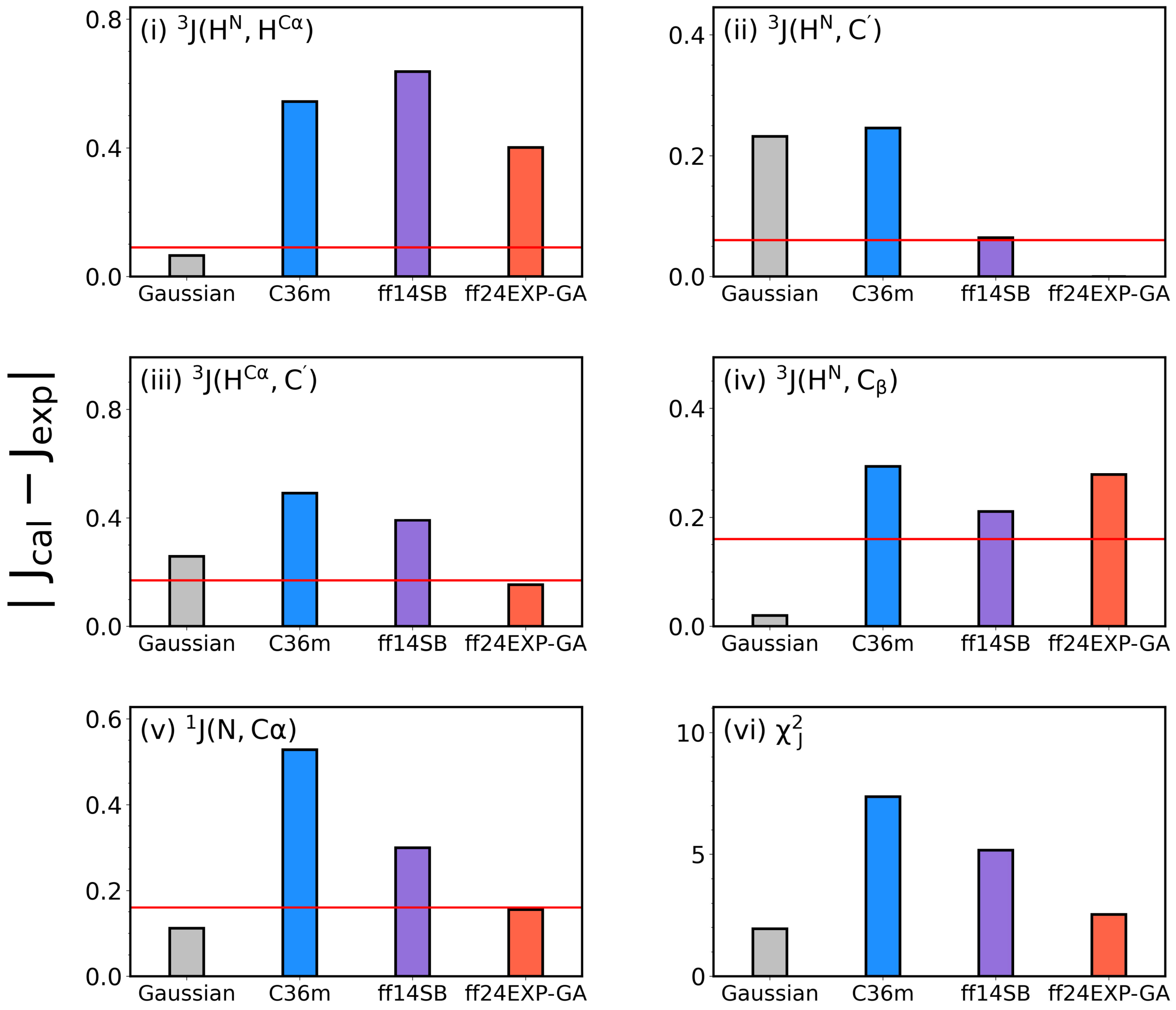

Figure S12: Comparison of experimental and computed J-coupling constants of valine residue in GA**V**G peptide. (i-v) Absolute differences between calculated and experimental values of the five J-coupling constants for the Gaussian model and the three MD force fields. Red lines correspond to experimental uncertainties. (vi) Reduced $\chi^2{}_J$ values. The Gaussian Ramachandran distribution used in this analysis is reproduced from Schweitzer-Stenner[S6] with permission from the Royal Society of Chemistry.

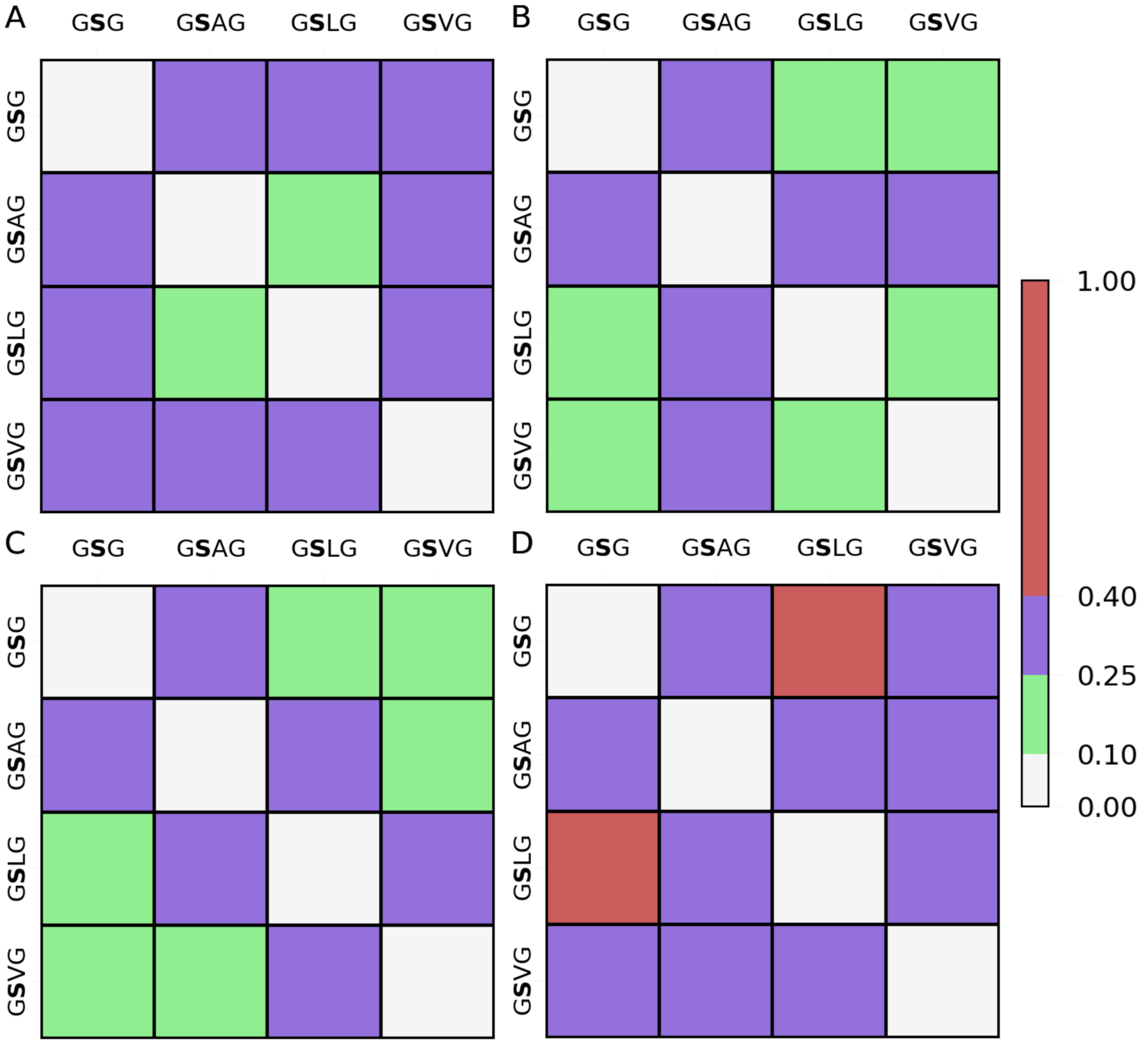

Figure S13: Hellinger distances between pairs of Ramachandran distributions of serine residue in serine residue-containing peptides, derived from (A) the Gaussian model, (B) CHARMM36m, (C) Amber ff14SB, and (D) Amber ff24EXP-GA simulations. The red, purple, green, and white colors correspond to very dissimilar, moderately dissimilar, moderately similar, and very similar Ramachandran distributions, respectively. The Gaussian Ramachandran distributions for G**S**AG, G**S**LG, and G**S**VG peptides used in this analysis are reproduced from Schweitzer-Stenner[S6] with permission from the Royal Society of Chemistry. The Gaussian and MD-derived Ramachandran distributions of serine residue in GSG peptide used in this calculation are reproduced from Andrews et al.[S3] with permission from the Royal Society of Chemistry and from Suresh et al. (Copyright 2025 American Chemical Society),[S4] respectively.

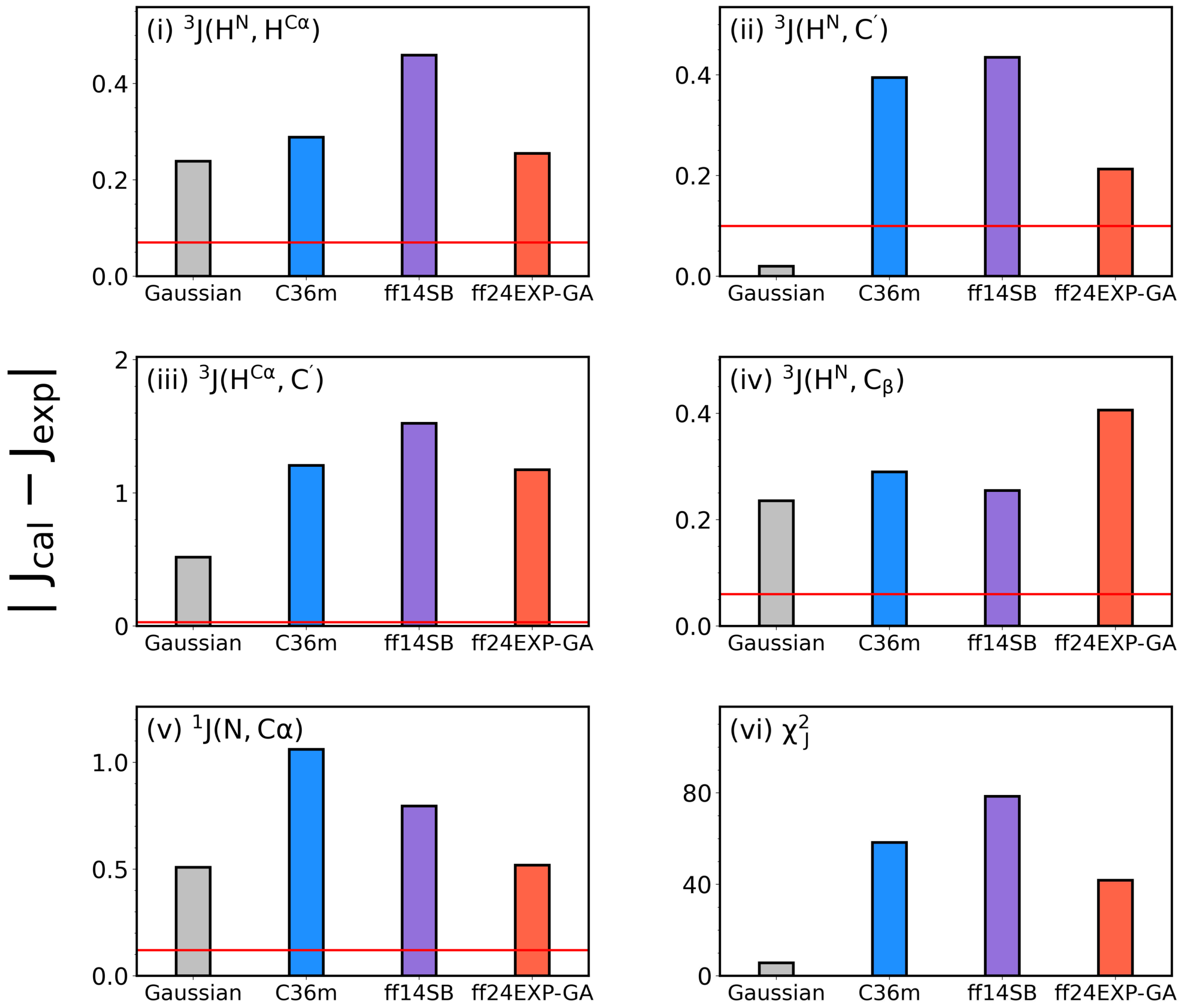

Figure S14: Comparison of experimental and computed J-coupling constants of serine residue in G**S**AG peptide. (i-v) Absolute differences between calculated and experimental values of the five J-coupling constants for the Gaussian model and the three MD force fields. Red lines correspond to experimental uncertainties. (vi) Reduced $\chi^2{}_J$ values. The Gaussian Ramachandran distribution used in this calculation is reproduced from Schweitzer-Stenner[S6] with permission from the Royal Society of Chemistry.

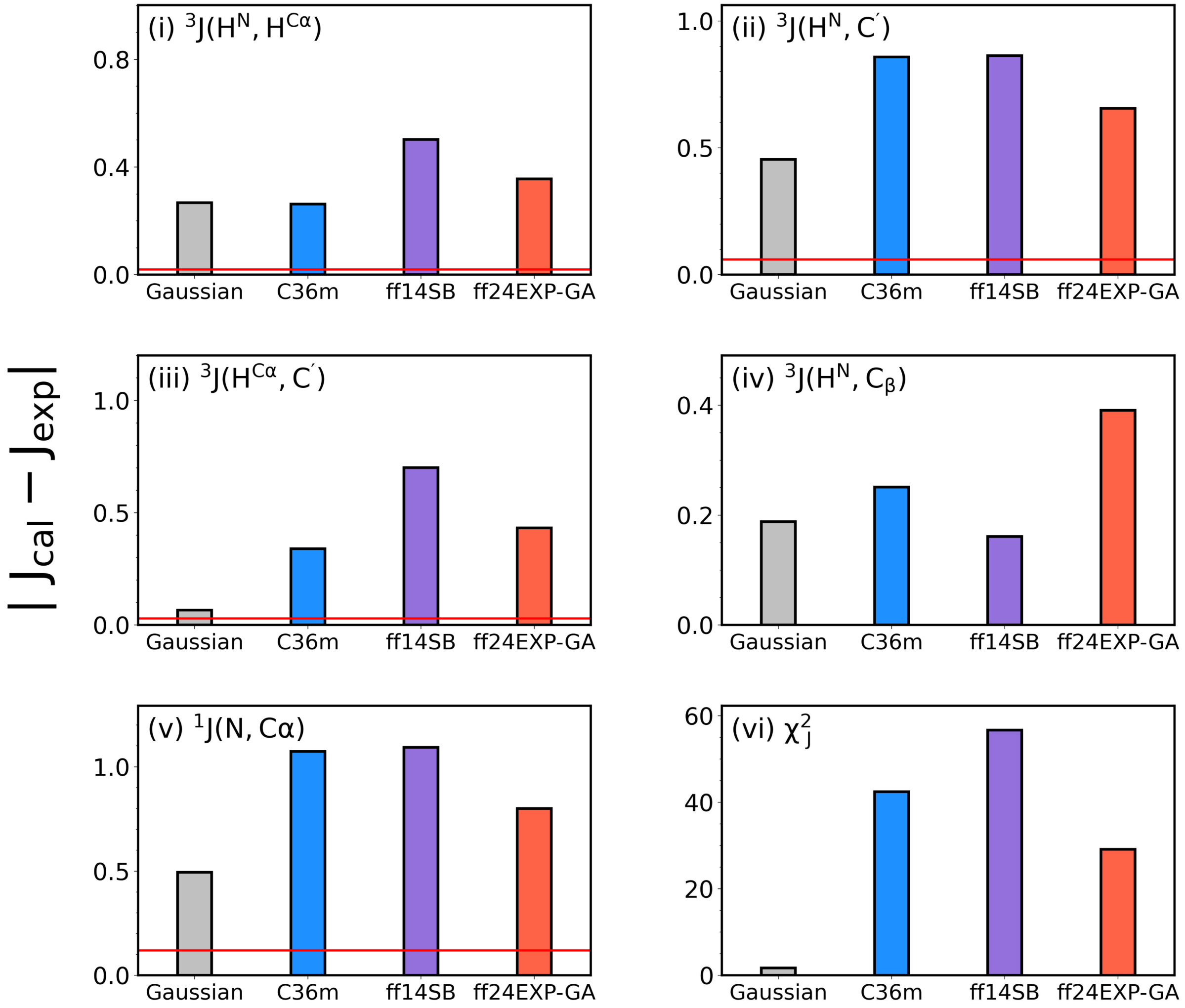

Figure S15: Comparison of experimental and computed J-coupling constants of serine residue in G**S**LG peptide for the Gaussian model and the three MD force fields. (i-v) Absolute differences between calculated and experimental values of the five J-coupling constants for the Gaussian model and the three MD force fields. Red lines correspond to experimental uncertainties. (vi) Reduced $\chi^2{}_J$ values. The Gaussian Ramachandran distribution used in this analysis is reproduced from Schweitzer-Stenner[S6] with permission from the Royal Society of Chemistry.

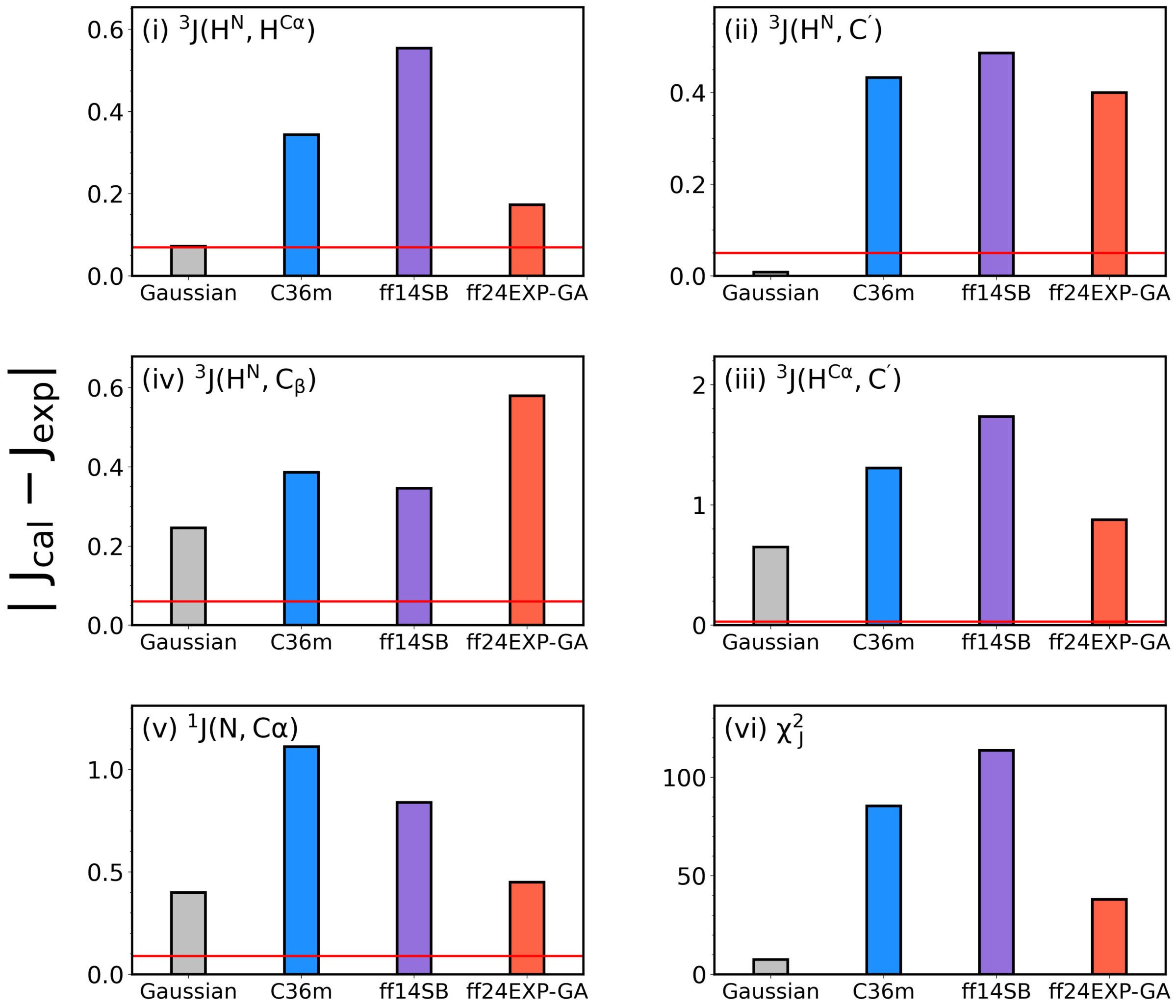

Figure S16: Comparison of experimental and computed J-coupling constants of serine residue in G**S**VG peptide. (i-v) Absolute differences between calculated and experimental values of the five J-coupling constants for the Gaussian model and the three MD force fields. Red lines correspond to experimental uncertainties. (vi) Reduced ${\chi^2}_J$ values. The Gaussian Ramachandran distribution used in this analysis is reproduced from Schweitzer-Stenner[S6] with permission from the Royal Society of Chemistry.

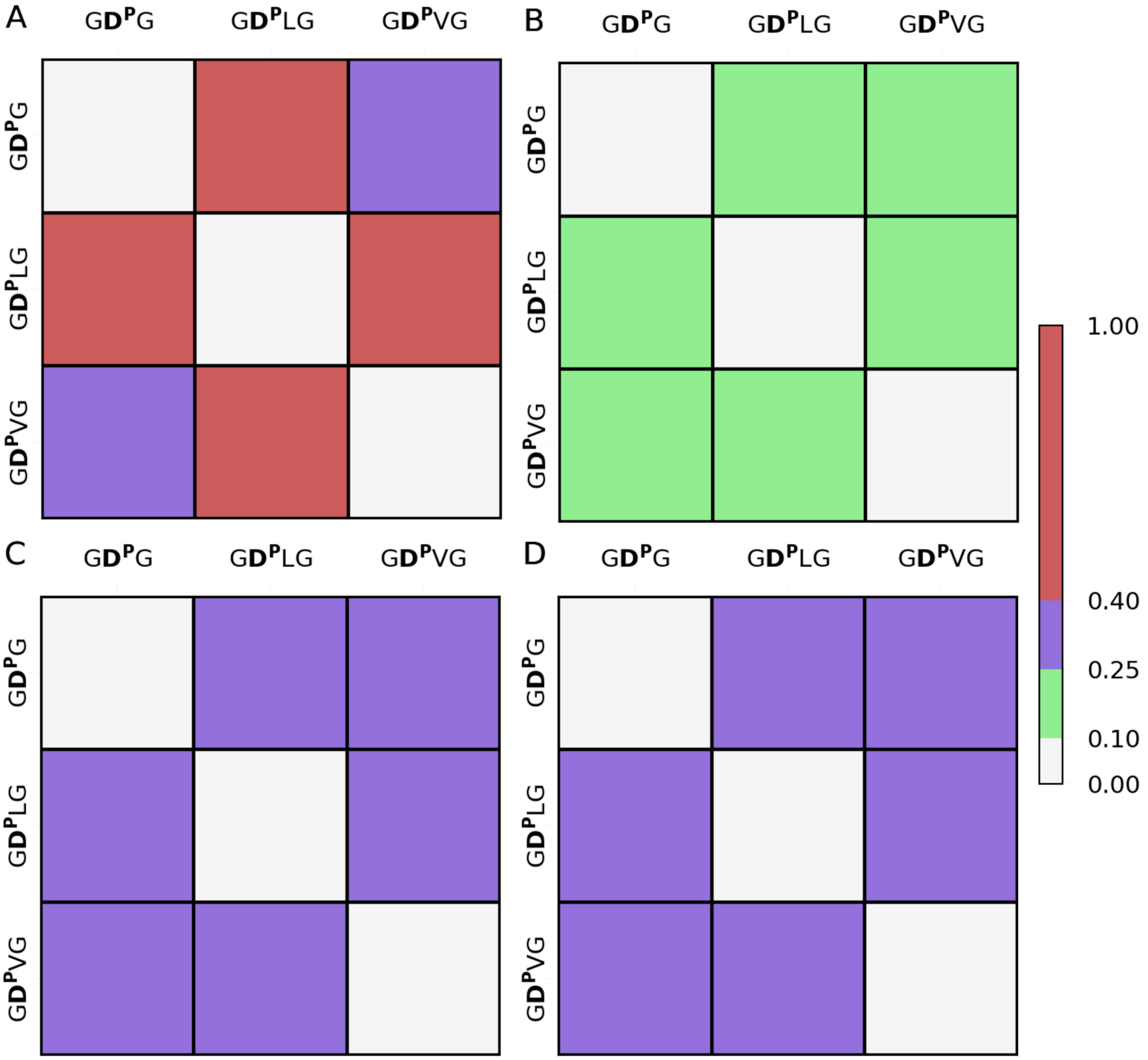

Figure S17: Hellinger distances between pairs of Ramachandran distributions of protonated aspartic acid residue in G**D**$^{\mathbf{P}}$G, G**D**$^{\mathbf{P}}$LG, and G**D**$^{\mathbf{P}}$VG peptides, derived from (A) the Gaussian model, (B) CHARMM36m, (C) Amber ff14SB, and (D) Amber ff24EXP-GA simulations. The red, purple, green, and white colors correspond to very dissimilar, moderately dissimilar, moderately similar, and very similar Ramachandran distributions, respectively. The Gaussian Ramachandran distributions for serine residue in G**D**$^{\mathbf{P}}$LG and G**D**$^{\mathbf{P}}$VG peptides used in this analysis are reproduced from Schweitzer-Stenner[S6] with permission from the Royal Society of Chemistry. The Gaussian and MD-derived Ramachandran distributions of protonated aspartic acid residue in GD^{P}G peptide used in this calculation are reproduced from Andrews et al.[S3] with permission from the Royal Society of Chemistry and from Suresh et al. (Copyright 2025 American Chemical Society),[S4] respectively.

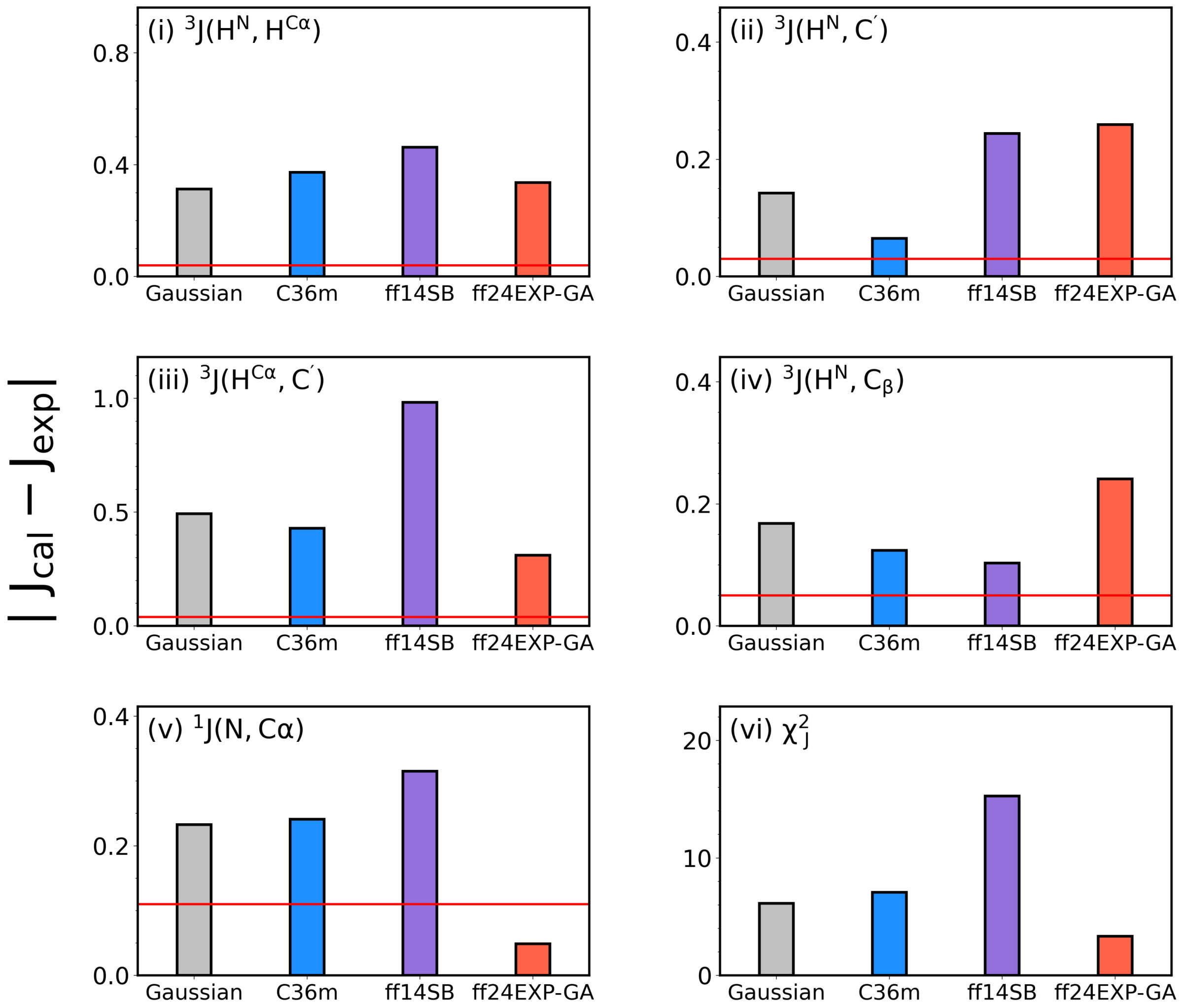

Figure S18: Comparison of experimental and computed J-coupling constants of protonated aspartic acid residue in G$\mathbf{D^P}$LG peptide. (i-v) Absolute differences between calculated and experimental values of the five J-coupling constants for the Gaussian model and the three MD force fields. Red lines correspond to experimental uncertainties. (vi) Reduced ${\chi^2}_J$ values. The Gaussian Ramachandran distribution used in this analysis is reproduced from Schweitzer-Stenner[S6] with permission from the Royal Society of Chemistry.

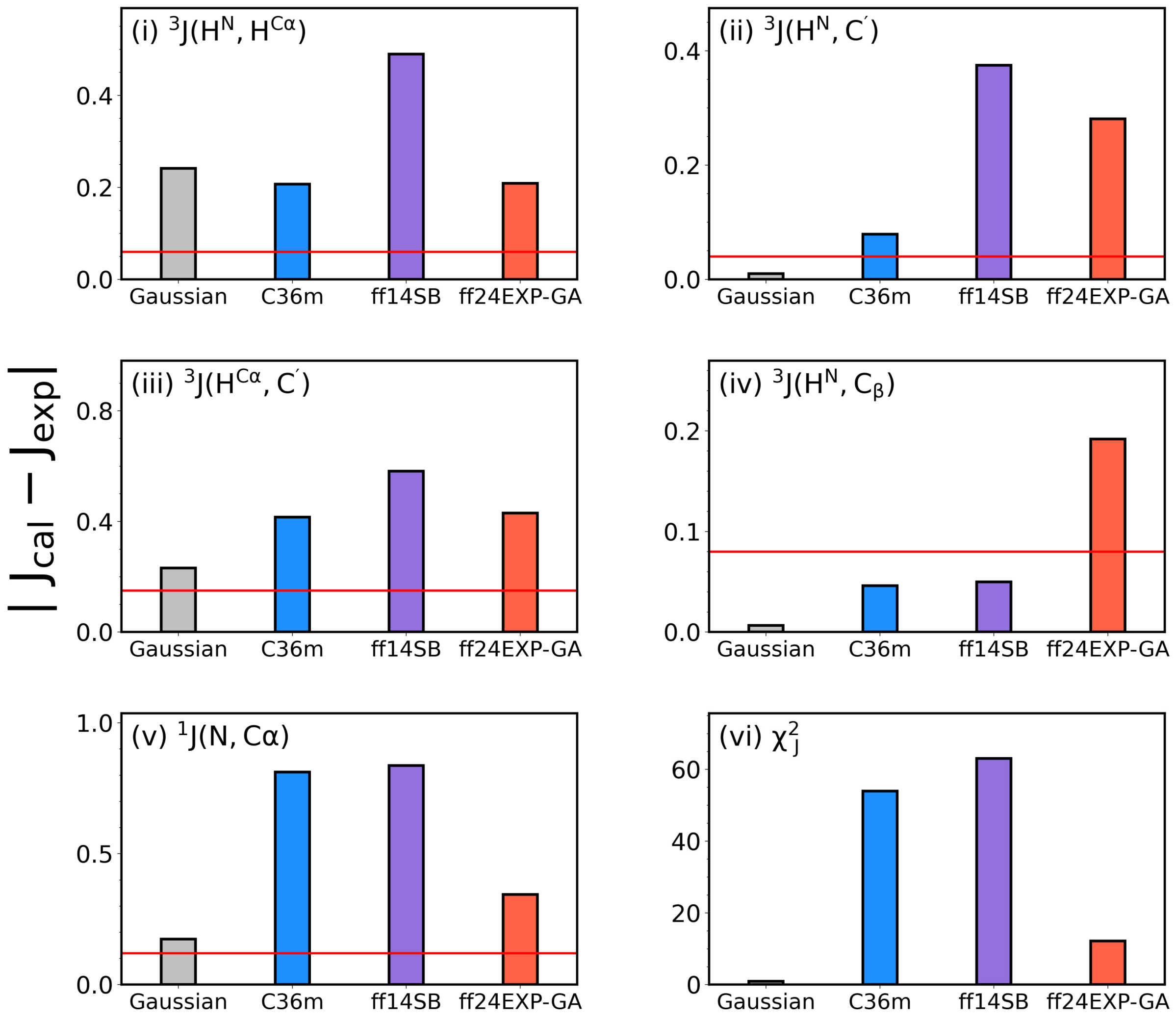

Figure S19: Comparison of experimental and computed J-coupling constants of protonated aspartic acid residue in G**D^P**VG peptide. (i-v) Absolute differences between calculated and experimental values of the five J-coupling constants for the Gaussian model and the three MD force fields. Red lines correspond to experimental uncertainties. (vi) Reduced ${\chi^2}_J$ values. The Gaussian Ramachandran distribution used in this analysis is reproduced from Schweitzer-Stenner[S6] with permission from the Royal Society of Chemistry.

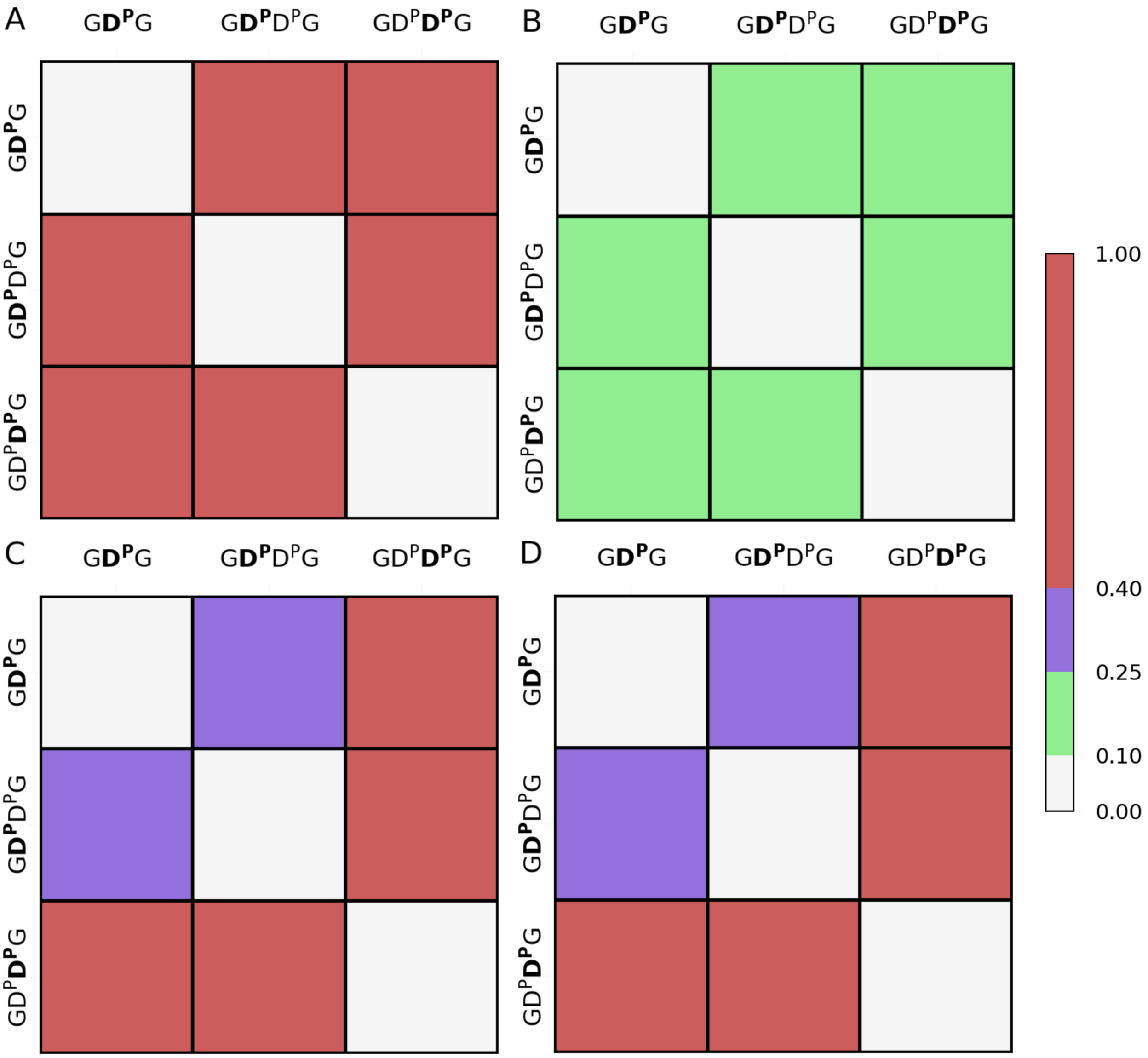

Figure S20: Hellinger distances between pairs of Ramachandran distributions of protonated aspartic acid residues in GD^{P}G and GD^{P}D^{P}G peptides, derived from (A) the Gaussian model, (B) CHARMM36m, (C) Amber ff14SB, and (D) Amber ff24EXP-GA simulations. The red, purple, green, and white colors correspond to very dissimilar, moderately dissimilar, moderately similar, and very similar Ramachandran distributions, respectively. The Gaussian Ramachandran distributions of aspartic acid residues in GD^{P}D^{P}G peptide used in this analysis are taken from Milorey et al. (Copyright 2021 American Chemical Society).[S5] The Gaussian and MD-derived Ramachandran distributions of protonated aspartic acid in GD^{P}G peptide used in this calculation are reproduced from Andrews et al.[S3] with permission from the Royal Society of Chemistry and from Suresh et al. (Copyright 2025 American Chemical Society),[S4] respectively.

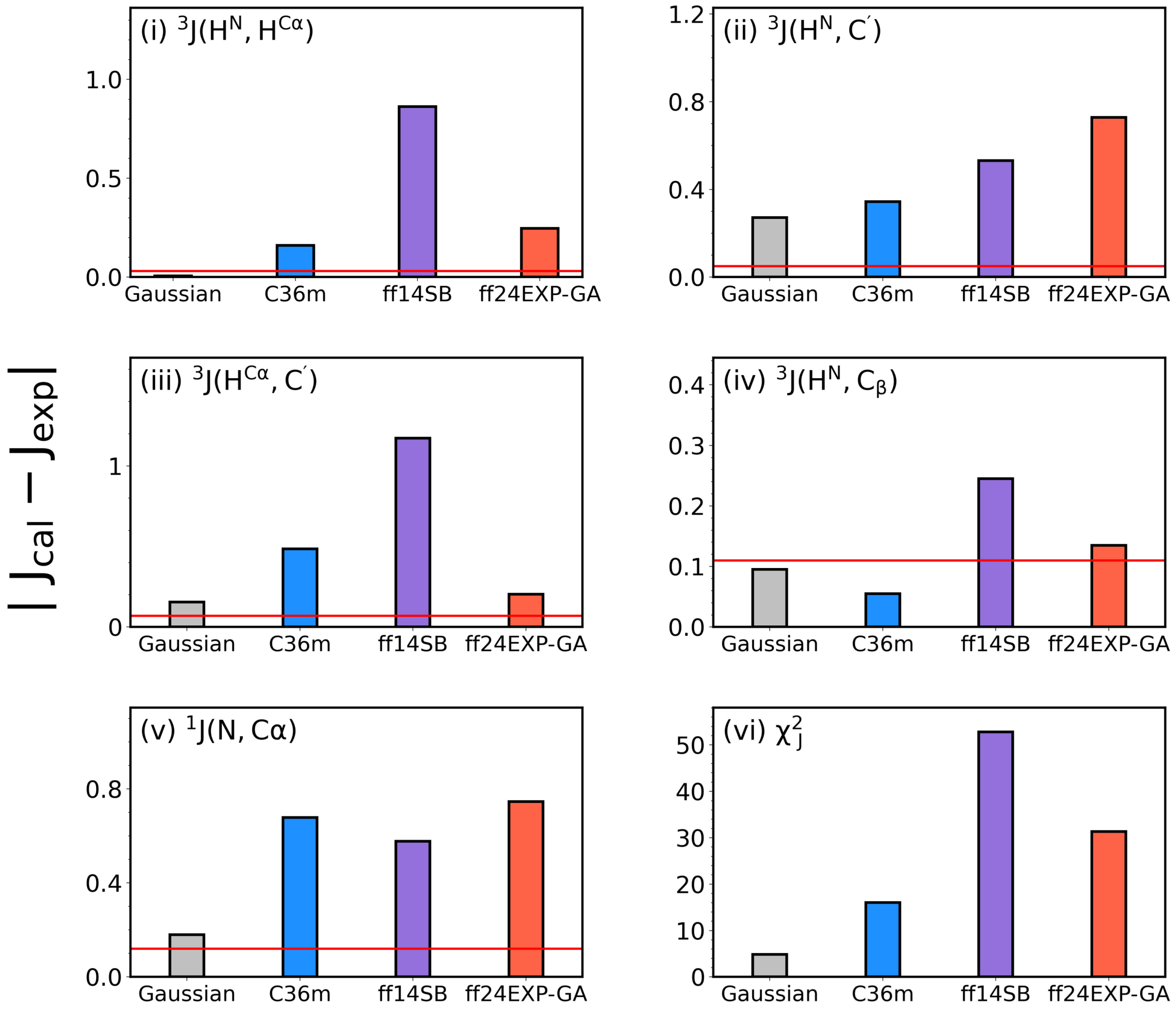

Figure S21: Comparison between experimental and computed J-coupling constants of the first protonated aspartic acid residue in G**D^P**D^PG peptide. (i-v) Absolute differences between calculated and experimental values of the five J-coupling constants for the Gaussian model and the three MD force fields. Red lines correspond to experimental uncertainties. (vi) Reduced $\chi^2{}_J$ values. The Gaussian Ramachandran distribution used in this calculation is taken from Milorey et al. (Copyright 2021 American Chemical Society).[S5]

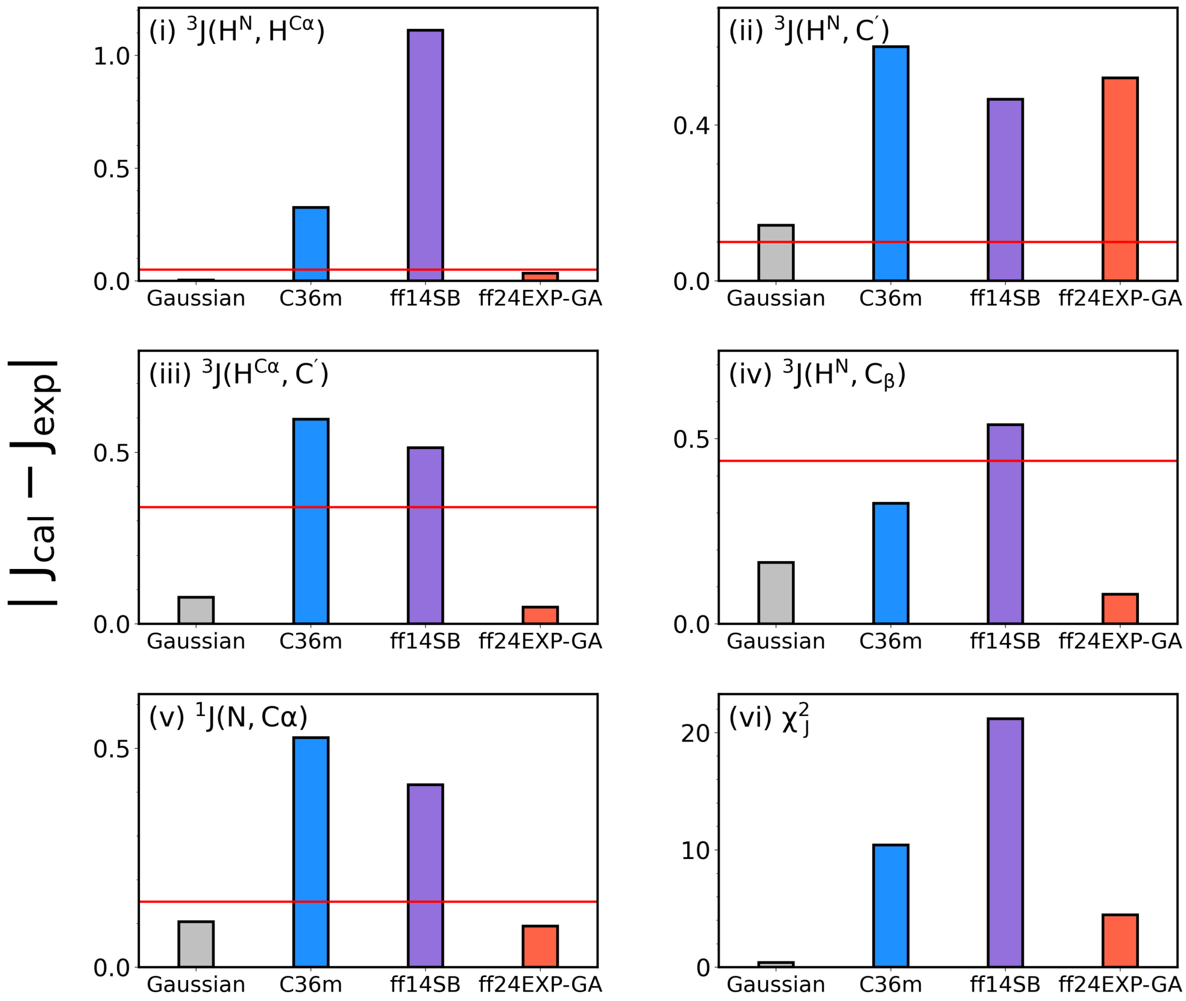

Figure S22: Comparison between experimental and computed J-coupling constants of the second aspartic acid residue in GD^{P}**D^{P}**G peptide. (i-v) Absolute differences between calculated and experimental values of the five J-coupling constants for the Gaussian model and the three MD force fields. Red lines correspond to experimental uncertainties. (vi) Reduced $\chi^2{}_J$ values. The Gaussian Ramachandran distribution used in this calculation is taken from Milorey et al. (Copyright 2021 American Chemical Society).[S5]

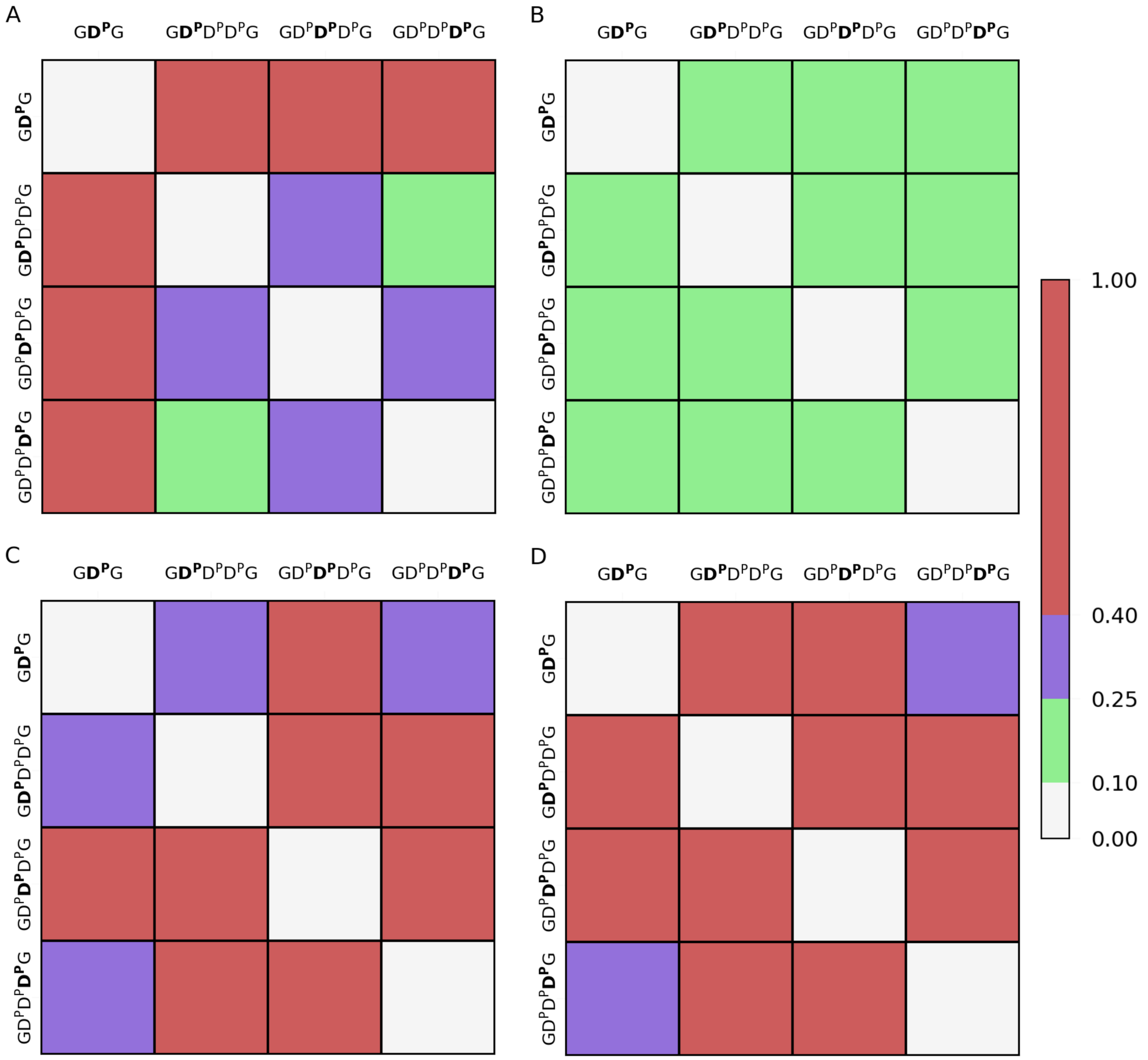

Figure S23: Hellinger distances between pairs of Ramachandran distributions of protonated aspartic acid residues in GD^{P}G and GD^{P}D^{P}D^{P}G peptides, derived from (A) the Gaussian model, (B) CHARMM36m, (C) Amber ff14SB, and (D) Amber ff24EXP-GA simulations. The red, purple, green, and white colors correspond to very dissimilar, moderately dissimilar, moderately similar, and very similar Ramachandran distributions, respectively. The Gaussian Ramachandran distributions for aspartic acid residues in GD^{P}D^{P}D^{P}G peptide used in this calculation are taken from Milorey et al.[S5] The Gaussian model and MD-derived Ramachandran distributions of protonated aspartic acid in GD^{P}G peptide are reproduced from Andrews et al.[S3] with permission from the Royal Society of Chemistry and from Suresh et al.,[S4] respectively.

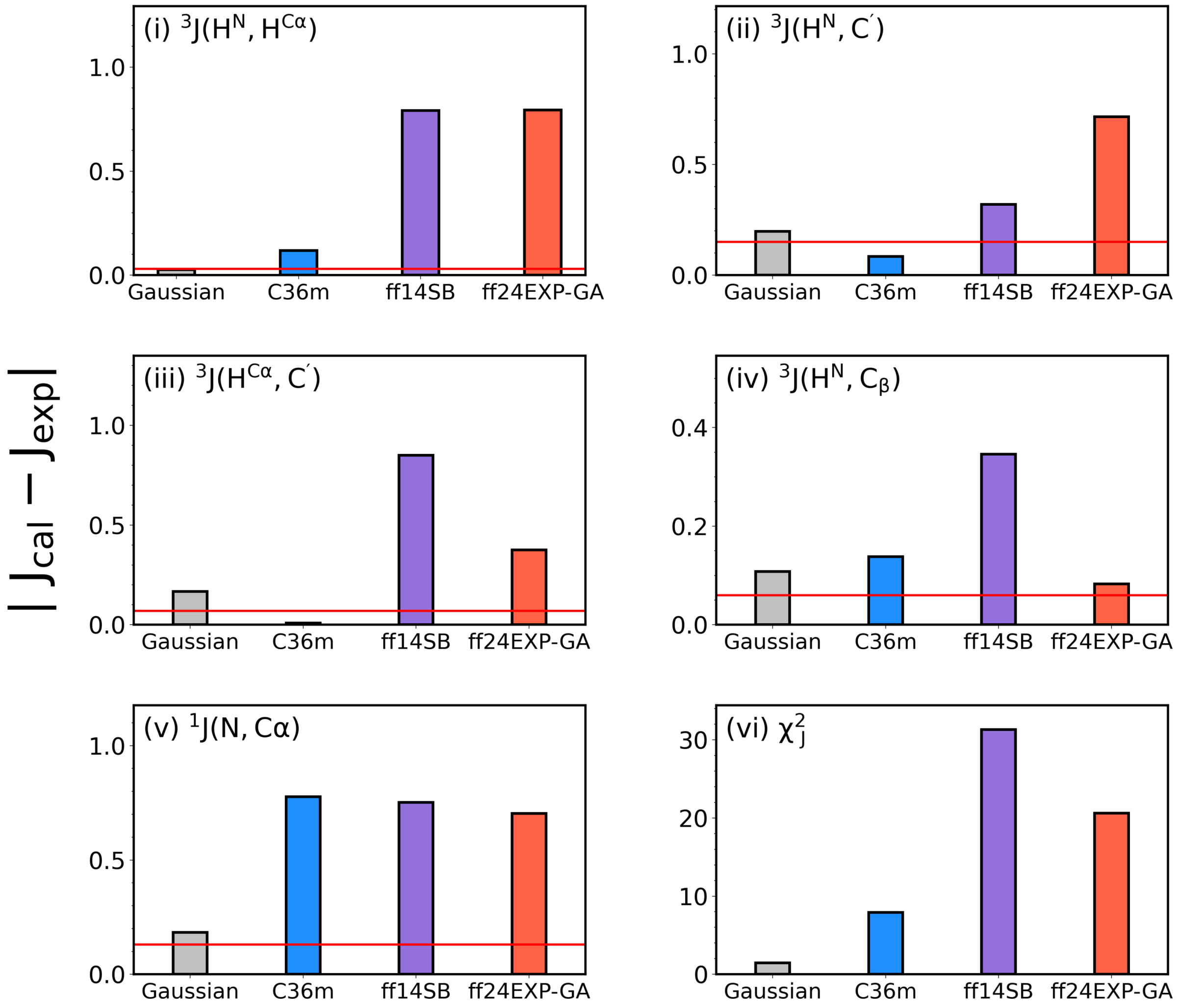

Figure S24: Comparison between experimental and computed J-coupling constants of the first protonated aspartic acid residue in G$\mathbf{D^P}$D^{P}D^{P}G peptide. (i-v) Absolute differences between calculated and experimental values of the five J-coupling constants for the Gaussian model and the three MD force fields. Red lines correspond to experimental uncertainties. (vi) Reduced $\chi^2{}_J$ values. The Gaussian Ramachandran distribution used in this calculation is taken from Milorey et al. (Copyright 2021 American Chemical Society).[S5]

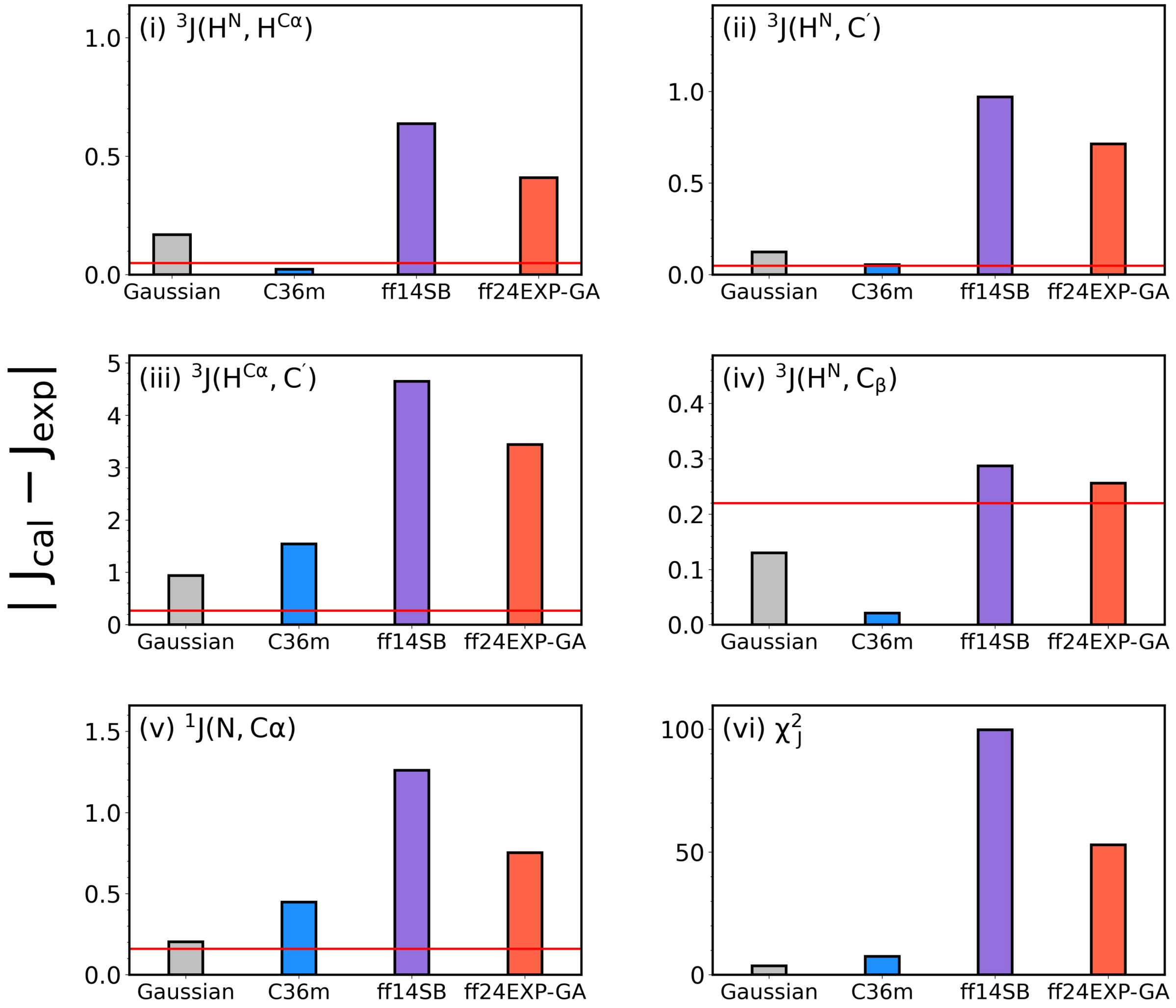

Figure S25: Comparison between experimental and computed J-coupling constants of the second protonated aspartic acid residue in $GD^{P}\mathbf{D^{P}}D^{P}G$ peptide. (i-v) Absolute differences between calculated and experimental values of the five J-coupling constants for the Gaussian model and the three MD force fields. Red lines correspond to experimental uncertainties. (vi) Reduced $\chi^2{}_J$ values. The Gaussian Ramachandran distribution used in this calculation is taken from Milorey et al. (Copyright 2021 American Chemical Society).[S5]

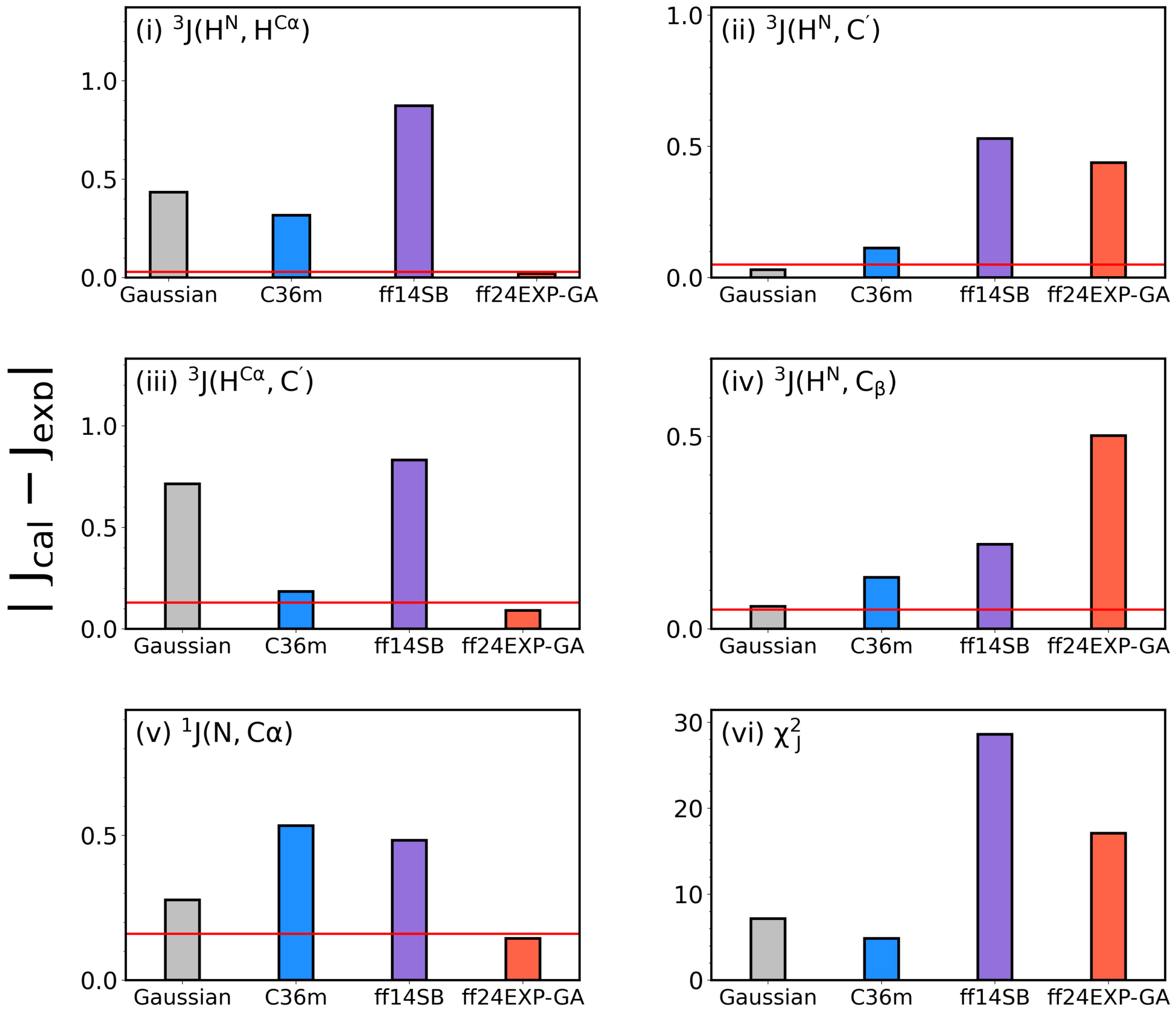

Figure S26: Comparison between experimental and computed J-coupling constants of the third protonated aspartic acid residue in GD^{P}D^P**D^P**G peptide. (i-v) Absolute differences between calculated and experimental values of the five J-coupling constants for the Gaussian model and the three MD force fields. Red lines correspond to experimental uncertainties. (vi) Reduced $\chi^2{}_J$ values. The Gaussian Ramachandran distribution used in this calculation is taken from Milorey et al. (Copyright 2021 American Chemical Society).[S5]

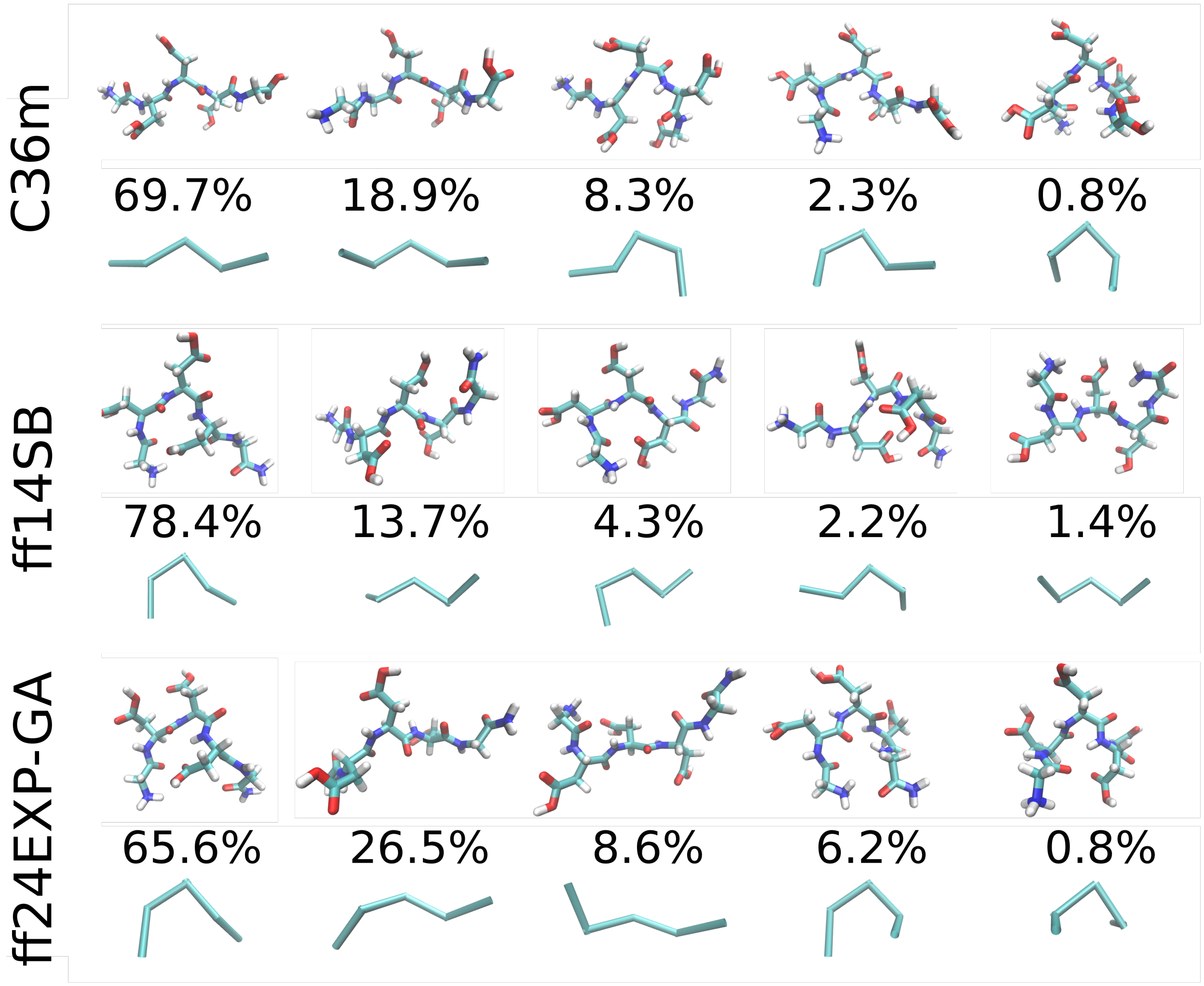

Figure S27: Populations of the predominant five clusters of GD^{P}D^{P}D^{P}G peptide conformations derived from CHARMM36m, Amber ff14SB and Amber ff24EXP-GA simulations. The GROMOS clustering algorithm by Daura et al.[S7] was used to generate clusters based on heavy (non-hydrogen) atom coordinates with a RMSD cut-off value of 0.2 nm, using conformations from 50-1000 ns of each respective trajectory.

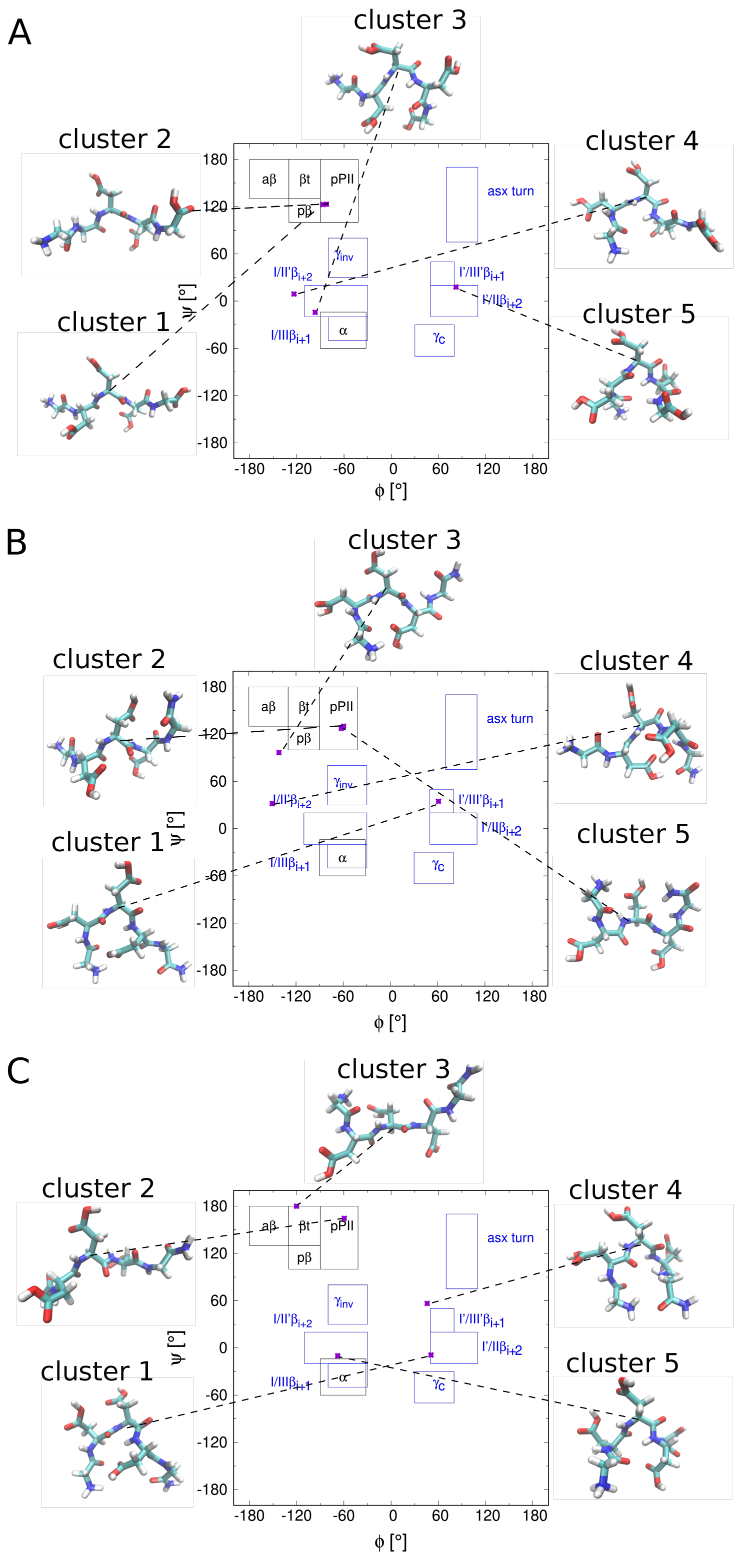

Figure S28: The the average dihedral angles for the central aspartic acid residue in GD^{P}D^{P}D^{P}G peptide for each of the five predominant clusters of conformations shown in Fig. S27 derived from (A) CHARMM36m, (B) Amber ff14SB, and (C) Amber ff24EXP-GA simulations.

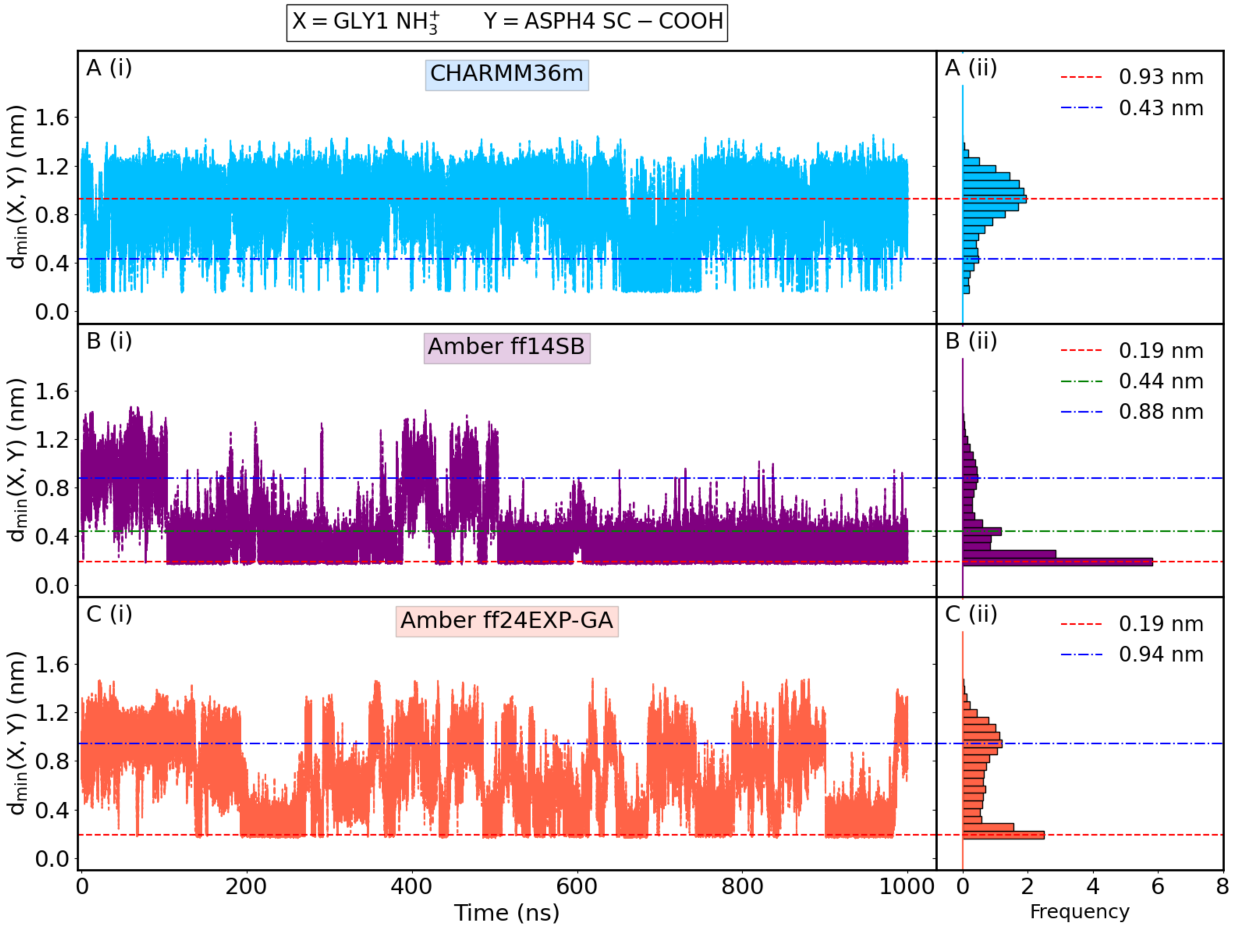

Figure S29: (i) Time evolution and (ii) histograms of the minimum distance, d_{min}, between NH_3^+ group of GLY1 and the side chain COOH group of ASPH4 in GD^{P}D^{P}D^{P}G peptide derived from (A) CHARMM36m, (B) Amber ff14SB, and (C) Amber ff24EXP-GA simulations. (ii) The most abundant distances, corresponding to the peaks of the respective histograms reported in legends, are marked by dashed orange, green, and blue lines.

References

(S1) Schweitzer-Stenner, R. Distribution of conformations sampled by the central amino acid residue in tripeptides inferred from amide I' band profiles and NMR scalar coupling constants. *J. Phys. Chem. B* **2009**, *113*, 2922–2932.

(S2) Zhang, S.; Schweitzer-Stenner, R.; Urbanc, B. Do molecular dynamics force fields capture conformational dynamics of alanine in water? *J. Chem. Theory Comput.* **2020**, *16*, 510–527.

(S3) Andrews, B.; Guerra, J.; Schweitzer-Stenner, R.; Urbanc, B. Do molecular dynamics force fields accurately model Ramachandran distributions of amino acid residues in water? *Phys. Chem. Chem. Phys.* **2022**, *24*, 3259–3279.

(S4) Suresh, A.; Schweitzer-Stenner, R.; Urbanc, B. Amber ff24EXP-GA, based on empirical Ramachandran distributions of glycine and alanine residues in water. *J. Chem. Theory Comput.* **2025**, *21*, 2515–2534.

(S5) Milorey, B.; Schwalbe, H.; O'Neill, N.; Schweitzer-Stenner, R. Repeating aspartic acid residues prefer turn-like conformations in the unfolded state: Implications for early protein folding. *J. Phys. Chem. B* **2021**, *125*, 11392–11407.

(S6) Schweitzer-Stenner, R. Nearest neighbour interactions between amino acid residues in short peptides and coil libraries. *RSC Adv.* **2026**, *16*, 17249–17266.

(S7) Daura, X.; Gademann, K.; Jaun, B.; Seebach, D.; van Gunsteren, W. F.; Mark, A. E. Peptide folding: when simulation meets experiment. *Angew. Chem. Int. Ed.* **1999**, *38*, 236–240.
